# Upgrading Ion Migration and Interface Chemistry via a Cyano-Containing COF in a Single-Ion Conductive Polymer toward High-Voltage Lithium–Metal Batteries

**DOI:** 10.1021/jacs.5c08267

**Published:** 2025-07-11

**Authors:** Xiaosa Xu, Junjie Chen, Jin Li, Jiadong Shen, Pengzhu Lin, Zhenyu Wang, Zixiao Guo, Jing Sun, Baoling Huang, Tianshou Zhao

**Affiliations:** † Department of Mechanical and Aerospace Engineering, 58207 The Hong Kong University of Science and Technology, Clear Water Bay, Kowloon 999077, Hong Kong SAR, China; ‡ Department of Mechanical and Energy Engineering, Southern University of Science and Technology, Shenzhen 518055, China

## Abstract

Concentration polarization-triggered dendrite growth hinders the practical application of solid-state polymer lithium batteries, which is caused by the uncontrolled anion migration in conventional dual-ion electrolytes. Single-ion conductive polymer electrolytes (SICPEs) offer a promise to mitigate dendrite growth via reducing concentration polarization and prohibiting salt depletion, yet they are highly challenging for successful implementation due to their narrow electrochemical window and poor ionic conductivity, which result from the deficient dissociation of Li^+^ polyanions and sluggish chain relaxation. Here, a cyano-containing covalent organic framework (COF) is designed to fuse with SICPEs, promising fast Li^+^ transport and remarkable interfacial stability toward high-voltage lithium–metal batteries. The electron-withdrawing cyano group on the COF facilitates the dissociation of the polyanions via ion–dipole interactions, resulting in more free-moving Li^+^. Rapid ion migration then occurs along the long-range cooperative ion transport pathways between the COF and SICPE. Additionally, the cyano group robustly bonds with transition metal ions of NCM cathodes to inhibit the catalytic decomposition of SICPE and guarantee the structural integrity of NCM. Hence, the as-prepared SICPE exhibits a significantly enhanced ionic conductivity of 9.2 × 10^–4^ S cm^–1^ and an improved Li^+^ transference number of 0.94 at room temperature. Accordingly, the NCM622||Li quasi-solid-state cell achieves an exceptional capacity retention of 92.0% over 1000 cycles at 0.5 C, while the cell pairing with the 4.8 V NCM622 cathode delivers a remarkable capacity of 149.5 mAh g^–1^ after 200 cycles at 0.5 C. This study provides a new perspective for facilitating ionic conductivity and interface chemistry toward the practical feasibility of single-ion conductive polymer electrolytes.

## Graphical abstract

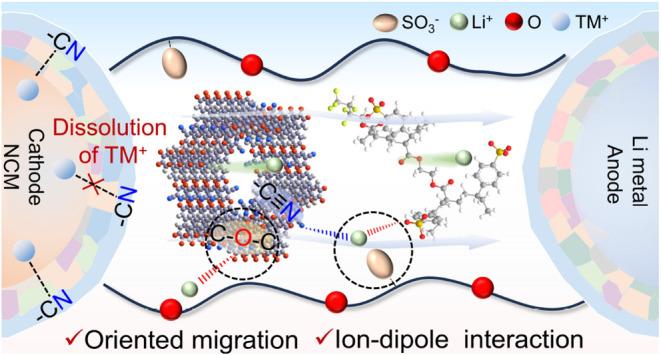

## Introduction

Solid-state lithium metal batteries (SSLMBs) consisting of solid-state electrolytes (SSEs) and Li metal anodes are promising candidates for next-generation high-specific-energy batteries.^1^ Among different types of SSEs, solid-state polymer electrolytes (SPEs) have sparked surging interest due to their excellent flexibility, processability, and interfacial compatibility.^2^ Typically, SPEs are mainly dual-ion conductors with lithium salts (e.g., LiFSI, LiTFSI, and LiPF_6_) dissolved in a polymer matrix (e.g., poly­(ethylene oxide) (PEO) and poly­(vinylidene fluoride-*co*-hexafluoropropylene) (PVDF-HFP)); their Li^+^ conductivity (σ_Li^+^_) has been improved through compositing with various fillers and designing new polymer chains.^3^ Nevertheless, the low Li^+^ transference number (*t*_Li^+^_ < 0.4) and narrow electrochemical window still remain persistent obstacles to their practical implementations.^4^ In particular, the free-moving anions in the traditional dual-ion SPEs trigger large concentration polarization and a reversed electric field upon cycling, which leads to enlarged overpotentials and uncontrolled lithium dendrite growth.^5^

The anion migration in the electric field causes the formation of a space charge region near the metal deposition side. According to the space charge theory proposed by Chazalviel et al., when the interface electric field reaches one critical value as the depletion of anions, Li^+^ deposits unevenly on the lithium–metal surface; afterward, the increased electric field near a growing tip accelerates the deposition rate and fast growth of the tip.^6,7^ Typically, the front of lithium dendrites expands at a velocity *v*_a_ = −μ_a_ *E*_0_ (where μ_a_ is the motion rate of the anion and *E*_0_ is the electric field intensity). Therefore, restricting the anion migration to improve the Li^+^ transference number is an effective strategy to inhibit the growth of lithium dendrites caused by space charge.

Single-ion conductive polymer electrolytes (SICPEs), featured by anions covalently bound onto the polymer skeletons to realize high *t*_Li^+^_ (close to unity), are ideal SPEs to diminish the undesired concentration polarization and dendrite growth.^7,8^ Although demonstrating enormous potential in SSLMBs, poor σ_Li^+^_ (∼10^–7^–10^–5^ S cm^–1^) at room temperature (RT) caused by inadequate ion dissociation and sluggish chain relaxation greatly impedes their practical application.^9,10^ Therefore, numerous efforts have been exerted to improve the σ_Li^+^_, such as designing polyanions with conjugated structures,^11^ constructing block copolymer architectures,^12^ and introducing ionic conductive groups into SICPEs.^13^ For example, Li et al. fabricated an interpenetrating SICPE based on [B­(C_6_F_4_)_4_]^−^ anions with proper O–Li^+^ coordination, yielding an enhanced σ_Li^+^_ of 3.53 × 10^–4^ S cm^–1^ and a *t*_Li^+^_ of 0.92 at RT.^5^ He et al. prepared a SICPE by the copolymerization of trifluoromethane sulfonimide lithium methacrylate and ethylene acrylate, delivering an improved σ_Li^+^_ of 6.3 × 10^–5^ S cm^–1^ and a *t*_Li^+^_ of 0.85 at RT.^14^ Nonetheless, compared with traditional dual-ion SPEs, it is more challenging to increase the ionic conductivity of SICPEs over 5.0 × 10^–4^ S cm^–1^ due to their high dissociation energy barrier of Li^+^ polyanions and the lack of effective ion migration pathways.^14,15^ Another major bottleneck of SICPEs is their deficient stability toward oxidation. Particularly, SICPEs with common ether-based functional groups fail to pair with high-energy cathodes such as ternary nickel cobalt manganese oxides (NCM).^9^ Therefore, exploring effective strategies to simultaneously regulate the Li^+^ migration and enhance oxidative stability is urgently desired for developing practical, feasible SICPEs.

Herein, an innovative design of fusing a cyano-containing covalent organic framework (COF) with SICPEs is devised to improve their ion transport and interfacial stability toward high-voltage quasi-solid-state lithium metal batteries. This functional SICPE is featured by incorporating a sulfonated lithium (−SO_3_Li)-containing SICPE with a cyano-containing COF316 framework. Figure 1a illustrates the regulation mechanisms of COF316 on the ionic migration and interfacial chemistry of SICPE. The strong electron-withdrawing cyano group (−C≡N) on the COF316 framework effectively reduces the dissociation energy barrier of Li^+^ polyanions via ion–dipole interactions of −C≡N···Li^+^, giving rise to more “free” Li^+^. The confinement of SICPE chains within/around COF pores establishes continuous ion transport pathways along their interface, enabling a rapid Li^+^ migration through the abundant ether-oxygen hopping sites in both COF316 and SICPE. Furthermore, the cyano group robustly bonds with transition metal (TM) ions of NCM cathodes to inhibit the catalytic decomposition of SICPE and guarantee the structural integrity of NCM cathodes upon cycling.^16^ In addition, the reduction of the cyano group and 2,2,3,4,4,4-hexafluorobutyl acrylate (HFBA) segment generates F, N-rich electrolyte/electrode interfacial films, promoting the fast Li^+^ transport and dense Li deposition. As a result, the as-prepared SICPE showcases a significantly enhanced Li^+^ conductivity (σ_Li^+^_ ∼ 9.2 × 10^–4^ S cm^–1^, *t*_Li^+^_ ∼ 0.94) and a wide electrochemical window up to 5.1 V. When pairing with the LiNi_0.6_Co_0.2_Mn_0.2_O_2_ (NCM622) cathode, a reversible capacity of 139.4 mAh g^–1^ with an exceptional capacity retention of 92.0% over 1000 cycles is obtained at 30 °C, positioning it among the top-performing SICPEs. Additionally, a remarkable capacity of 149.5 mAh g^–1^ after 200 cycles at 0.5 C can be achieved with a 4.8 V NCM622 cathode. Furthermore, the positive effects of the cyano-containing COF on SICPE were revealed via systematic characterization combined with theoretical calculations. The proposed optimization design for SICPEs provides a promising avenue for developing high-performance SSLMBs.

**Figure 1:**
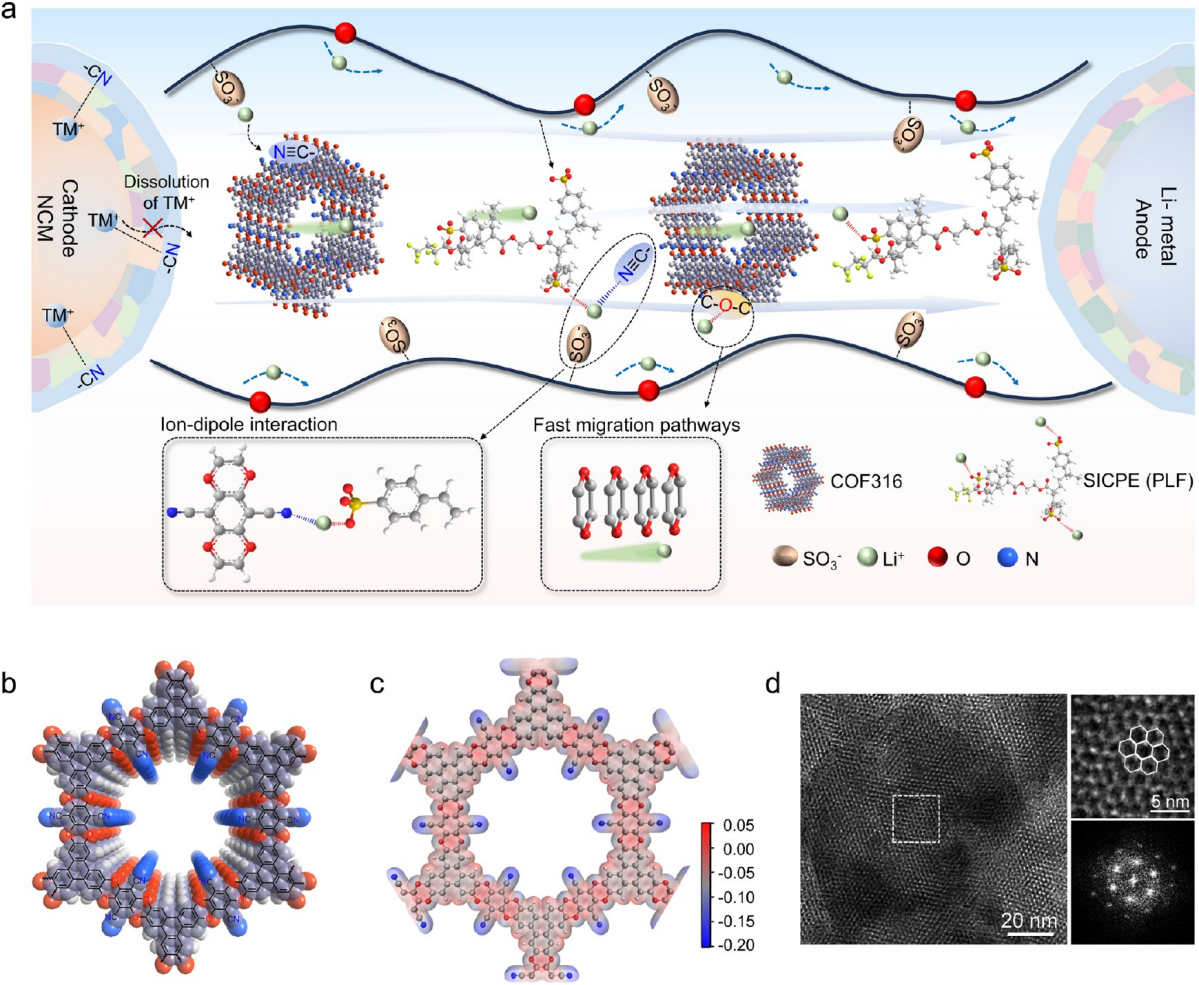
(a) Regulation mechanisms of COF316 on the ionic migration and interfacial chemistry of SICPEs. (b) Top view of the space-filling model, (c) the electrostatic potential (ESP), and (d) high-resolution transmission electron microscopy (TEM) images and corresponding selective area electron diffraction (SAED) pattern of COF316.

## Results and Discussion

### Synthesis of PLF@COF316 SICPE

The crystalline framework of COF316 with abundant cyano groups, excellent chemical stability, and well-ordered pores was synthesized by linking 2,3,6,7,10,11-hexahydroxytriphenylene (HHTP) and tetrafluoroterephthalonitrile (TFTPN) monomers (Figures 1b and S1).^17^ Electrostatic potential (ESP) of materials significantly influence the ion migration behavior, with lower potentials typically promoting electrophilic migration. As shown in Figure 1c, COF316 exhibits numerous negative potentials around the electron-withdrawing groups of cyano groups within the overall framework, which is responsible for manipulating electron distribution and expediting Li^+^ migration.^18^ Specifically, the most negative potential sites around cyano groups are preferential to attract the Li^+^ on SICPE segments via electrostatic interaction, facilitating the dissociation of Li^+^ polyanions. Furthermore, the −O– groups on the COF316 framework with moderate negative potential serve as a “bridge” for Li^+^ transport at the interface between the polymer chain and the COF. The highly ordered two-dimensional (2D) hexagonal channels can be clearly observed from the high-resolution transmission electron microscopy (TEM) images (Figure 1d) and top view of the simulated COF316 framework (Figure 1b). The hexagonal pore size is ∼1.2 nm along the pore axes, which can serve as a desirable “reservoir” to accommodate SICPE chains. The selective area electron diffraction (SAED) pattern with a regular array demonstrates the exceptional crystallinity of as-prepared COF316.

As depicted in Figures 2a and S2, a COF316-fused SICPE membrane was prepared by the copolymerization of poly­(ethylene glycol) diacrylate (PEGDA), lithium *p*-styrenesulfonate (SSLi), and HFBA within/around COF316 under ultraviolet (UV) radiation (named PLF@COF316). As a control, the pure polymer chain was also prepared by the copolymerization of PEGDA, SSLi, and HFBA without COF316 (named PLF). First, the structure and chemical environment of COF316 were analyzed in detail. The powder X-ray diffraction (XRD) pattern in Figure 2b shows three diffraction peaks at 4.3, 8.5, and 26.6°, corresponding to the (100), (200), and (001) planes of COF316, respectively. The formation of the characteristic dioxin C–O asymmetric (1262 cm^–1^) and symmetric (1024 cm^–1^) stretching modes of COF316 was verified by Fourier transform infrared (FT-IR) spectra (Figure S3). The vibrating peak belonging to cyano groups can also be found at 2241 cm^–1^. The solid-state ^13^C nuclear magnetic resonance (NMR) spectrum further validated the formation of COF316 (Figure S4). The resonance signals at 147.3 and 139.6 ppm are typical of C–O carbons. The peak at 95.7 ppm is attributed to aromatic carbons connected to nitriles, while those at 127.2 and 111.6 ppm are identified as nitrile and aromatic carbons. The Brunauer–Emmett–Teller (BET) surface area of COF316 was calculated to be 337.9 m^2^ g^–1^, accompanied by a narrow pore size of 1.2 nm (Figure 2c and Table S1), consistent with the TEM image. The high-angle annular dark-field scanning transmission electron microscopy (HAADF-STEM) image further clearly demonstrates the well-ordered hexagonal channels of COF316. The corresponding energy-dispersive spectroscopy (EDS) mapping exhibits the homogeneous distribution of C, N, and O elements throughout COF316 (Figure S5).

**Figure 2:**
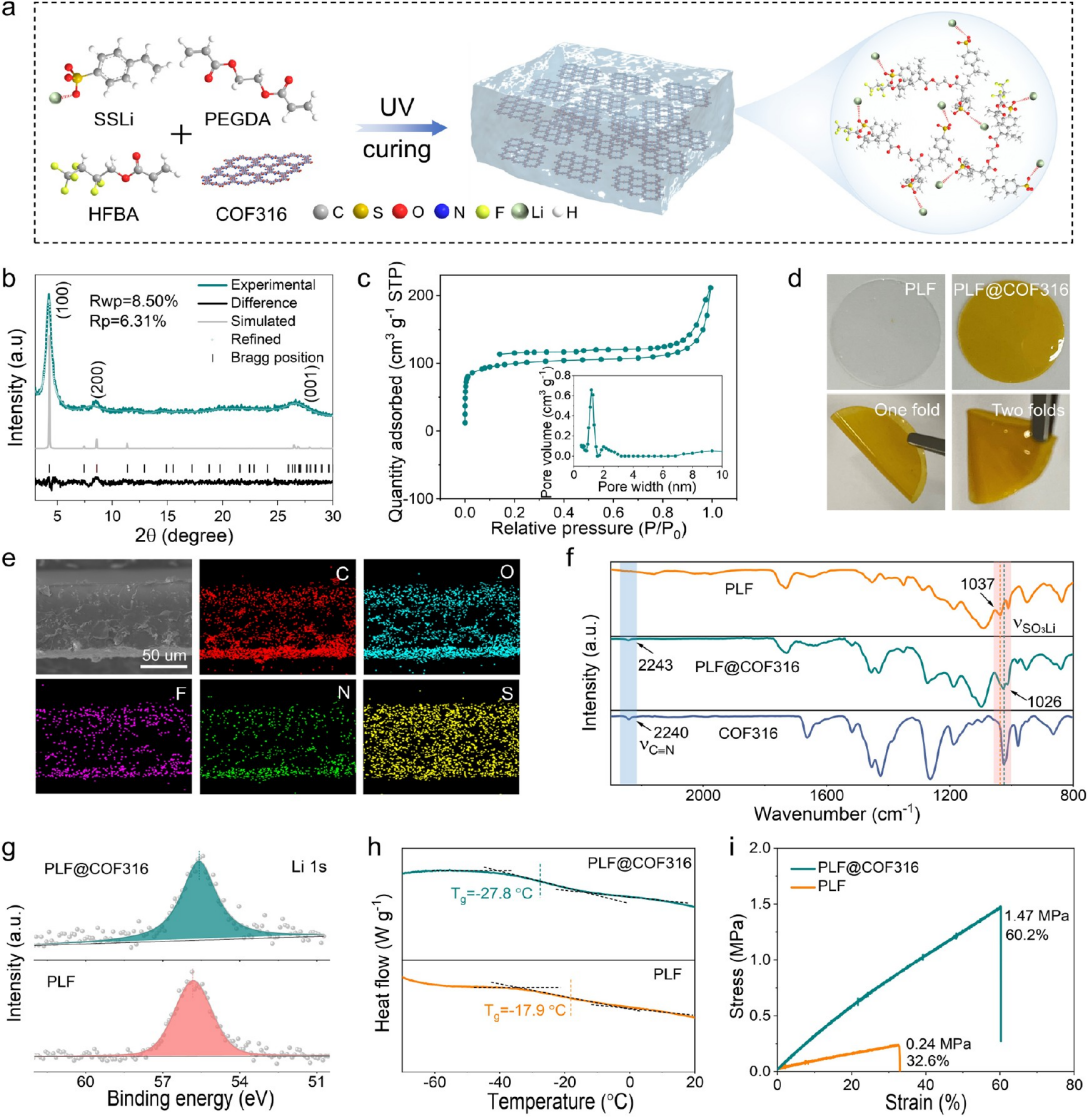
Synthesis of the PLF@COF316 electrolyte. (a) The preparation of the PLF@COF316 electrolyte. (b) XRD spectrum and (c) nitrogen adsorption–desorption isotherms of COF316. (d) Digital photographs for illustrating the flexibility of PLF@COF316. (e) Cross-sectional SEM image and corresponding EDS element mappings of PLF@COF316. (f) FT-IR spectra of COF316, PLF, and PLF@COF316. (g) X-ray photoelectron spectroscopy (XPS) Li 1s spectra, (h) glass transition temperatures (*T*_g_), and (i) stress–strain curves of PLF and PLF@COF316.

Various measurements were further conducted to explore the as-prepared PLF@COF316 electrolyte membrane. The scalable PLF@COF316 film with an area of ∼500 cm^2^ was prepared through photopolymerization (Figure S6), indicating the superior processing performance of the PLF@COF316 electrolyte. As displayed in Figure 2d, the SICPE membrane without COF316 turns from colorless to yellow after introducing COF316. No cracking or damage occurs after both one and two folds, indicative of its excellent mechanical strength. The cross-sectional SEM image shows that the thickness of the PLF@COF316 membrane is ∼75 μm, and the corresponding EDS mapping exhibits the homogeneous distribution of C, O, F, N, and S elements throughout PLF@COF316 (Figure 2e). FT-IR was employed to survey the polymerization reactions and interactions between PLF and COF316. The C=C absorption peaks of SSLi, PEGDA, and HFBA disappeared after polymerization, indicating that the monomers were successfully polymerized into a cross-linking structure (Figure S7).^19^ The gel permeation chromatography (GPC) analysis shows that the average molecular weight (Mn) of the synthesized PLF is 157,893 g mol^–1^ (Figure S8 and Table S2). Furthermore, the liquid-state ^1^H NMR spectrum shows chemical shifts at 7.6, 7.4, and 1.8 ppm, which are attributed to SSLi, while those at 3.5 and 3.3 ppm are ascribed to PEGDA and HFBA, respectively (Figure S9). These typical signals in PLF@COF316 indicate the successful polymerization of monomers to form the PLF polymer chain. The XRD pattern in Figure S10 shows an amorphous state of PLF@COF316, which is favorable to promoting the segmental mobility and Li^+^ transport in the electrolyte.^20^ In addition, the obvious shift of −SO_3_Li stretching compared to PLF (from 1037 to 1026 cm^–1^) and −C≡N absorption band compared to that of COF316 (from 2240 to 2243 cm^–1^) in PLF@COF316 indicates a robust ion–dipole interaction between PLF and COF316 (Figure 2f). Furthermore, due to the ion–dipole interaction between Li^+^ and −C≡N, the X-ray photoelectron spectroscopy (XPS) Li 1s orbital in PLF@COF316 suffers an evident downshift after the introduction of COF316 (Figure 2g). Differential scanning calorimetry (DSC) was employed to assess the segmental motion of the PLF chain. As shown in Figure 2h, the introduction of COF316 decreases the glass transition temperature (*T*_g_) of PLF from −17.9 to −27.8 °C, which is conducive to enhancing the polymer segmental mobility and decreasing the energy barrier for Li^+^ migration.^21^ The stress–strain curves in Figure 2i exhibit that PLF@COF316 bears higher mechanical strength and stretchability than PLF, which may be attributed to the nanochannel confinement and strong interactions between PLF and COF316.^22^ Thermogravimetric analysis (TGA) was employed to assess the thermal stability of the electrolytes. As shown in Figure S11, PLF@COF316 and PLF show a high decomposition temperature of ∼358 °C, indicating their excellent thermal stability. In addition, the PLF@COF316 membrane is almost nonflammable after the ignition test, while the PLF membrane was ignited and burned out (Figure S12), indicating the excellent fire resistance of PLF@COF316, which is conducive to the secure operation of SSLMBs.^23^ We further explore the mechanical thermodynamic properties of PLF@COF316 by the stress–strain curves at high temperatures (Figure S13). As the temperature increases to 150 °C, the tensile strength of the PLF@COF316 membrane gradually decreases, while the elongation at break increases. This phenomenon is attributed to the intensified thermal motion of polymer chains at elevated temperatures, which weakens intermolecular forces, thereby reducing tensile strength and improving breaking elongation.

### Analysis of Li^+^ Migration in PLF@COF316 SICPE

The ESP distribution is simulated to reveal the local interaction between COF316 and the PLF polymer chain. As shown in Figure 3a, an obvious negative potential (blue zone) can be found in the −C≡N sites of COF316, while the positive potential (red area) appears in the Li sites of the SSLi segment. In the SSLi-COF316 configuration, an obvious blue shift of the Li atom in SSLi indicates a decreased electron density, while the −C≡N sites of COF316 show an elevated degree of positive zones, showcasing a robust ion–dipole interaction between COF316 and the sulfonated Li (−SO_3_^–^Li^+^) group.^24^ Deformation charge density was performed to further reveal the mechanism of enhanced Li^+^ dissociation. As depicted in Figure 3b, massive electron concentration in −C≡N sites of COF316 and dissipation in the Li sites of SSLi reveal a spontaneous electron transfer from the SSLi segment to COF316, highlighting that the Li^+^ is bound with −C≡N via ion–dipole interaction and easily dissociated from the −SO_3_^–^Li^+^ group.^25^ Quantitatively, the dissociation energy barrier of Li^+^ from the −SO_3_^–^Li^+^ group is calculated to be 6.59 eV, which decreases to 5.35 eV after introducing the COF316 framework (Figure 3c), indicating that the interaction between Li^+^ and −SO_3_^–^ is weakened when Li^+^ is attracted by the −C≡N sites on the COF316 framework via ion–dipole interaction. The much-reduced Li^+^ dissociation energy barrier in the SSLi-COF316 configuration suggests that more free-moving Li^+^ can be dissociated from the PLF polymer chain, thereby enhancing Li^+^ transport.

**Figure 3:**
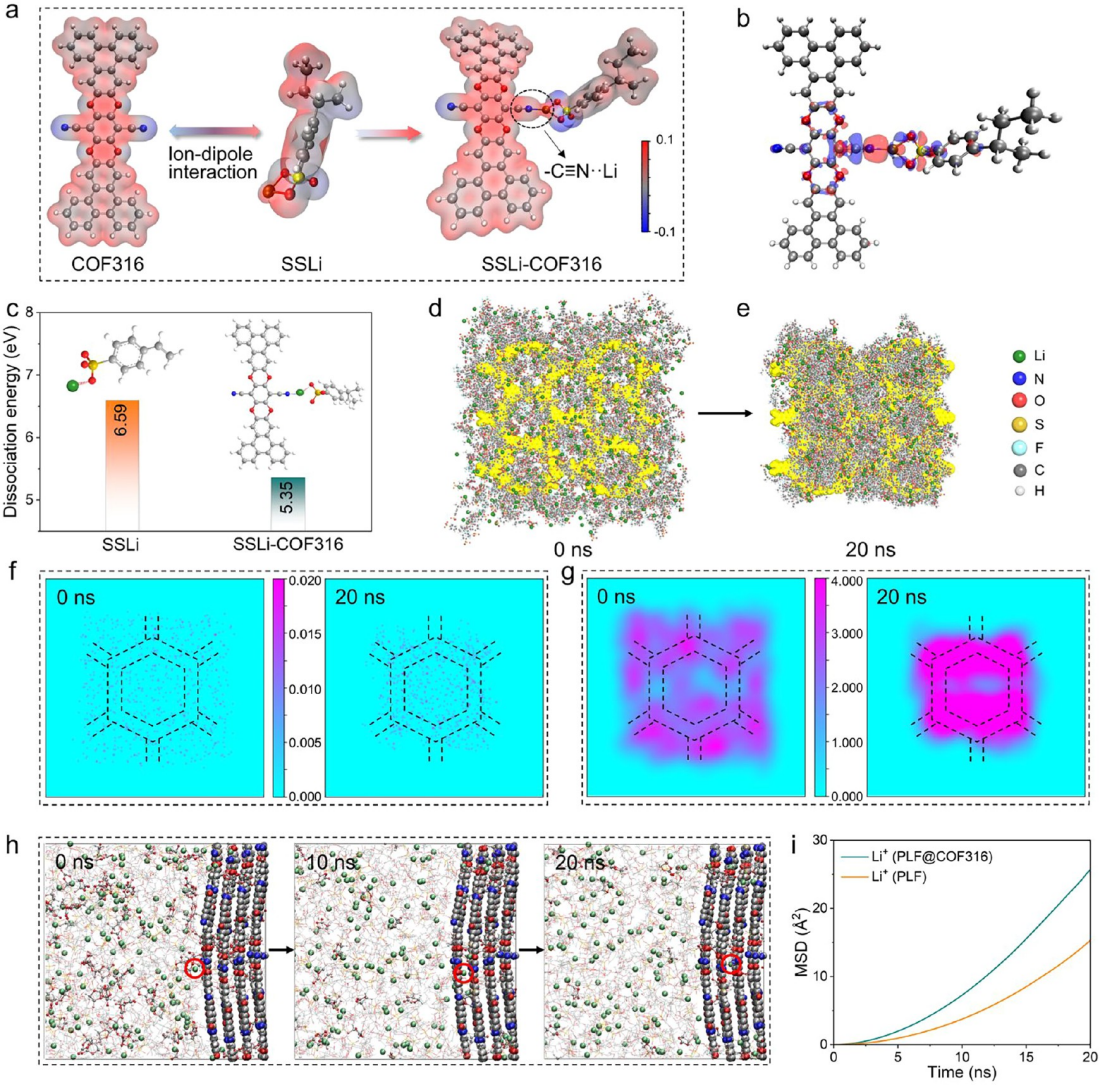
Simulation of the Li^+^ migration in PLF@COF316. (a) Electrostatic potential calculations and (b) deformation charge density of the SSLi-COF316 structure. (The red and blue clouds represent the electron concentration and dissipation area, respectively.) (c) Calculated Li^+^ dissociation energy barriers of SSLi and SSLi-COF316 configurations. The top view of the conformation evolution of the PLF@COF316 system at (d) 0 ns and (e) 20 ns based on molecular dynamics (MD) simulations. 2D number density distribution of (f) Li^+^ and (g) PLF near COF316. (h) Simulation snapshots of the Li^+^ migration in the PLF@COF316 system at room temperature. (i) The mean-squared displacement (MSD) of Li^+^ in the PLF@COF316 and PLF systems calculated from MD simulations.

The initial molecular dynamics (MD) simulations were conducted to deeply evaluate the Li^+^ diffusion kinetics in both electrolytes. As shown in Figures 3d and S14a, the PLF chains are homogeneously distributed in the box, while Li^+^ coordinates uniformly with the polymer segments at the initial stage (0 ns) of MD-simulated PLF@COF316. However, the local ionic environment is significantly altered, displaying an uneven distribution with notable accumulation in some specific regions after simulation for 20 ns (Figures 3e and S14b), which attributes to the fact that the high density of electron-withdrawing cyano groups on COF316 could attract the Li^+^ in PLF chains by robust ion–dipole interaction of −C≡N···Li^+^.^26^ 2D number density distribution images depict that Li^+^ exhibits substantial accumulation at the COF sites in PLF@COF316 after 20 ns (Figure 3f), highlighting a strong affinity for Li^+^ sites facilitated by plentiful electronegative channels in COF316. Furthermore, the polarized state of the open channels in COF316 promotes the encapsulation of polar guest components, resulting in the spatial confinement of PLF chains within the pores of COF316 (Figure 3g), which is conducive to establishing an accelerated conductive pathway for Li^+^ migration.^27^ The simulation snapshots are captured to track the Li^+^ conductivity in both electrolytes. In the PLF@COF316 system, Li^+^ ions are evenly distributed throughout the interconnected structure of PLF chains, with only a few ions located at the interface between PLF and COF316 in the initial snapshot (Figure 3h). Nevertheless, some Li^+^ ions in PLF tended to migrate toward COF316 in the snapshot taken after 20 ns, owing to the robust attraction by the high density of electron-withdrawing cyano groups on COF316. Furthermore, the plentiful ether-oxygen sites in both COF316 and the confined PLF chains can cooperatively establish a well-ordered and continuous ion transport channel along their interface, facilitating rapid and oriented Li^+^ migration along the layer-by-layer direction of COF316. In contrast, the simulations show that the Li^+^ ions can only be transported along the amorphous region of PLF chains in the pure PLF system, resulting in an anisotropic and sluggish ion migration (Figure S15). Moreover, the radial distribution function (RDF­(*r*)) exhibits that the dominant peak for Li–O in PLF appears at ∼2.0 Å (Figure S16a), which is similar to the RDF­(*r*) for COF316 (Figure S16b), indicating that the PLF chain and COF316 possess a similar affinity for Li^+^, enabling them to collaboratively serve as hopping sites for stable Li^+^ transport. As shown in Figure S17, a notable peak for Li–N in COF316 occurs at ∼2.2 Å, suggesting the ion–dipole interaction of −C≡N···Li^+^. The diffusion rate of Li^+^ in both electrolytes can be qualitatively assessed through mean-squared displacement (MSD) analysis. As shown in Figure 3i, the Li^+^ ion diffusion rate of PLF@COF316 was calculated to be 2.24 × 10^–8^ cm^2^ s^–1^, which is much higher than that of PLF (1.26 × 10^–8^ cm^2^ s^–1^), demonstrating a significantly enhanced Li^+^ diffusion kinetics in the PLF@COF316 electrolyte, aligning with above simulations and experiments.^28^

XPS measurement was carried out to further verify the interaction between COF316 and PLF. As shown in Figure 4a, apart from the −C≡N band, a typical peak belonging to −C≡N–Li can be found in the XPS N 1s orbital of PLF@COF316, demonstrating the ion–dipole interactions of −C≡N···Li^+^. The local chemical environments of COF316 interacting with PLF were further examined by ^7^Li solid-state NMR. As displayed in Figure 4b, the chemical shift of ^7^Li for PLF@COF316 (δ = −0.53 ppm) is significantly downfield shifted compared to that of pure PLF (δ = −1.18 ppm), indicating a decreased electron cloud density around the Li atom and looser coordination with the electron-donating oxygen in −SO_3_^–^, which further proves that the COF316 framework is crucial for dissociating Li^+^ from polyanions and facilitating rapid Li^+^ migration.^29^

**Figure 4:**
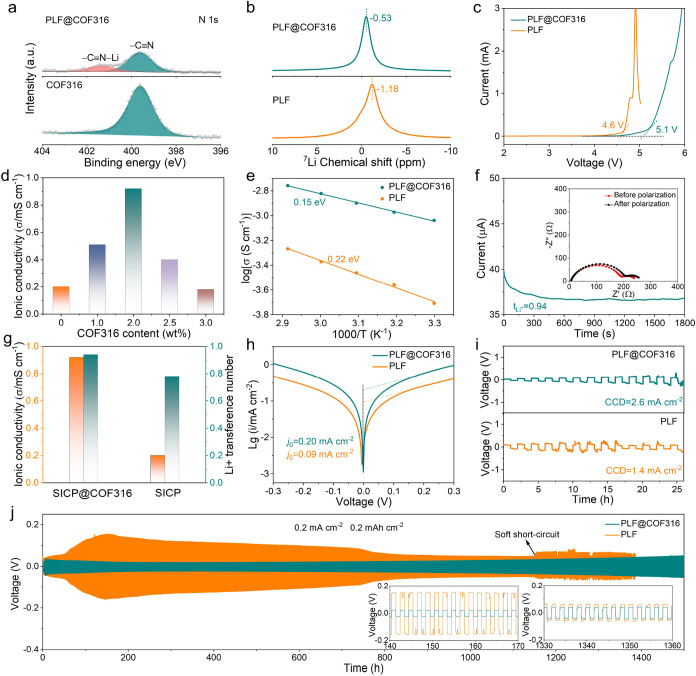
Li^+^ transport in PLF@COF316. (a) XPS N 1s spectra of PLF@COF316 and COF316. (b) ^7^Li solid NMR, (c) linear sweep voltammetry (LSV) curves, and (d) ionic conductivity of PLF@COF316 with different COF316 contents at 30 °C. (e) Arrhenius plots of PLF@COF316 and PLF. (f) Chronoamperometry polarization curve and the impedance spectra before and after polarization of the Li|PLF@COF316|Li symmetric cell. (g) Summarized σ_Li^+^_ and *t*_Li^+^_ of PLF@COF316 and PLF electrolytes. (h) Tafel plots, (i) critical current density (CCD) tests, and (j) voltage–time plots at 0.2 mA cm^–2^ and 0.2 mAh cm^–2^ of Li||Li symmetric cells with PLF@COF316 and PLF electrolytes.

Linear sweep voltammetry (LSV) measurements reveal that the electrochemical stability window increases from 4.6 to 5.1 V after the introduction of COF316, which is attributed to the highly oxidative-resistant cyano groups on the COF316 framework (Figure 4c).^30,31^ The temperature-dependent ionic conductivity was tested by electrochemical impedance spectroscopy (EIS) over the temperature range from 30 to 70 °C. We optimized the COF316 content to achieve the highest ionic conductivity in PLF@COF316 (Figures 4d and S18). COF316 greatly promotes the ionic conductivity of PLF. When the COF316 content is up to 2.0 wt %, the PLF@COF316 electrolyte achieves the highest ionic conductivity of 9.2 × 10^–4^ S cm^–1^ at 30 °C, which is 4.6 times that of PLF (2.0 × 10^–4^ S cm^–1^). However, excessive COF addition could result in aggregation and decrease the free volume of the polymer, thus hindering the ionic transport.^27^ As correspondingly depicted in Figure 4e, the activation energy (*E*_a_) of PLF@COF316 is calculated to be 0.15 eV, much lower than that of PLF (0.22 eV). Furthermore, the Li^+^ transference number was measured at RT to explore the selective ionic conduction of as-prepared SICPEs. As shown in Figures 4f and S19, PLF@COF316 displays a remarkable *t*_Li^+^_ up to 0.94, which is much higher than that of PLF (0.80). The improved *t*_Li^+^_ might be attributed to the anchoring effect of residual SSLi molecules by the cyano group on COF316. The σ_Li^+^_ and *t*_Li^+^_ of PLF@COF316 and PLF electrolytes are summarized in Figure 4g. As shown in Figure 4h, the Tafel curves reveal that the exchange current density (*j*_0_) of PLF@COF316 (0.20 mA cm^–2^) is much higher than that of PLF (0.09 mA cm^–2^), indicative of improved Li^+^ transport kinetics at the interface between the Li anode and the electrolyte.^32^ Furthermore, the Li|PLF@COF316|Li cell presents a critical current density (CCD) of 2.6 mA cm^–2^ (Figure 4i), which is significantly higher than that of PLF (1.4 mA cm^–2^). The enhanced CCD can be ascribed to more efficient Li^+^ transport and a robust electrode/electrolyte interface.

The electrochemical stability of the SICPEs with lithium metal anodes was assessed by a Li||Li symmetric cell at 30 °C. As displayed in Figure 4j, the Li|PLF@COF316|Li cell exhibits steady galvanostatic Li plating/stripping for 1500 h, with a small polarization voltage of ∼104 mV at a current density and capacity of 0.2 mA cm^–2^ and 0.2 mAh cm^–2^, respectively. In stark contrast, the Li|PLF|Li cell suffers fluctuating Li plating/stripping with large polarization voltage until a soft short circuit occurs after 1150 h, indicating an excellent interface stability toward lithium metal and even Li^+^ deposition with PLF@COF316. The significantly enhanced interface stability can be ascribed to the superb Li^+^ transport and robust solid electrolyte interphase (SEI) film formed on the Li anode surface. The EIS results depict that the interfacial resistance of the Li|PLF@COF316|Li symmetric cell is much lower than that of Li|PLF|Li both before and after cycling, implying better interfacial compatibility and stability between PLF@COF316 and lithium metal (Figure S20 and Table S3).^33^ As shown in Figure S21, compared with the smooth and uniform surface on the cycled Li anode using PLF@COF316, the Li anode with PLF presents a rough surface with numerous Li dendrites, leading to ongoing side reactions and cell failure.

### Li-Storage Performance of PLF@COF316 SICPE

NCM622||Li and LiNi_0.8_Co_0.1_Mn_0.1_O_2_ (NCM811)||Li quasi-solid-state cells were assembled to evaluate the practicality of our proposed SICPE and were tested at 30 °C. As illustrated in Figure 5a, the NCM622|PLF@COF316|Li cell delivers a remarkable reversible capacity of 139.4 mAh g^–1^ at 0.5 C after 1000 cycles under a cutoff voltage of 4.3 V, with an impressive capacity retention of 92.0% and a capacity decay of only 0.008% per cycle. In contrast, the capacity decreased to 46.3 mAh g^–1^ after 400 cycles for the NCM622|PLF|Li cell. Furthermore, the capacity–voltage curve of the NCM622|PLF@COF316|Li cell reveals its durable cycle over 1000 cycles without evident polarization (Figure S22). With a high loading of up to 8.8 mg cm^–2^, the NCM622|PLF@COF316|Li cell provides a stable cycle with a reversible capacity of 146.5 mAh g^–1^ after 200 cycles at 0.5 C, showcasing its excellent potential for practical application (Figure S23). Further, PLF@COF316 is paired with a high-nickel NCM811 cathode to assess its promise for high-specific-energy SSLMBs. As shown in Figure S24, the NCM811|PLF@COF316|Li cell can stably operate for 300 cycles with discharge capacity retaining 133.5 mAh g^–1^ at 0.5 C. By contrast, the capacity of the NCM811|PLF|Li cell rapidly decays to 57.4 mAh g^–1^ after 150 cycles. In addition, the NCM811|PLF@COF316|Li cell delivers specific capacities of 202.5, 201.0, 193.1, 184.0, and 170.1 mAh g^–1^ at stepwise current densities of 0.1, 0.2, 0.5, 1, and 2 C, respectively, and the capacity is reversible with the current density going back to 0.1 C (Figure 5b). In contrast, the NCM811|PLF|Li cell displays inferior discharge capacity at different current densities, with *a* capacity of only 98.1 mAh g^–1^ at 2.0 C. Correspondingly, the charge/discharge plateaus of NCM811|PLF@COF316|Li always remain more stable with smaller polarization compared to NCM811|PLF|Li at different current densities, indicating a superior fast-charging capability of PLF@COF316 (Figure S25). The EIS curves display that the charge transfer resistance of NCM811|PLF@COF316|Li (52.2 Ω) is significantly lower than that of NCM811|PLF|Li (163.4 Ω) after rate cycling, indicating a fast Li^+^ transport and excellent interface compatibility of PLF@COF316 (Figure S26 and Table S4).^34^

**Figure 5:**
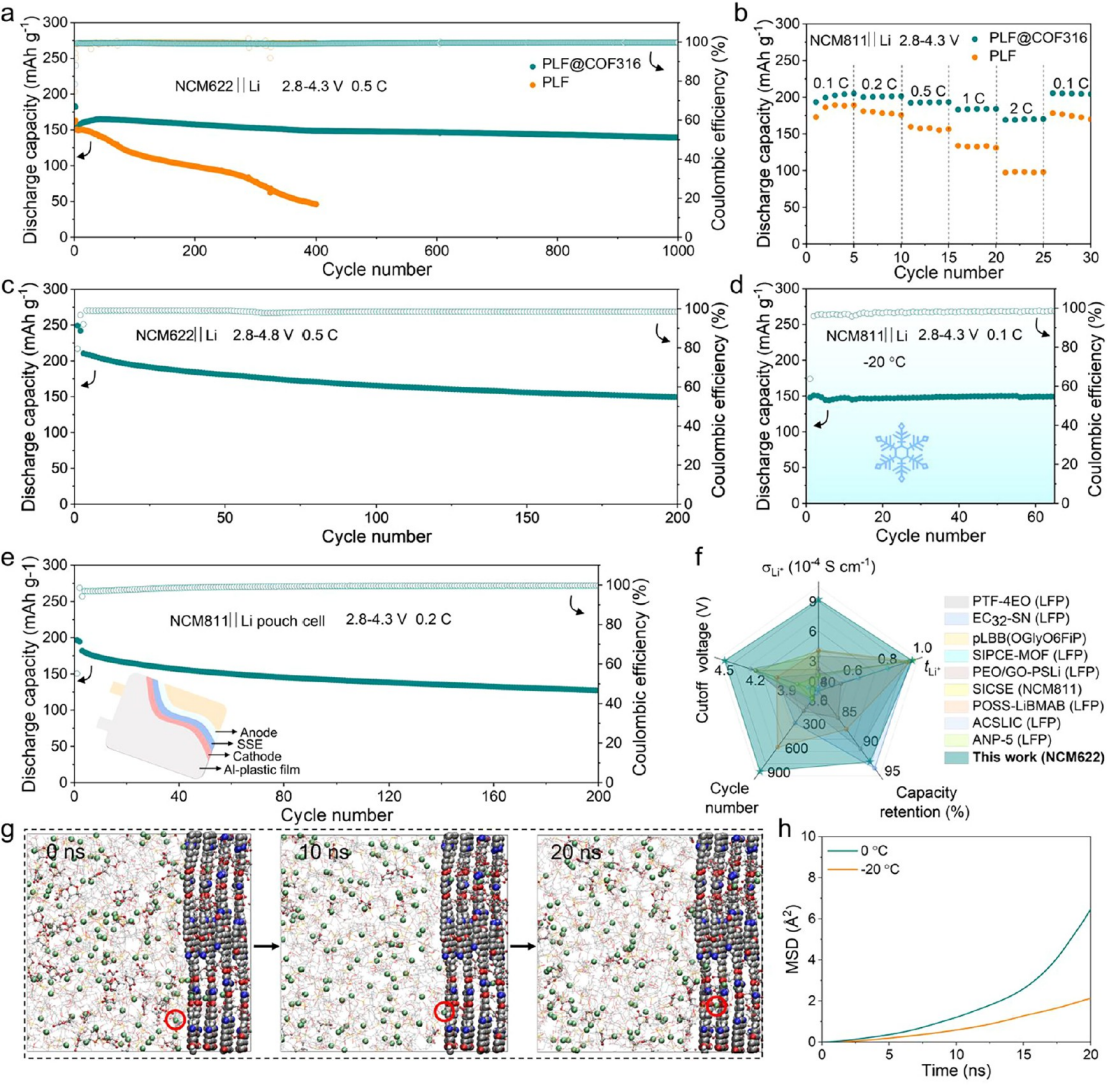
Li-storage performance of PLF@COF316. (a) Cycling performance of NCM622||Li cells with PLF@COF316 and PLF electrolytes at 0.5 C with a cutoff voltage of 4.3 V. (b) Rate performance of NCM811||Li cells with PLF@COF316 and PLF electrolytes at current densities from 0.1 to 2 C. (c) Cycling performance of the NCM622|PLF@COF316|Li cell at 0.5 C with a cutoff voltage of 4.8 V. (d) Cycling performance of the NCM811|PLF@COF316|Li cell at −20 °C and 0.1 C. (e) Cycling performance of the NCM811|PLF@COF316|Li pouch cell at 0.2 C. (f) Comparison of σ_Li^+^_, *t*_Li^+^_, cutoff voltage, and cycling performance with reported SICPEs. (The capacity retentions only select from the cycling performance over 200 loops.) (g) Molecular dynamics simulation snapshots of the Li^+^ migration in the PLF@COF316 system at 0 °C. (h) The MSD of Li^+^ for PLF@COF316 at 0 and −20 °C.

Remarkably, even under a cutoff voltage of 4.8 V, the NCM622|PLF@COF316|Li cell continues to exhibit a high reversible capacity of 149.5 mAh g^–1^ at 0.5 C after 200 cycles with a capacity retention of 71.0% (Figure 5c), demonstrating excellent potential for high-specific-energy SSLMBs. The NCM811|PLF@COF316|Li cell was cycled at −20 °C to explore the Li^+^ migration and Li-storage performance of PLF@COF316 under extreme conditions. As shown in Figure 5d, the NCM811|PLF@COF316|Li cell delivers a stable loop with almost no capacity degradation at 0.1 C after 65 cycles, indicating a remarkable Li-storage capability at low temperatures.

Based on the superior coin cell performance, the NCM811|PLF@COF316|Li pouch cell was assembled to further investigate the practical feasibility of PLF@COF316. As shown in Figure 5e, the NCM811|PLF@COF316|Li pouch cell delivers a reversible specific capacity of 127.4 mAh g^–1^ (corresponding to an energy density of 282.0 Wh kg^–1^ excluding the Al-plastic film) over 200 cycles at 0.2 C with an exceptional capacity retention of 70.0%, indicative of an excellent long-term cycling stability for successful implementation in SSLMBs. The overview of the NCM811|PLF@COF316|Li pouch cell is shown in Table S5. Additionally, the corresponding charge–discharge curves reveal the favorable capacity reversibility of the NCM811|PLF@COF316|Li pouch cell (Figure S27). In terms of safety, the NCM811|PLF@COF316|Li pouch cell is also capable of enduring rigorous destructive tests (Figure S28), still powering a light-emitting diode (LED) device to even suffer harsh cutting and folding, indicative of excellent practicality and short circuit prevention. The PLF@COF316 electrolyte demonstrates an exceptional advantage in comparison to previously reported SICPEs in terms of σ_Li^+^_, *t*_Li^+^_, cutoff voltage, and cycling performance, positioning it among the top-performing SICPEs (Figure 5f and Table S6).

Given the excellent cycling stability of the NCM811|PLF@COF316|Li cell at −20 °C, the MD simulations were employed to reveal its ion transport behavior at low temperatures. The snapshots depict that the strong attraction exerted by the electron-withdrawing cyano groups in COF316 allows Li^+^ ions in PLF to migrate toward the COF316 framework, even at low temperatures of 0 and −20 °C (Figures 5g and S29). MSD analyses indicate that the Li^+^ ion diffusion rates of PLF@COF316 are 4.02 × 10^–9^ and 1.78 × 10^–9^ cm^2^ s^–1^ at 0 and −20 °C, respectively (Figure 5h). They are lower than the Li^+^ ion diffusion rate at room temperature (2.24 × 10^–8^ cm^2^ s^–1^), showcasing that ionic diffusion slows down with decreasing temperature, which is attributed to the decelerated creep of polymer segments at low temperatures. The temperature-dependent EIS from 20 to −20 °C was conducted to assess the ionic conductivity at low temperatures (Figure S30a,b). PLF@COF316 exhibits an ionic conductivity of 1.8 × 10^–4^ S cm^–1^ at −20 °C (Figure S30c), which is near the ionic conductivity of PLF at room temperature and 10 times that of PLF at −20 °C (1.8 × 10^–5^ S cm^–1^). The *E*_a_ of PLF@COF316 for Li^+^ transport is calculated to be 0.22 eV at −20 °C (Figure S30d), which is significantly lower than that of PLF (0.27 eV). The excellent ionic conductivity of PLF@COF316 at −20 °C guarantees the stable cycling of the NCM811||Li cell at low temperatures.

### Structural Stability and Interfacial Evolution Analysis

The electrode structure and interfacial evolution of both the cathode electrolyte interphase (CEI) and SEI were investigated to reveal the mechanisms of enhanced electrochemical performance. The surface phase transition from a layered (Rm) structure to a rock-salt (Fmm) NiO-like configuration is recognized to cause severe structural degradation of the NCM cathode upon cycling. Compared to the ordered layered structure, the rock-salt phase is neither electrochemically active nor ionic conductive, leading to severe capacity decline and sluggish Li^+^ kinetics.^35^ To clarify the NCM811 deterioration, HAADF-STEM was applied to track the lattice phase change of NCM811 with different electrolytes after cycling. As shown in Figure 6a, two distinct regions were detected in NCM811 with the PLF electrolyte. Aside from the original layered structure within the bulk (region I), a severe phase transition to the NiO rock-salt phase was observed on the NCM811 surface (region II). The rock-salt phase results from the reduction of high-valence Ni ions with the electrolyte at high voltage, followed by the mixed arrangement of Li^+^/Ni^2+^ during the lithium intercalation/delithiation process.^36^ NCM cathodes under a highly charged state exhibit thermodynamic instability due to the unstable high valence state of the Ni cation. Ni^3+^ tends to be reduced to Ni^2+^ owing to the unpaired electron spin on the *e*_g_ orbital, while highly reactive Ni^4+^ suffers a rapid reaction with the electrolyte and then reduces to Ni^2+^ with a large loss of Li and oxygen. Due to the similar ionic radii of Li^+^ (0.76 Å) and Ni^2+^ (0.69 Å), Ni^2+^ ions tend to intermix with Li^+^ at high Ni content, resulting in poor capacity retention and Li diffusivity owing to the kinetic hindrance.^37^ In contrast, the bulk and surface of NCM811 with PLF@COF316 almost retained the original layered structure and were proved by fast Fourier transform (FFT) patterns, indicating that the COF316-derived CEI could avoid the generation of the resistive rock-salt phase, thereby maintaining the structural stability to mitigate the capacity decline under high voltage.^38^ TEM was conducted to further investigate the morphology of CEI. As shown in Figure S31a, NCM811 cycled in PLF exhibits an irregular and loosened CEI layer with a thickness of ∼12 nm. In contrast, a smooth and homogeneous CEI with a thickness of ∼5 nm is grown on the surface of NCM811 when cycling with the PLF@COF316 electrolyte (Figure S31b), showcasing an effective inhibition of interfacial side reactions.

**Figure 6:**
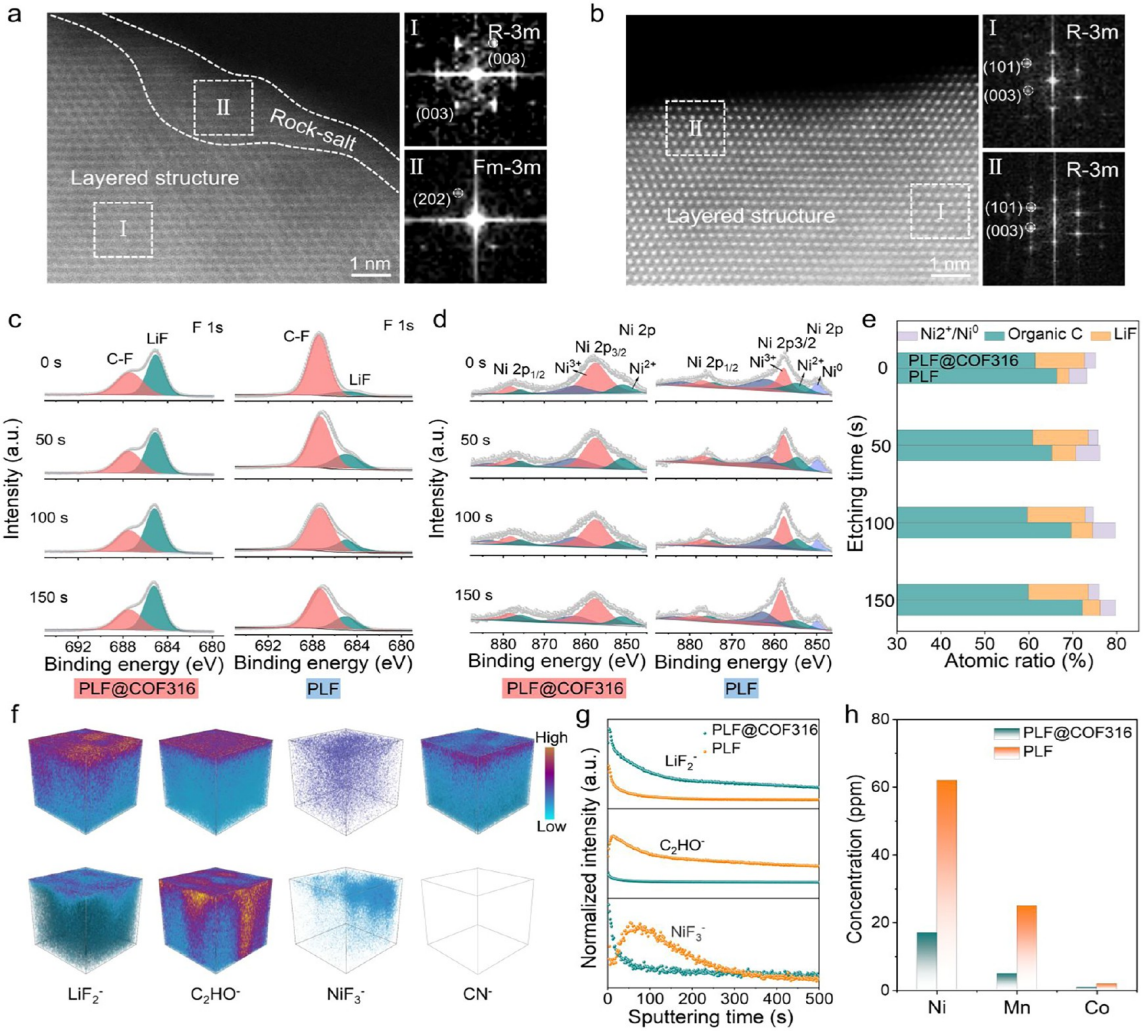
Characterization of the CEI. STEM images and corresponding FFT patterns for the NCM811 particles cycled in the (a) NCM811|PLF|Li and (b) NCM811|PLF@COF316|Li cells. XPS depth profiles of (c) F 1s and (d) Ni 2p of cycled NCM811 cathodes in the NCM811|PLF@COF316|Li and NCM811|PLF|Li cells. (e) Comparison of LiF, organic C, and reduced Ni content distribution in the cycled NCM811 cathodes. (The reduced Ni content is magnified 10 times for clear display in the figure.) (f) Time-of-flight secondary-ion mass spectrometry (TOF-SIMS) three-dimensional (3D) mappings in the formed CEI by PLF@COF316 (up) and PLF (bottom) electrolytes. (g) The corresponding TOF-SIMS depth profiles of various elemental segments. (h) TM dissolution measured by inductively coupled plasma mass spectrometry (ICP-MS) after 100 cycles.

XPS depth profiling was conducted to analyze the interface electrochemical environment of cycled NCM811 cathodes, and the fitting results are summarized in Table S7. As depicted in Figure S32, the C 1s spectra for both electrolytes show typical peaks corresponding to C–C/C–H, C–O, C=O, and −CO_3_. The organic C atomic concentration of the CEI with PLF@COF316 (60.0% after etching 150 s) is less than that with PLF (72.3%), implying fewer organic components resulting from electrolyte decomposition (Figure 6e and Tables S7 and S8). Organic-rich CEI is readily oxidized under high voltage and cannot withstand the large volume change of the cathode, leading to fracture upon cycling and continuous parasitic reactions between the cathode and electrolytes. Furthermore, the CEI generated from PLF@COF316 displays a higher intensity of LiF than that of PLF (Figure 6c), with an inner content exceeding 13.6%, compared to only 4.1% for PLF. LiF is widely recognized as a crucial component of CEI because of its high interface energy and excellent electronic insulation, serving a vital role in inhibiting electron tunneling and improving CEI stability. Impressively, the reduced Ni ions (Ni^2+^ and Ni^0^) concentration for the PLF@COF316 electrolyte is lower than that for PLF at various depths (Figure 6d,e), further confirming the restricted formation of the NiO rock-salt phase.^39^ The typical −C≡N and −C≡N–TM peaks were detected in N 1s spectra using PLF@COF316, proving that the −C≡N group participates in the formation of CEI and bonds with TM ions to inhibit their catalytic reactivity toward the PLF chain (Figure S33).^16,30^

Time-of-flight secondary-ion mass spectrometry (TOF-SIMS) was performed to further explore the surface compositions and depth distribution of CEI. The three-dimensional (3D) reconstruction images and 2D mappings illustrate the distribution of organic and inorganic components in CEI, with the LiF_2_^–^ fragment denoting the inorganic component of LiF, while the C_2_HO^–^ fragment represents the organic lithium compounds (Figures 6f, S34, and S35). The CEI formed in PLF@COF316 bears a much higher and more uniform signal intensity of LiF_2_^–^ and a lower signal intensity of C_2_HO^–^ in comparison to that of PLF, illustrating an effective inhibition of interface side reactions and electrolyte decomposition. Moreover, the signal intensity of NiF_3_^–^ is less in the CEI obtained from PLF@COF316 than that from PLF, and a uniform signal of CN^–^ derived from COF316 is detected on the surface of NCM811, indicating that the −C≡N group in COF316 participates in the formation of CEI and effectively restrain the TM dissolution. The depth profile shown in Figure 6g further validates the distribution of typical LiF_2_^–^, C_2_HO^–^, and NiF_3_^–^ fragments, aligning with the 3D views and XPS analysis. Inductively coupled plasma mass spectrometry (ICP-MS) measurements were carried out to quantify the transition metal dissolution. As shown in Figure 6h, the contents of Ni, Co, and Mn elements dissolved into the Li anode in the NCM811|PLF@COF316|Li cell are notably lower than those in the NCM811|PLF|Li cell after cycling, implying the restrained TM dissolution and cathode degradation.^16^

To further explore the factors contributing to the outstanding Li plating/stripping reversibility, cryo-TEM was used to analyze the structure and composition of the SEI at the atomic level. Li metal was deposited onto a Cu grid with a capacity of 0.25 mAh cm^–2^ at a current density of 0.5 mA cm^–2^ for cryo-TEM measurement. As shown in Figure 7a, a continuous and smooth SEI is grown on the surface of the deposited Li metal using the PLF@COF316 electrolyte. The SEI presents a classical mosaic structure featuring an amorphous matrix with embedded inorganic nanocrystals, including LiF, Li_2_O, Li_3_N, and Li_2_CO_3_ species, which are conducive to promoting Li^+^ transport across SEI and suppress uncontrolled dendrite growth (Figure 7b).^40^ The EDS mapping shows the uniform distribution of C, N, F, and O elements throughout the SEI (Figures 7c and S36). Specifically, the main crystalline lattices with calibrated interplanar spacings of 2.01 Å (Figure 7d), 2.78 Å (Figure 7e), and 2.66 Å (Figure 7f) are captured from the high-resolution images and the corresponding FFT patterns, aligned with the LiF(200), Li_3_N­(101), and Li_2_O­(111) planes, respectively.

**Figure 7:**
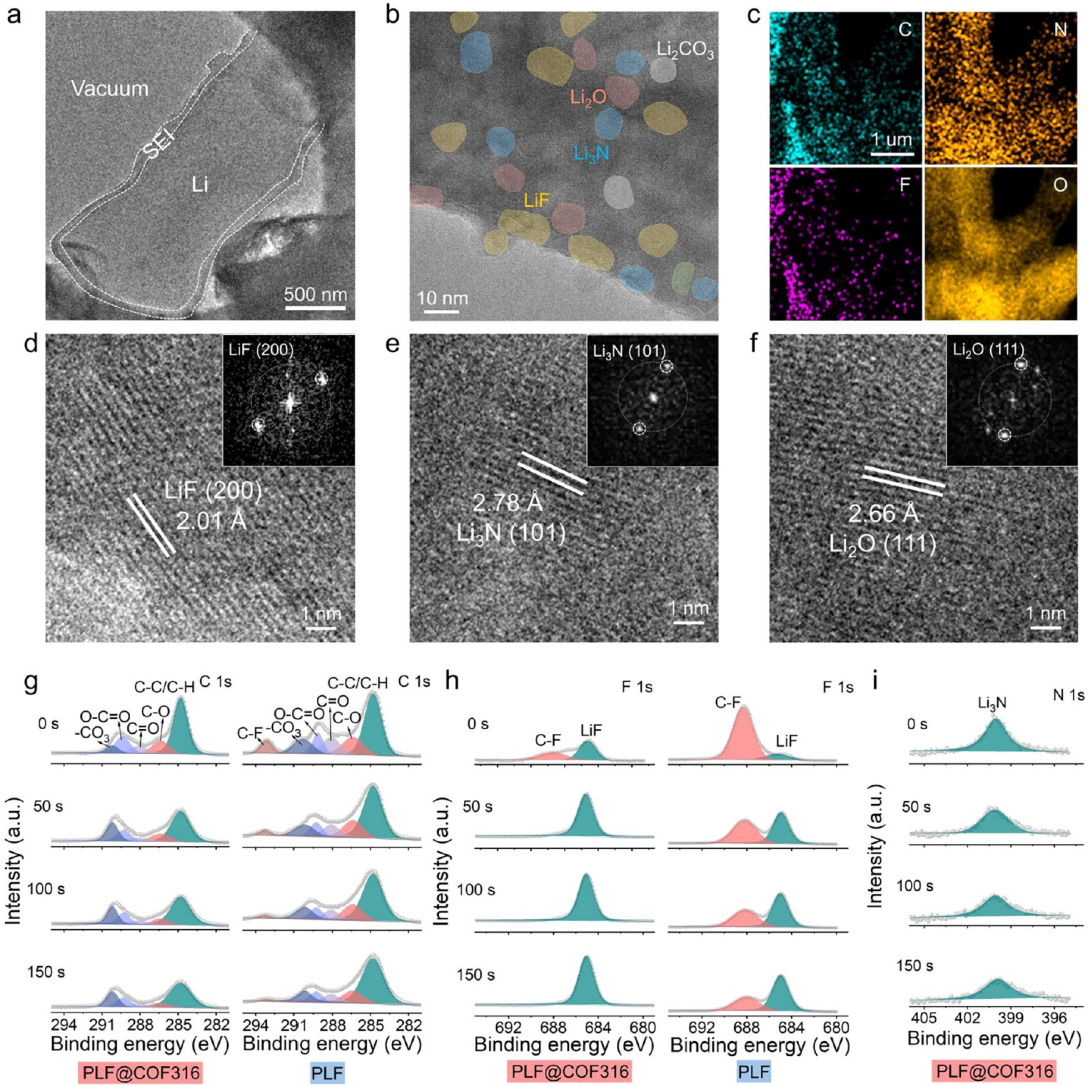
Characterization of the SEI. Cryo-TEM images of SEI on the Li anode surface formed by the PLF@COF316 electrolyte at (a) low magnification and (b) high resolution. (c) EDS mappings of the surface of the deposited Li. The enlarged high-resolution TEM images and corresponding FFT patterns of (d) LiF, (e) Li_3_N, and (f) Li_2_O. XPS depth profiles of (g) C 1s, (h) F 1s, and (i) N 1s of Li anodes cycled in NCM811|PLF@COF316|Li and NCM811|PLF|Li cells.

XPS depth profiling was employed to further probe the composition of SEI, and the fitting results are summarized in Table S9. Compared with PLF, the generated SEI using PLF@COF316 bears a much lower organic C species, revealing that the decomposition of the electrolyte on the metallic Li surface is effectively inhibited (Figure 7g). Furthermore, the Li anode cycled with PLF@COF316 exhibits a higher concentration of LiF than that of PLF in the F 1s spectra, which is conducive to restricting lithium dendrite growth because of its high interface energy and superior electronic insulation (Figure 7h).^40^ As shown in Figure 7i, Li_3_N species is also formed in the SEI using PLF@COF316, which is attributed to the reduction of the −CN group and enables enhanced Li^+^ transport across SEI owing to its high ion conductivity.^41^ On the contrary, there is no obvious signal of N species in the N 1s spectra with PLF (Figure S37). Moreover, the intensities of inorganic species such as LiF and Li_2_O using PLF@COF316 obviously increase with sputtering depth (Figures 7h and S38), while the intensities of C peaks decrease rapidly, demonstrating a dual-layer SEI with an amorphous organic outer layer and an inorganic-rich inner layer. The organic outer layer can improve the mechanical strength, while the inner inorganic-rich phases facilitate uniform Li^+^ deposition and rapid Li^+^ migration, thus allowing for dendrite-free lithium deposition and robust interfacial stability.^24^

COF316-COOH with an electron-withdrawing carboxyl group was synthesized to compare with cyano-containing COF316 in terms of cell performance for high-voltage SSLMBs (Figures S39–S42). The ionic conductivity of PLF@COF316-COOH is 7.9 × 10^–4^ S cm^–1^ (Figure S43), slightly lower than that of PLF@COF316 but ∼4 times that of pure PLF, indicating that the incorporation of COFs with electron-withdrawing groups can effectively promote polyanion dissociation and enhance ionic conductivity of SICPEs. However, the specific capacity of the NCM622|COF316-COOH|Li cell decreased to 122.2 mAh g^–1^ at 0.5 C after 200 cycles (Figure S44), significantly lower than that of NCM622|PLF@316|Li (157.9 mAh g^–1^), demonstrating that a cyano-containing COF not only enhances the ionic conductivity but also is more conducive to the stable cycle of NCM cathodes at high voltage. To sum up, the results discussed above demonstrate that the fusion of a cyano-containing COF with SICPEs can simultaneously upgrade their ionic conductivity and interface stability, ultimately enhancing the Li-storage performance in high-voltage SSLMBs.

## Conclusions

In summary, we developed a novel strategy of incorporating a cyano-containing COF with an SICPE to upgrade its Li^+^ migration and interfacial stability toward high-voltage SSLMBs. Experiments and theoretical simulations prove that the ion–dipole interaction of −C≡N···Li^+^ promotes Li^+^ dissociation from the polyanions, resulting in more free-moving Li^+^. The confinement of PLF chains within/around COF316 pores facilitates the rapid Li^+^ migration via the ether-oxygen hopping sites between PLF and COF316. Furthermore, the HFBA segment and cyano group on the COF316 framework contribute to the formation of F, N-rich electrolyte/electrode interfacial films, inhibiting the TM dissolution and promoting uniform Li deposition. As a result, the PLF@COF316 electrolyte exhibits a significantly enhanced Li^+^ transport with superior ionic conductivity (9.2 × 10^–4^ S cm^–1^) and a high Li^+^ transference number (0.94). The assembled NCM622||Li cell exhibits a remarkable capacity retention of 92.0% over 1000 cycles, and the cell can also cycle stably when paired with a 4.8 V NCM622 cathode, positioning them among the top-performing SICPEs. The innovative strategy demonstrates great promise for the development of long-cycling and high-voltage SSLMBs.

## Supplementary Material



## Notes

Experimental section; computer calculation; Figures S1–S44; and Tables S1–S9 (DOCX)
