# Supplementary material for: Upgrading Ion Migration and Interface Chemistry via a Cyano-Containing COF in a Single-Ion Conductive Polymer toward High-Voltage Lithium–Metal Batteries
Source: J Am Chem Soc. 2025 Jul 11;147(29):25896–909. doi: 10.1021/jacs.5c08267 (PMC12291442; doi:10.1021/jacs.5c08267)
Supplement: Supplementary file 1 [file ja5c08267_si_001.docx]

**Supporting Information**

**Upgrading Ion Migration and Interface Chemistry via a Cyano-****Containing COF in a Single-Ion Conductive Polymer toward High-Voltage Lithium-Metal Batteries**

Xiaosa Xu^a^, Junjie Chen^a^, Jin Li^a^, Jiadong Shen^a^, Pengzhu Lin^a^, Zhenyu Wang^a^, Zixiao Guo^a^, Jing Sun* ^a^, Baoling Huang* ^a^, Tianshou Zhao* ^a,b^

^a^ Department of Mechanical and Aerospace Engineering, The Hong Kong University of Science and Technology, Clear Water Bay, Kowloon 999077, Hong Kong SAR, China

^b^ Department of Mechanical and Energy Engineering, Southern University of Science and Technology, Shenzhen 518055, China

E-mail: jsunav@connect.ust.hk (J. Sun); mebhuang@ust.hk (B.L. Huang); zhaots@sustech.edu.cn (T.S. Zhao).

**Experimental Section**

**Materials.** HHTP, TFTPN, triethylamine, 1,4-dioxane, HFBA, PEGDA, 2-hydroxy-2-methylpropiophenone, and Nmethyl-2-pyrrolidone (NMP) were purchased from Aladdin. SSLi was purchased from EXTENSION. Propylene carbonate (PC), floroethylene carbonate (FEC), 1-Ethyl-3-methylimidazolium bis(trifluoromethylsulfonyl)imide) (EMImNTF_2_), and lithium foil were purchased from DoDoChem. Poly(vinylidene fluoride) (PVDF), N-methyl-2-pyrrolidone (NMP), and Super P were purchased from Shenzhen Kejing. NCM811 and NCM622 were purchased from Guangdong Canrd. All reagents were used as received without further purification.

**Synthesis of COF316.** The synthesis approach of COF316 follows the previously reported literature. HHTP (30.0 mg, 0.0928 mmol), TFPN (27.6 mg, 0.138 mmol), triethylamine (78.0 μL), and 1,4-dioxane (2.0 mL) were charged to a dried Pyrex tube. After sonication for 10 min, the tube was flash-frozen in a liquid N_2_ bath (77 K). After three freeze-pump-thaw cycles, the system was sealed off and heated at 120 °C for 72 h. The precipitate was achieved by filtration and washed with DMF (15 mL × 5). Then, the wet powder was transferred to a Soxhlet extractor and washed with THF for 24 h and acetone for 24 h. Finally, the product was evacuated at 120 °C overnight to yield the activated sample. The yield of collected COF316 is ~83 % (38.6 mg).

**Synthesis of COF316-COOH.** The synthesis of COF316-COOH follows the previously reported literature. The mixture of COF316 powder (200.0 mg) and 20% NaOH solution (H_2_O:ethanol = 1:1, 50.0 ml) was heated to 120 °C and left to stir at this temperature under reflux for 72 h. The powder was filtrated and refluxed in water for 2 h, then refluxed in 1 M HCl for another 2 h. The solid was collected by vacuum filtration, then washed with water and THF. Then, the wet powder was transferred to a Soxhlet extractor and washed with THF for 24 h. The solvent was then exchanged with anhydrous acetone and evaporated under vacuum at 80 °C, acquiring COF316-COOH as a brown powder. The yield of collected COF316-COOH is ~95 % (190.2 mg).

**Synthesis of PLF@COF316 Film.** To prepare PLF@COF316 film, monomers of SSLi, PEGDA, HFBA, and FEC with a mass ratio of 5:4:1:1 were dissolved in DMSO. Then, COF316 (2wt% relative to the total mass of monomers) was added into the as-acquired mixture and ultrasound for 30 min to ensure a uniform dispersion. Followed by adding 2-hydroxy-2-methylpropiophenone (1wt%) as initiator, the as-prepared precursor solution was exposed to a 365 nm UV lamp for 30 min until an even polymer film was obtained, which was then dried in a glove box for 24 h to obtain PLF@COF316 film. Afterward, the as-acquired PLF@COF316 film was cut into 18 mm disks to assemble cells. The preparation processes of PLF and PLF@COF316-COOH films are the same as above but without COF316 and with COF316-COOH, respectively. The degree of polymerization is calculated to be 93.1%. The yields of collected PLF@COF316, PLF, and PLF@COF316-COOH are ~96 %, ~94%, and ~95 %, respectively.

**Preparation of NCM811/NCM622 Cathodes.** NCM811/NCM622 powders (80 wt%), Super P (10 wt%), and PVDF (10 wt%) as a binder dissolved in NMP were blended into a slurry and coated onto the carbon-coated Al foil. Afterward, the coated foil was dried in a vacuum oven at 80 °C for 12 h and then punched into disks with a diameter of 12 mm. The mass loading of NCM811/NCM622 on each disk is ∼2.5 mg cm^-2^.

**Battery Assembly and Test**. To assemble the Li||Li symmetric cell, the SICPE film was sandwiched between two Li foils. For the preparation of NCM622||Li and NCM811||Li full cells, the SICPE film was sandwiched between an NCM811/NCM622 cathode and a Li foil. To decrease the interfacial resistance, a minute volume of EMImNTF_2_/FEC (1:1) electrolyte (5 μL) was carefully added at the interface between the SICPE and the electrode. All coin cells used were CR2032 type, and all the operations were carried out in the Ar-filled glove box (H_2_O < 0.01 ppm, O_2_ < 0.01 ppm). Galvanostatic charge and discharge tests were conducted on the Neware battery testing system at 30 °C and -20 °C. EIS measurements were carried out over a frequency range of 7.0 MHz to 0.1 Hz on Bio-logic VMP3 potentiostat/galvanostat.

**Electrochemical Measurements.** Ionic conductivity was measured using Biologic VMP3 potentiostat/galvanostat by alternating current (AC) impedance spectroscopy over a temperature range of -20 °C to 70 °C with two stainless steels (SS) as electrodes in a CR2032 coin cell. The spectra were recorded within a frequency range of 7.0 MHz to 0.1 Hz. The ionic conductivity ($\sigma$) was calculated according to **Equation 1**:

$$\sigma=\frac{l}{RA}$$

Here, *σ* is ionic conductivity (S cm^-1^), *R* denotes the ionic resistivity (Ω), *l* is the thickness of the electrolyte membrane, and *A* refers to the contact area between the electrolyte and SS electrode (cm^2^).

The activation energy (*E_a_*) for Li^+^ transport was determined according to **Equation 2**:

$\sigma=Ae^{\frac{-E_{a}}{RT}}$

Where *A* represents the pre-exponential factor, *E_a_* is the activation energy for Li^+^ transport, *R* is the ideal gas constant, and *T* is the absolute temperature.

The electrochemical stability window was assessed using linear scanning voltammetry (LSV) conducted in a configuration of SS||Li cell (scan rate: 1 mV s^-1^) over a voltage range of 0-6 V (*vs*. Li^+^/Li).

Li^+^ transference number ($t_{{Li}^{+}}$) was evaluated using a Li||Li symmetric cell by combining an AC impedance measurement with a direct current (DC) potentiostatic polarization. EIS was measured over a frequency range of 7.0 MHz to 0.1 Hz before and after the DC polarization (with a voltage amplitude of 10 mV). The *t_Li+_* was calculated using the modified Bruce-Vincent-Evans (BVE) **Equation 3**:

$$t_{{Li}^{+}}=\frac{I_{s}R_{0}(\Delta V-I_{0}R_{0})}{I_{0}R_{s}(\Delta V-I_{s}R_{s})}$$

Where $t_{{Li}^{+}}$ is the Li^+^ transference number, Δ*V* is the polarization voltage of 10 mV, *I_0_* and *I_s_* are the initial and steady-state currents, and *R_0_* and *R_s_* are the resistances before and after polarization, respectively.

**Characterization.** The morphology, structure, and elemental mapping analysis were monitored by scanning electron microscope (SEM, JEOL-6700 F) and transmission electron microscopy (TEM, JEOL JEM-ARM200F). The compositions and chemical states of the samples were investigated by X-ray photoelectron spectroscopy (AXIS Supra, Kratos). Fourier-transform infrared spectroscopy (FT-IR) data were obtained using a PerkinElmer Spectrum Two spectrometer. Solid-state Nuclear Magnetic Resonance (NMR) was tested using an Agilent 600M spectrometer. N_2_ adsorption-desorption measurement was conducted using a surface area and pore size distribution analyzer (Micromeritics ASAP 2020). Powder X-ray diffraction (PXRD) pattern was collected by a Bruker D2 Phaser XE-T X-ray diffractometer with Cu Kα radiation source (λ = 1.5406 Å). Time-of-flight secondary ion mass spectrometer tests were carried out using a TOF-SIMS 5 iontof (PHI NanoTOFII). The differential scanning calorimetry (DSC) curve was collected on a DSC Q2000 (TA Instruments) at a heating rate of 10 °C min^-1^. The stress-strain measurement was performed on a universal stretching machine (CMT6103). ICP-MS measurement was performed on a PerkinElmer ICP 2100 analyzer.

For cryo-TEM measurement, a bare grid was positioned on Cu foil and assembled as a Li||Cu cell in an Ar-filled glovebox. The cell was discharged at a current density of 0.5 mA cm^-2^ for 0.5 h. After the grid was rinsed with DEC and dried, it was carefully transferred into the cryo-TEM holder in the glovebox. Employing a sealed container, the cryo-TEM holder was quickly inserted into the cassette. Then liquid nitrogen was poured into the holder until the sample temperature decreased below -170 °C. The cryo-TEM measurement was performed on a JEOL JEM-2100Plus microscope.

**Computational Methods and Models.** In the MD simulations, molecular models of PLF and FEC were constructed using Materials Studio. Parameters of PLF polymer were derived from the CL&P parameters, while Li^+^, COF316, and FEC were simulated using the Amber03 forcefield and RESP charge model. Topology files for these various components were generated using the AuToFF web server and Sobtop.

All-atom MD simulations were performed using the GROMACS software package, version 2021.5. In the simulation, we first constructed a system consisting of 6 layers of COF316 as the base. The initial simulation box size was 6 nm × 6 nm × 10 nm, and system 1 was constructed by randomly filling 100 PLF and 150 FEC molecules above the COF316. Meanwhile, we also constructed system 2 without COF, which contains 100 PLF and 150 FEC molecules. Both systems were energy minimized to achieve initial relaxation and then annealed over 10 ns from 600 to 298.15 K, 273.15 K, and 253.15 K with a time step of 1 ps to achieve equilibrium, respectively. A velocity-scaling thermostat with a relaxation time of 1 ps was used in the NPT ensemble to maintain the temperature at 298.15 K, 273.15 K, and 253.15 K. A Berendsen's barostat with a semi-isothermal compressibility constant of 4.5×10^-5^ along the z-axis was utilized to adjust the pressure at 1 Pa to simulate 10 ns, ensuring full compression of the box. Then A constant electric field of 4V/nm is applied in the X direction in the NVT ensemble, with periodic boundary conditions are applied in all dimensions. The Particle mesh Ewald (PME) method with a cutoff distance of 0.12 nm is used to deal with the electrostatic interactions and van der Waals forces. Visualization of the simulation box was facilitated by the Visual Molecular Dynamics (VMD) program.

To simulate the interaction between Li^+^ and COF316, we expanded the COF316 into a nanosheet with eight pores, superimposed three layers, and placed the COF sheet at the center of the simulation box. The COF layer was surrounded by 100 randomly distributed PLF and 150 FEC molecules. After performing energy minimization, we conducted molecular dynamics simulations for 20 ns in an NVT set, during which the COF was frozen. After the simulation, ions and molecules were fully adsorbed on the COF lamella, with other conditions were consistent with the previous system.

In the Density Functional Theory (DFT) calculations, the Perdew-Burke-Ernzerhof (PBE) formulation within the generalized gradient approximation (GGA) was employed for the exchange-correlation interactions. For the solid-state calculations, the cutoff energy of the plane-wave basis is set at 520 eV and the Brillouin zone integration was performed on a 1×1×4 k-points grid. For the molecule calculations, the def-TZVP basis set was applied. The electron density and electrostatic potential obtained from DFT calculations were further analyzed using Multiwfn to generate the distribution of the electrostatic potential on the 0.01 a.u. isosurface of the electron density.


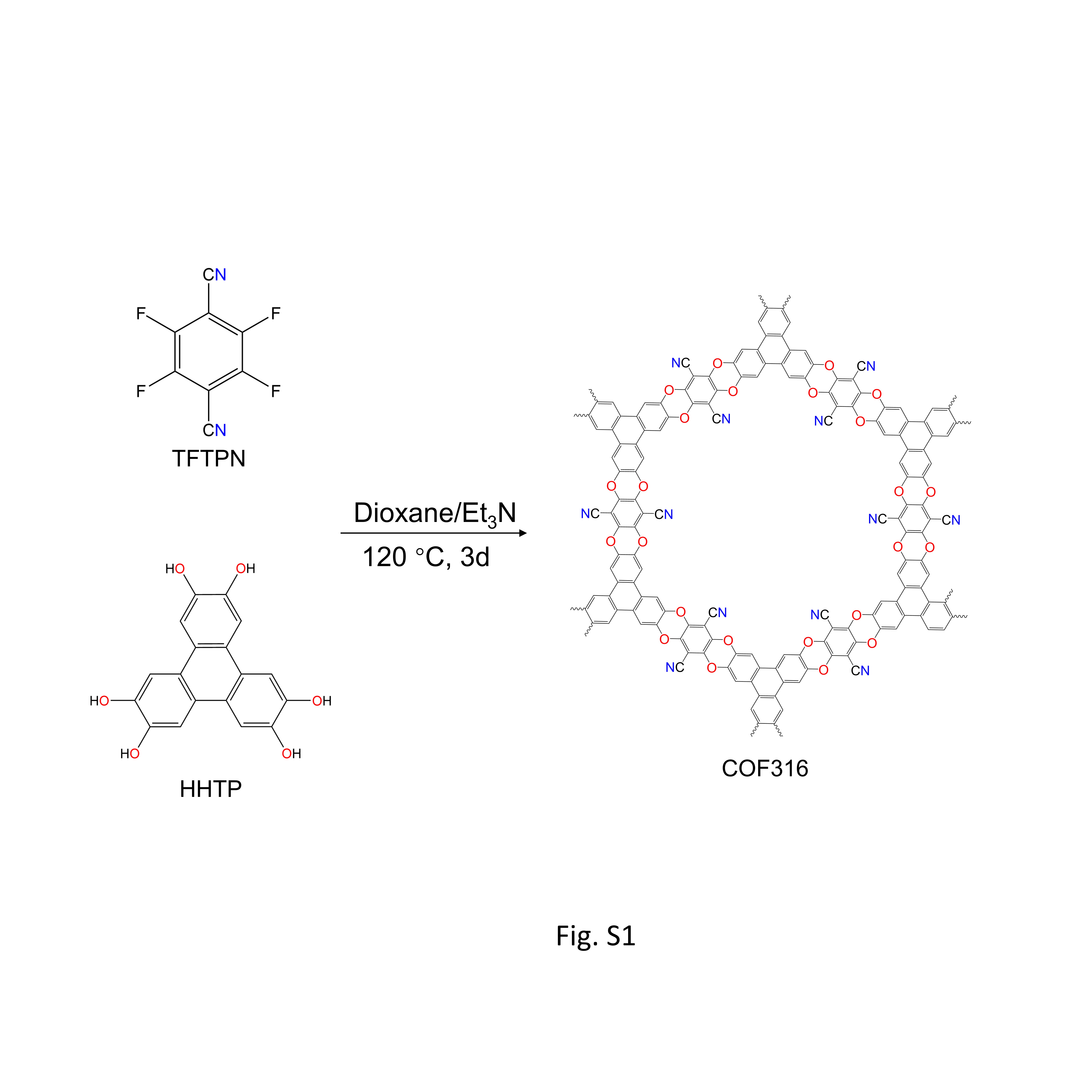


**Figure S1.** The synthesis of COF316.


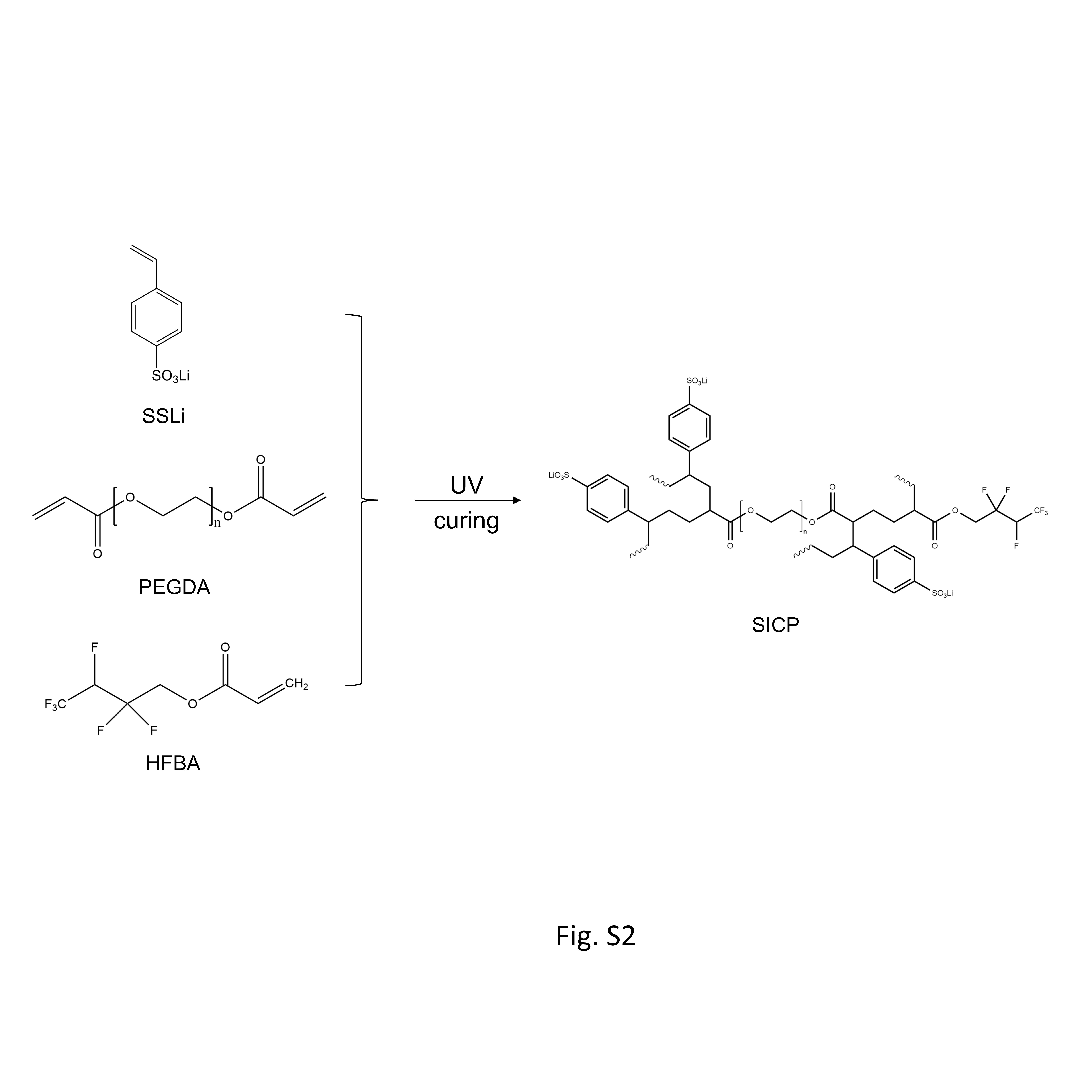


**Figure S2.** The synthesis of PLF electrolyte.


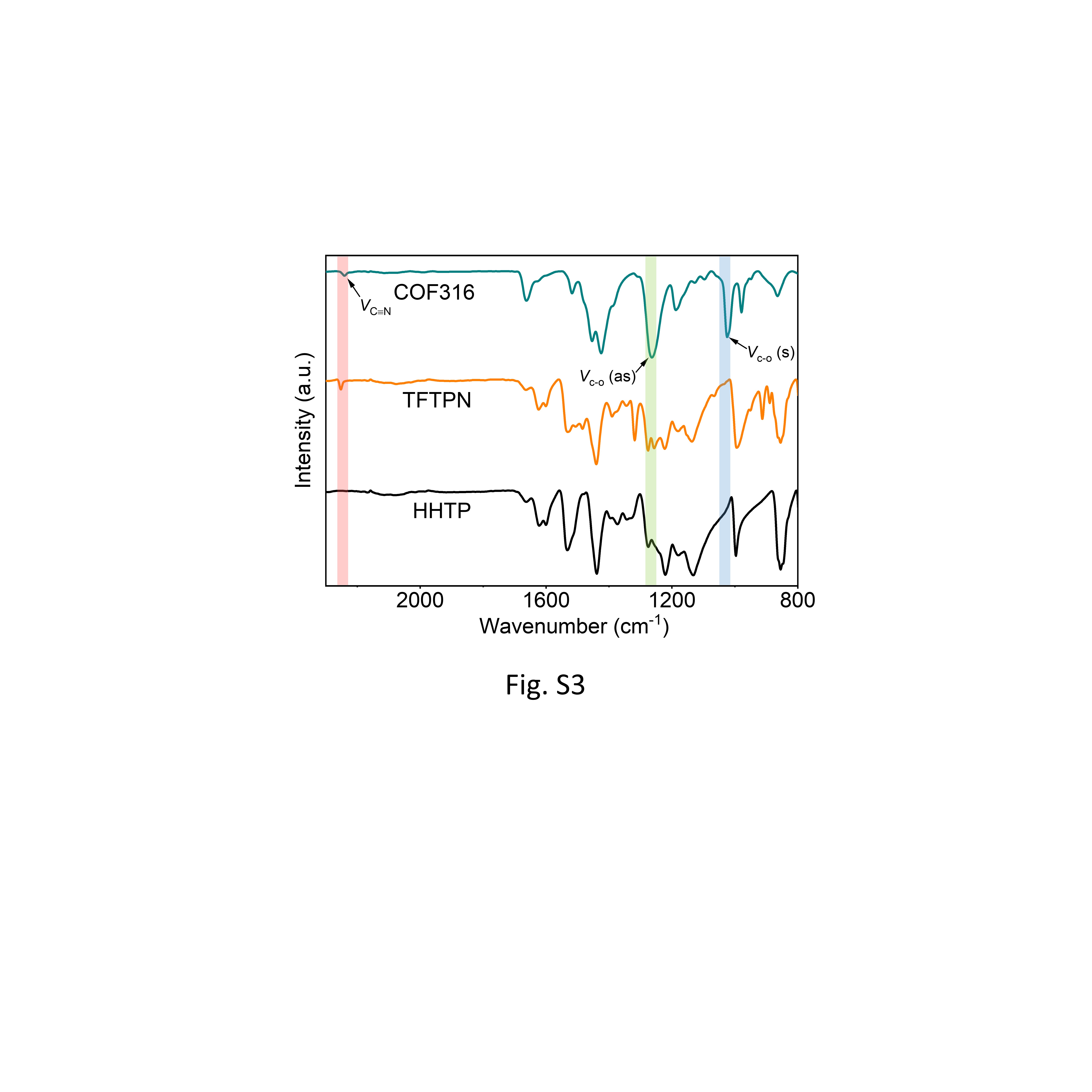


**Figure S3.** FT-IR spectra of COF316, TFTPN, and HHTP.


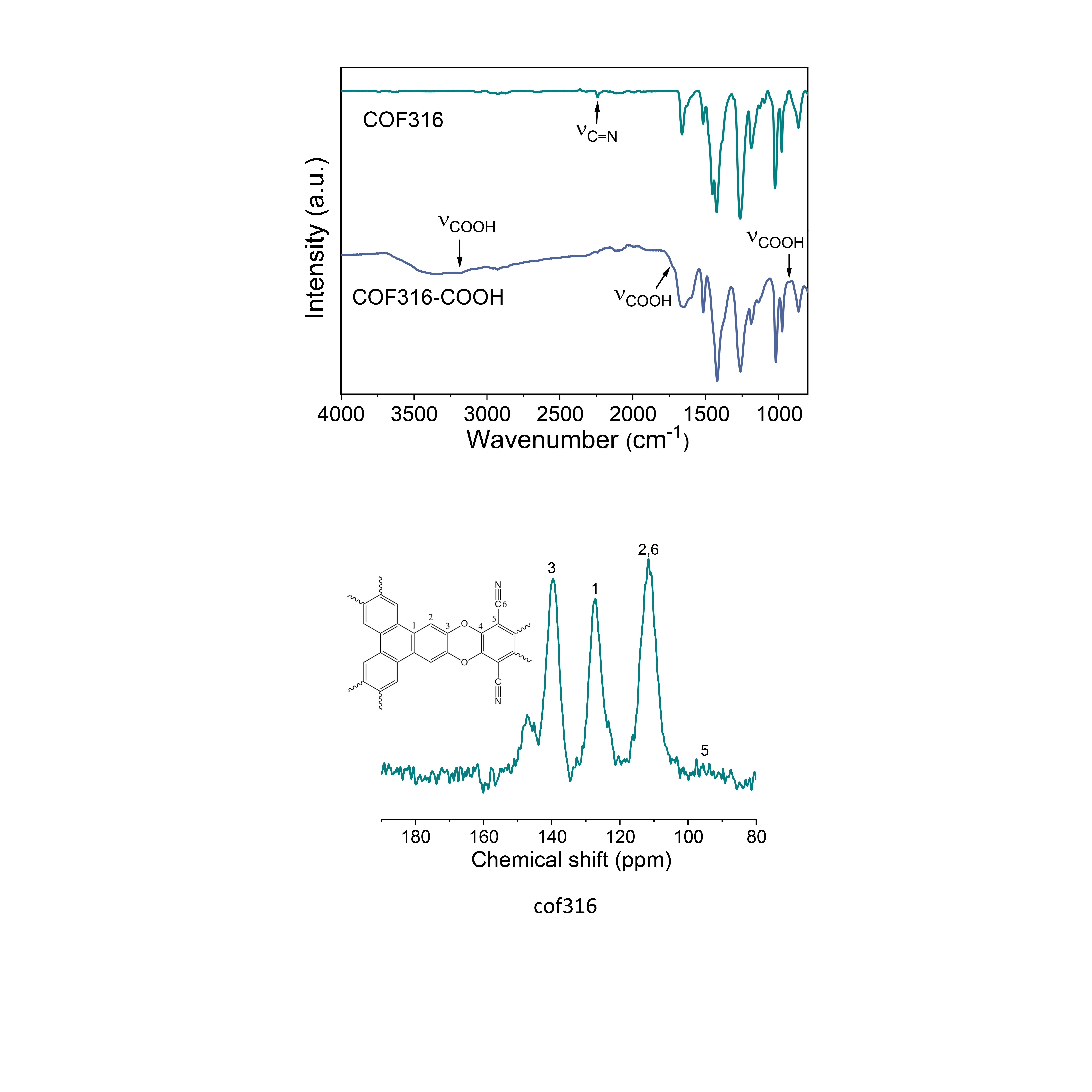


**Figure S4.** Solid-state ^13^C NMR spectrum of COF316.


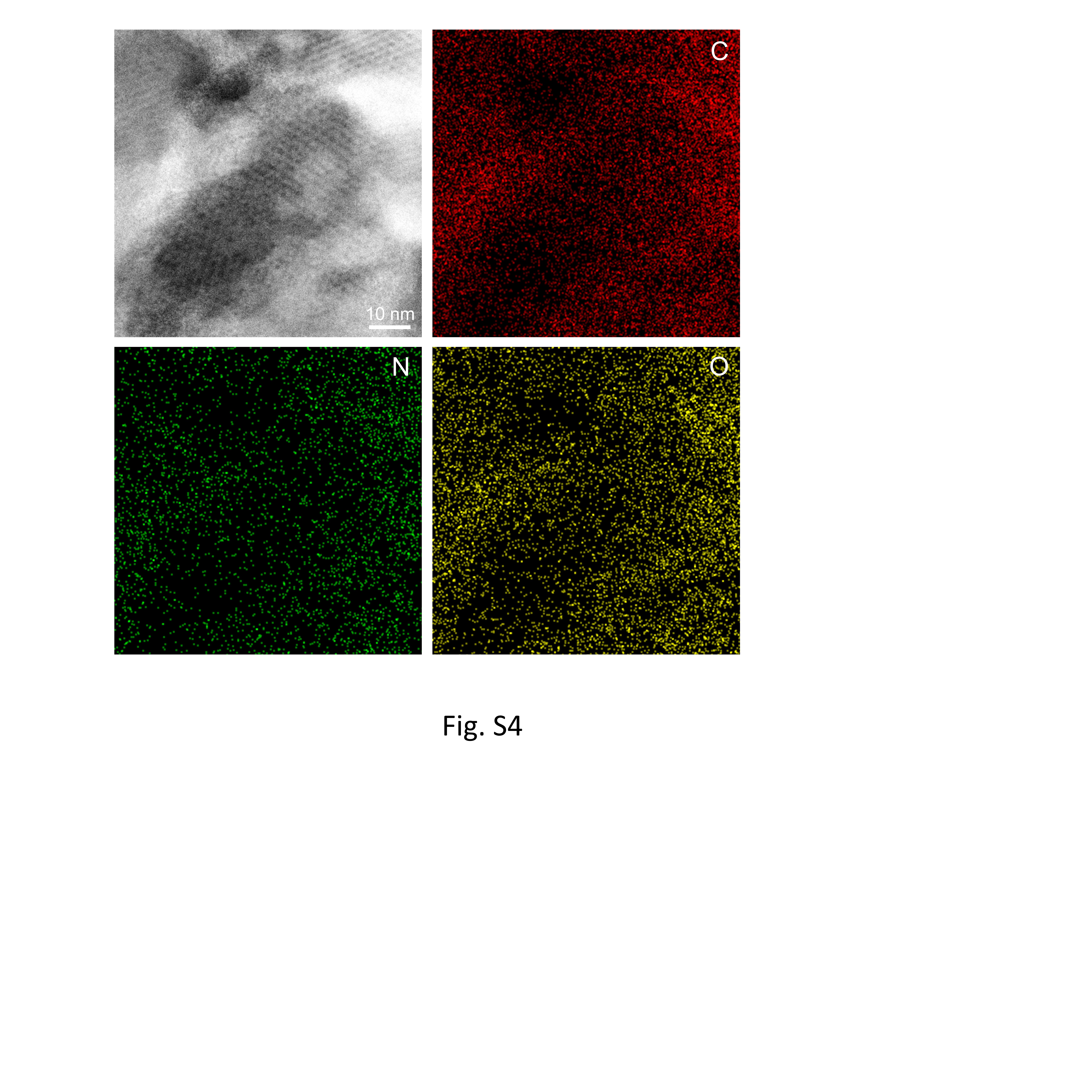


**Figure S5.** Annular dark-field STEM image of COF316 and the corresponding elemental mappings.


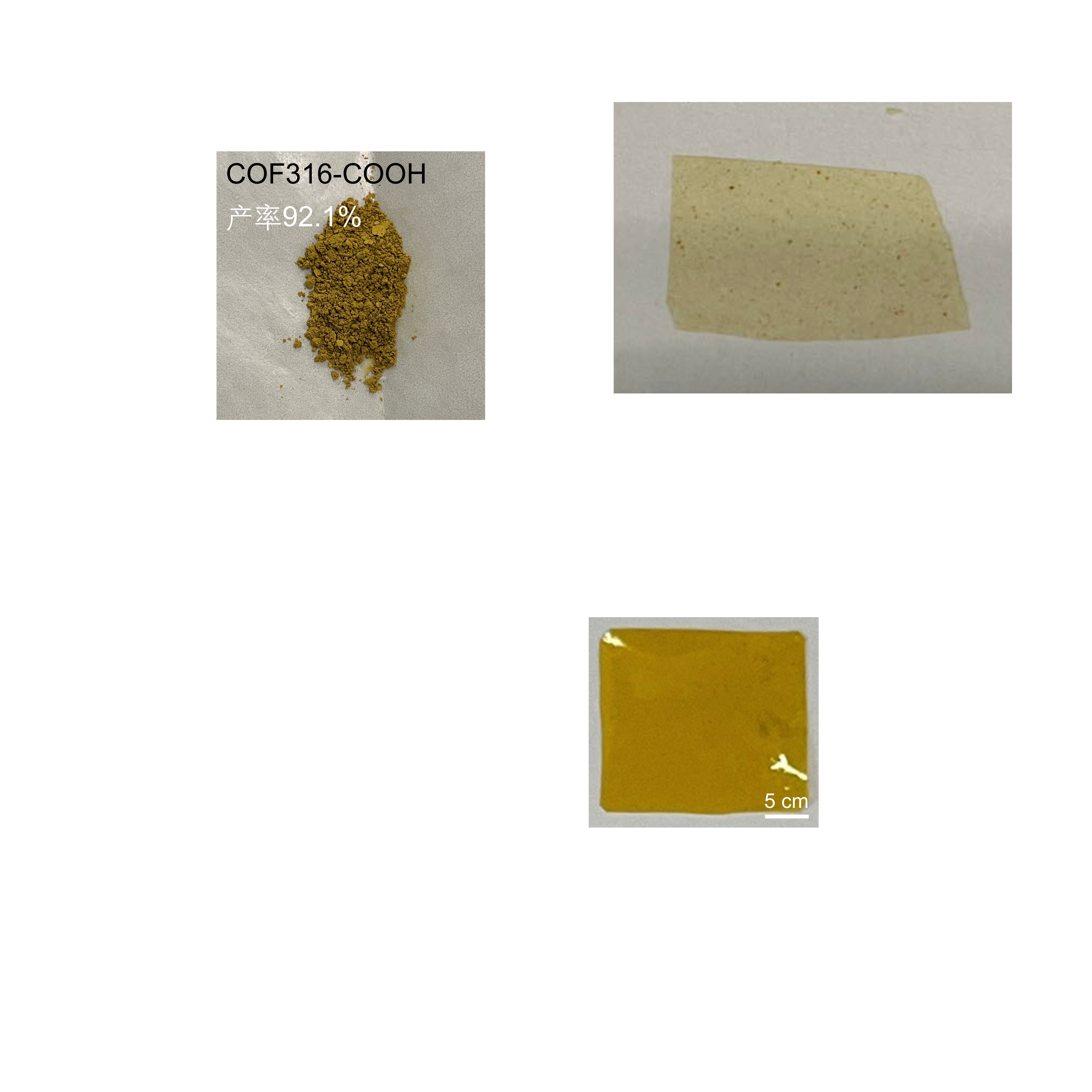


**Figure S6.** Digital photograph of PLF@COF316 film with area of ~ 500 cm^2^.


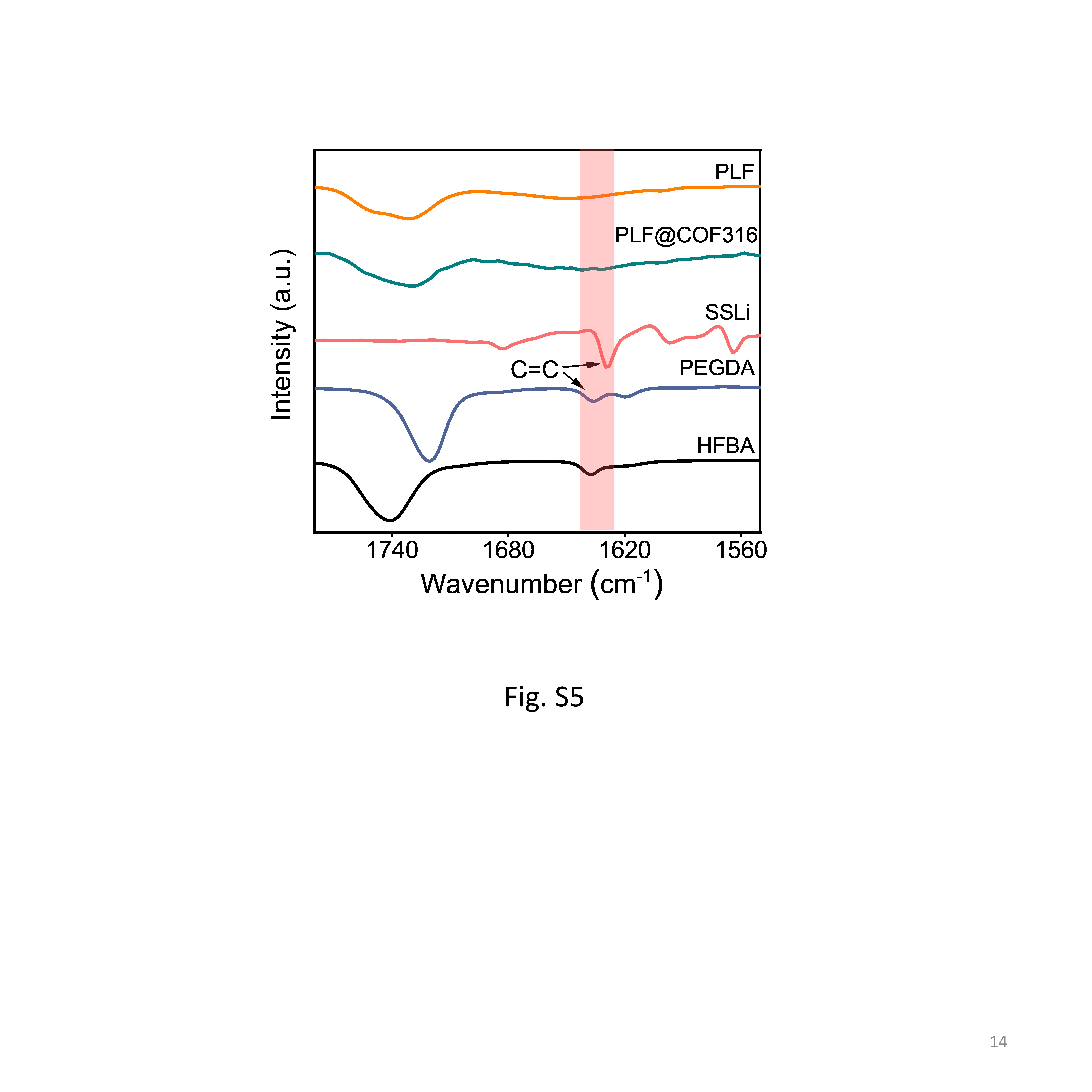


**Figure S7.** FTIR spectra of PLF, PLF@COF316, SSLi, PEGDA, and HFBA.


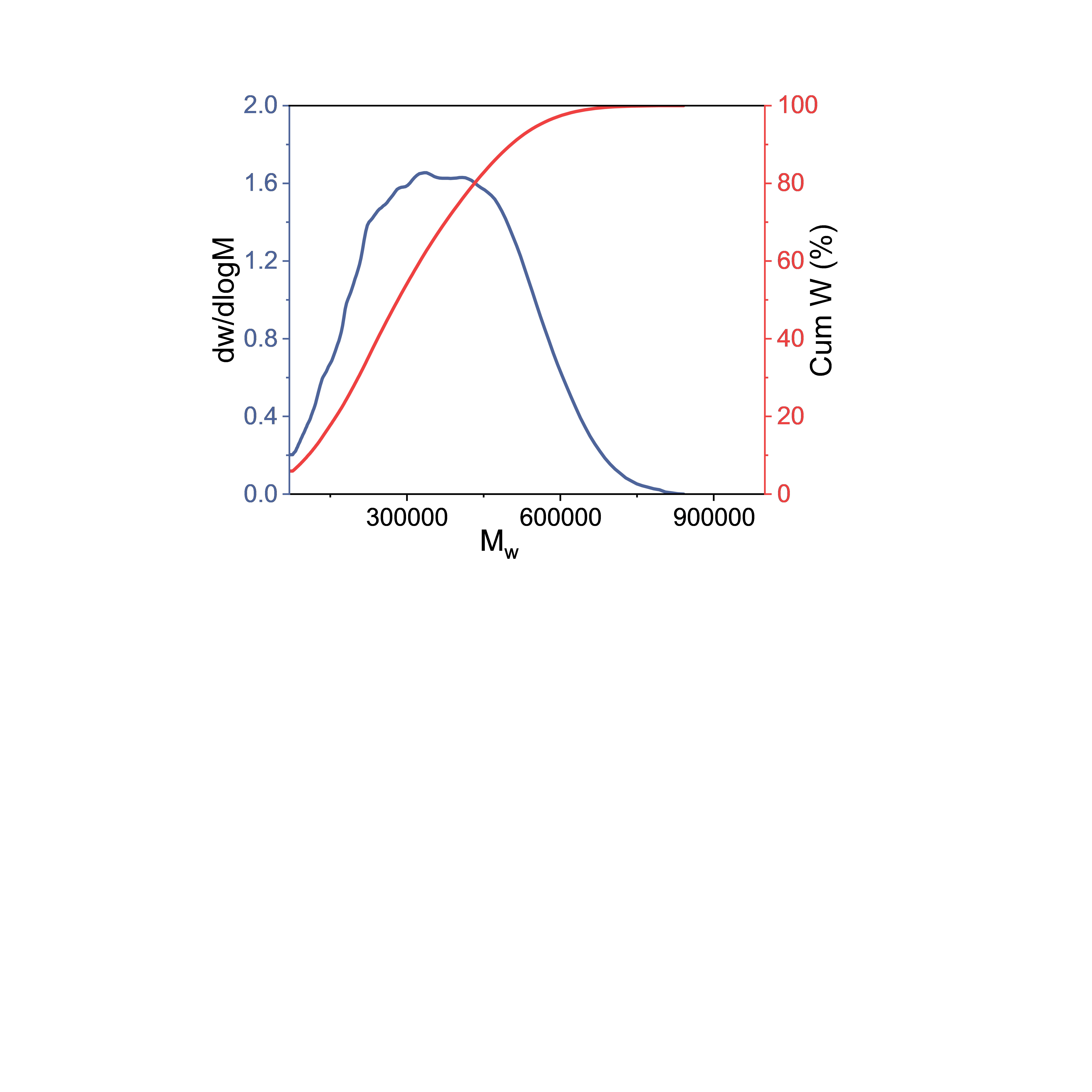


**Figure S8.** GPC result of the PLF@COF316.


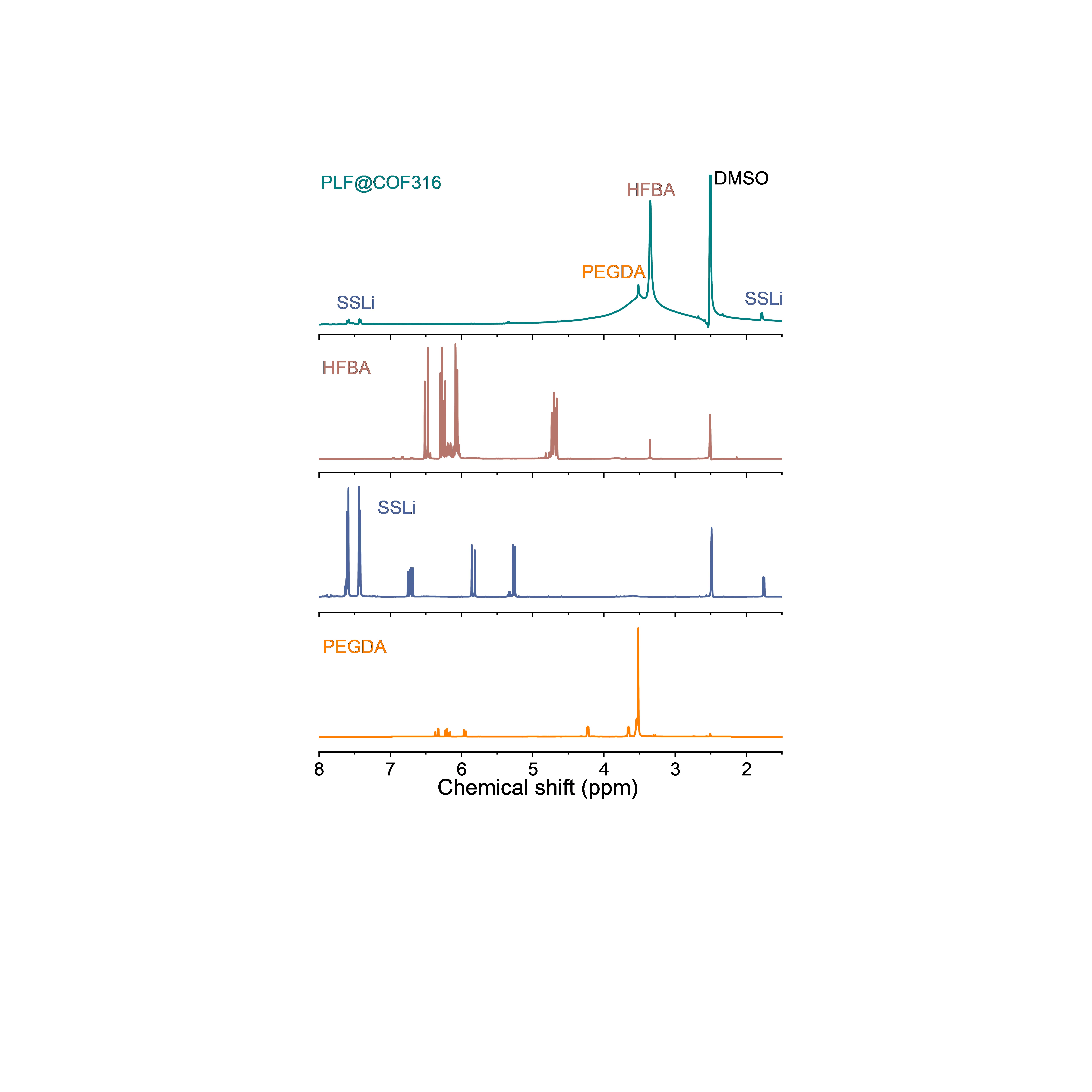


**Figure S9.** ^1^H NMR spectrum of PLF@COF316, HFBA, SSLi, and PEGDA.


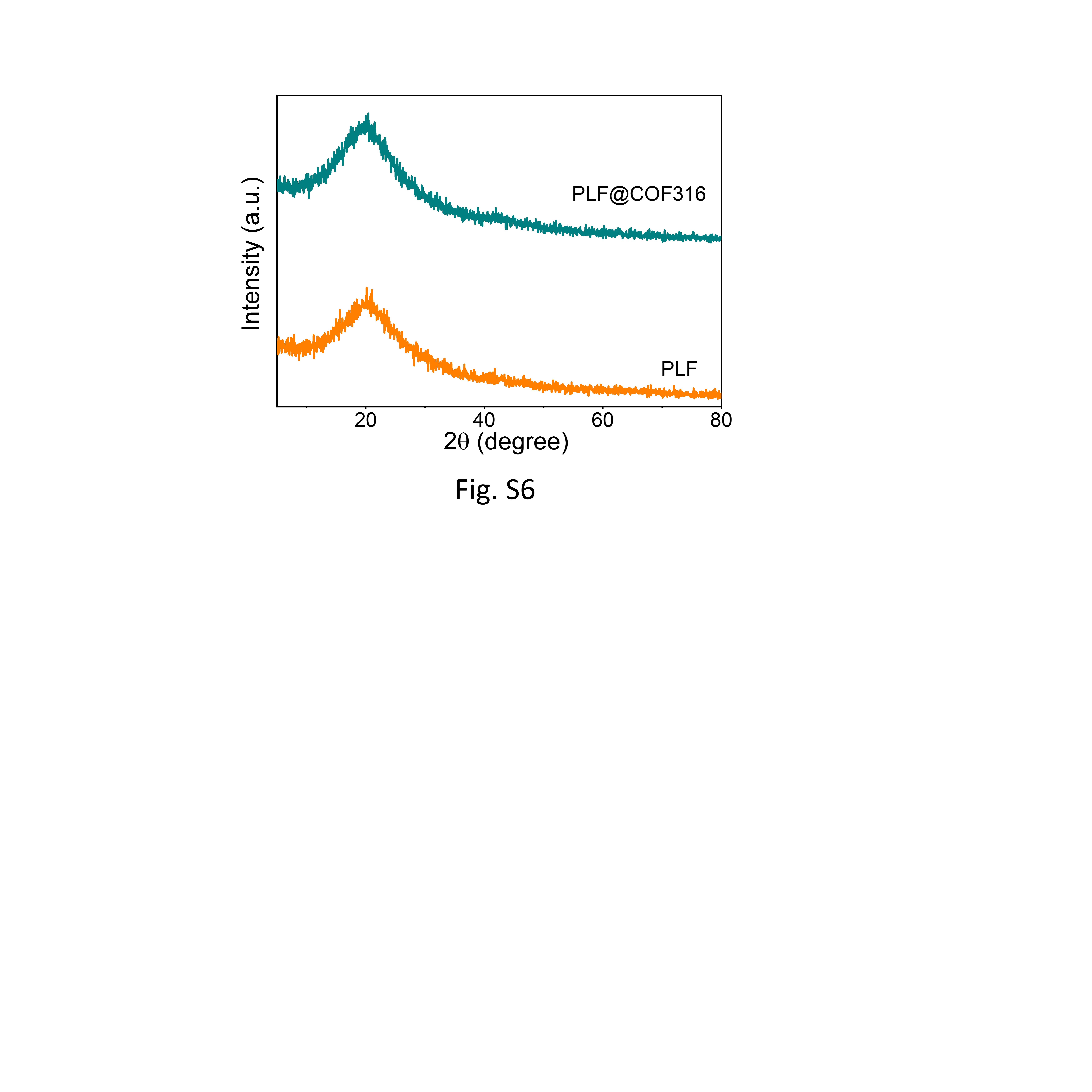


**Figure S10.** XRD patterns of PLF and PLF@COF316.


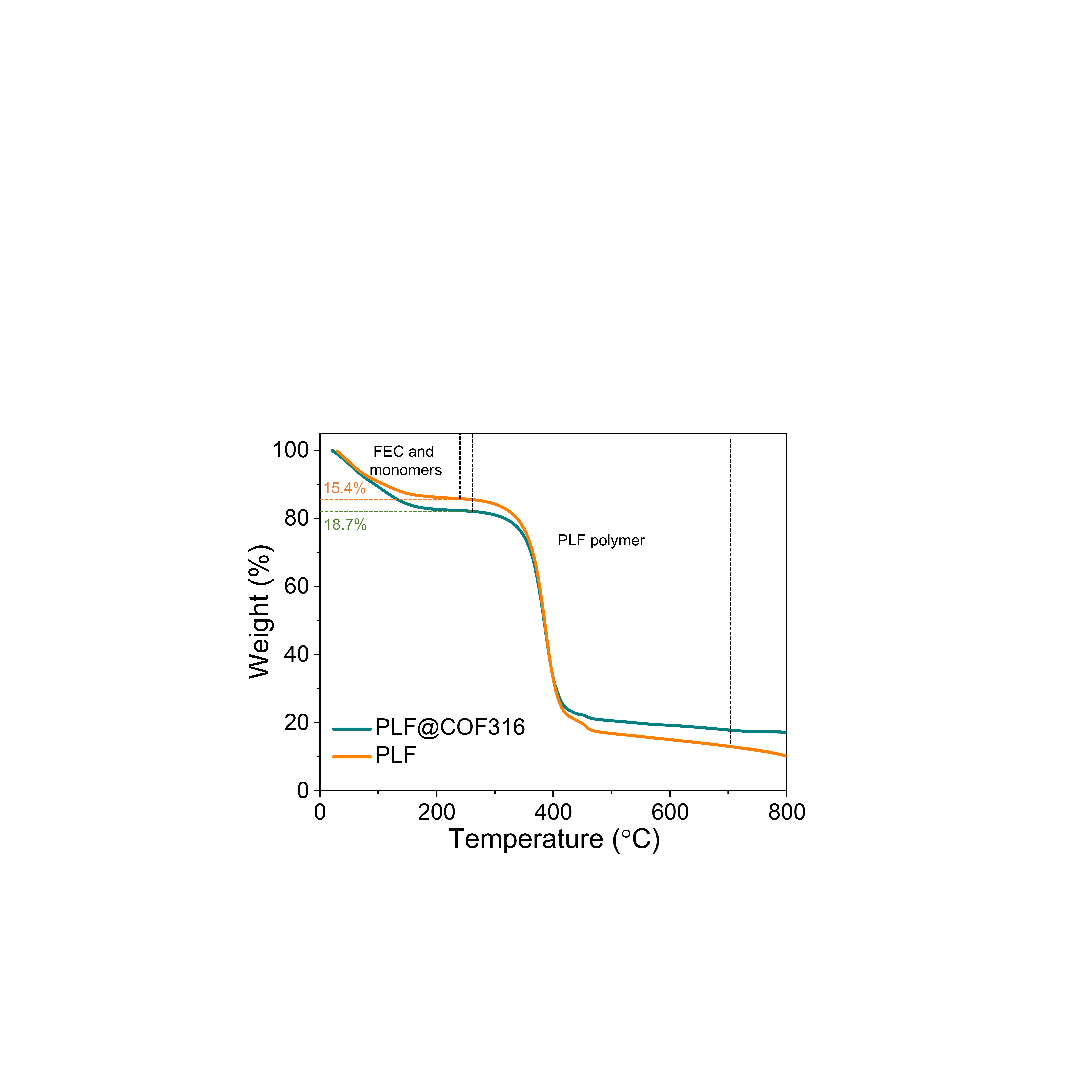


**Figure S11.** Thermogravimetric analysis of PLF@COF316 and PLF electrolytes.

TGA analysis was employed to determine the component of PLF. The mass loss below 225 °C is attributed to FEC and residual monomers. From the TGA analysis, it can be concluded that PLF is composed of polymer chain (~84.6wt%), FEC (~9.1wt%) and residual monomers (~6.3wt%). PLF@COF316 is composed of polymer chain (~79.3wt%), COF316 (~2.0wt%), plasticizer of FEC (~9.1wt%) and residual monomers (~6.3wt%). Another mass loss of 3.3wt% in PLF@COF316 below 225 °C maybe come from the moisture adsorbed by the developed channels of COF316. Therefore, the degree of polymerization is calculated to be 93.1% according to the following formula.

Degree of polymerization=$\frac{Weight of polymer chain}{Weight of polymer chainWeight of residual monomers}$


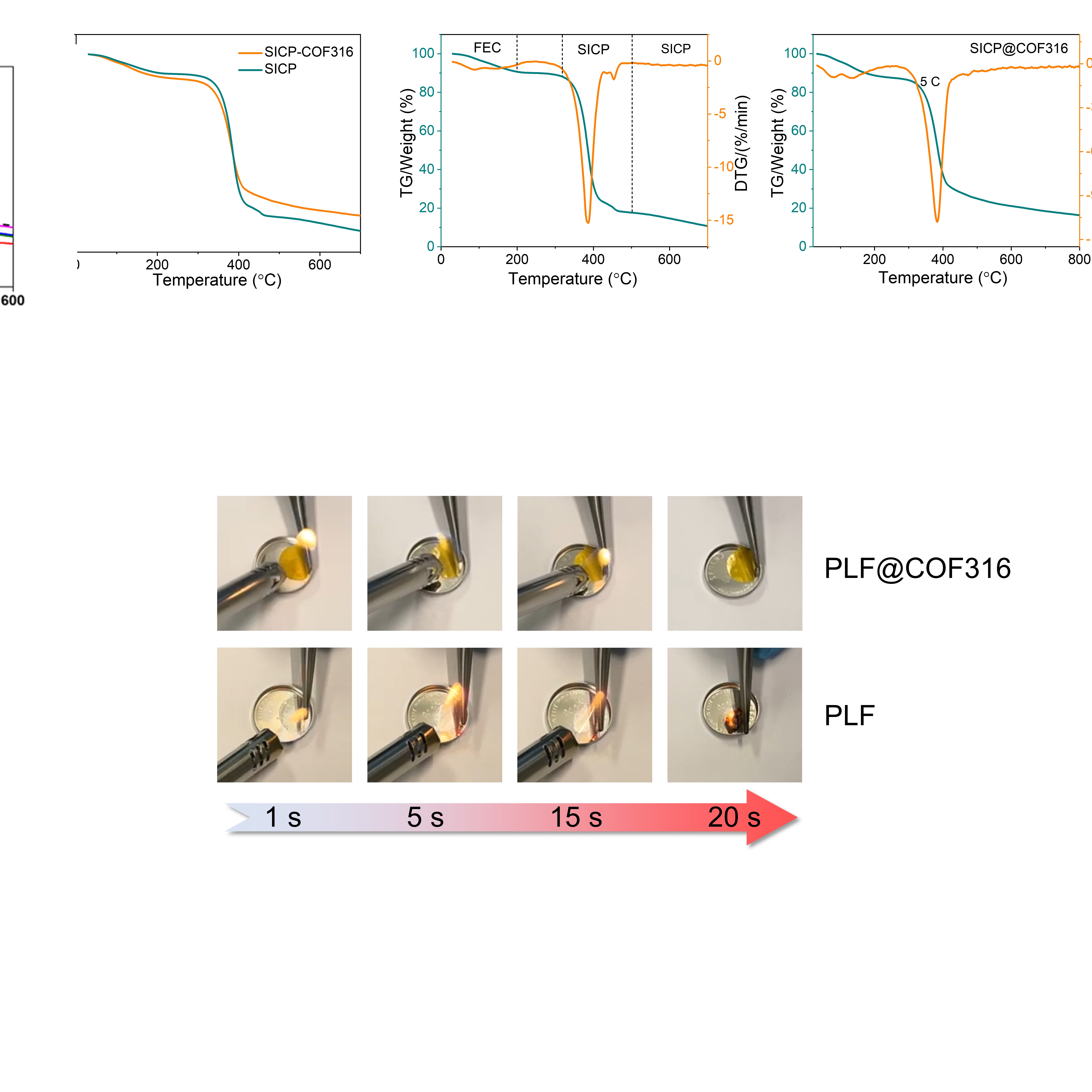


**Figure S12.** Ignition test of PLF@COF316 and PLF electrolytes.


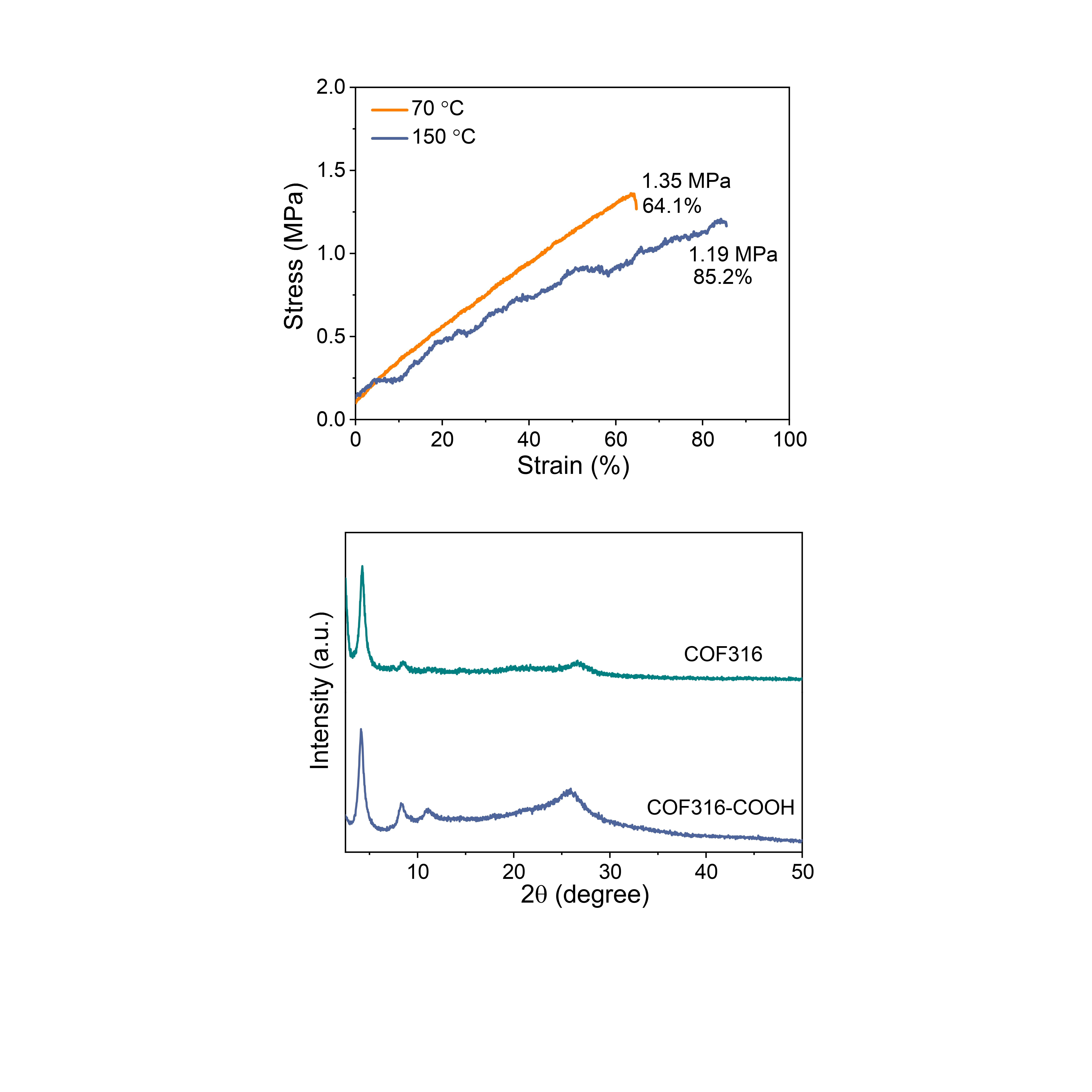


**Figure S13.** The stress-strain curves of PLF@COF316 at 70 °C and 150 °C.


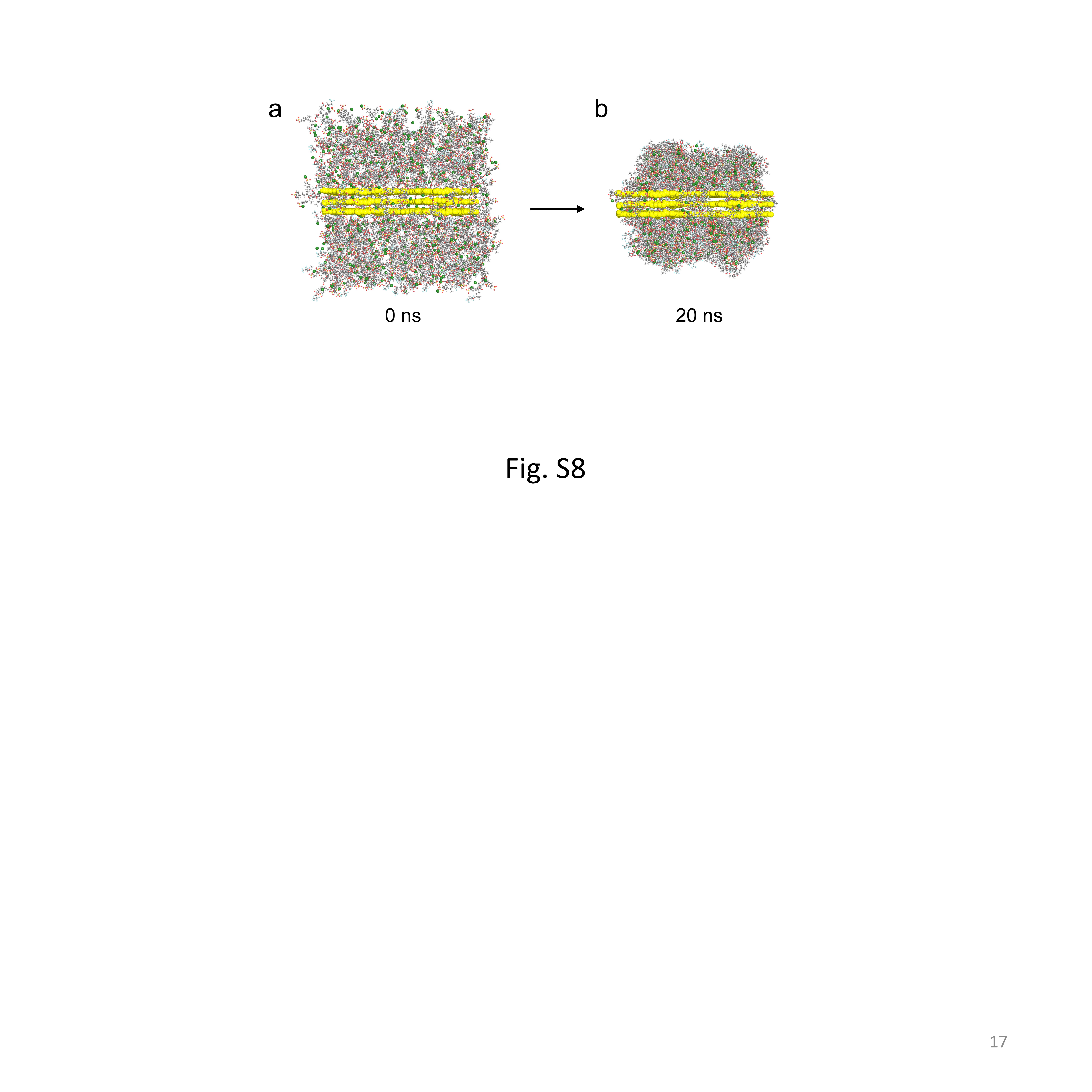


**Figure S14.** Side-view of the conformation evolution of PLF@COF316 system at a) 0 ns and b) 20 ns based on MD simulations.


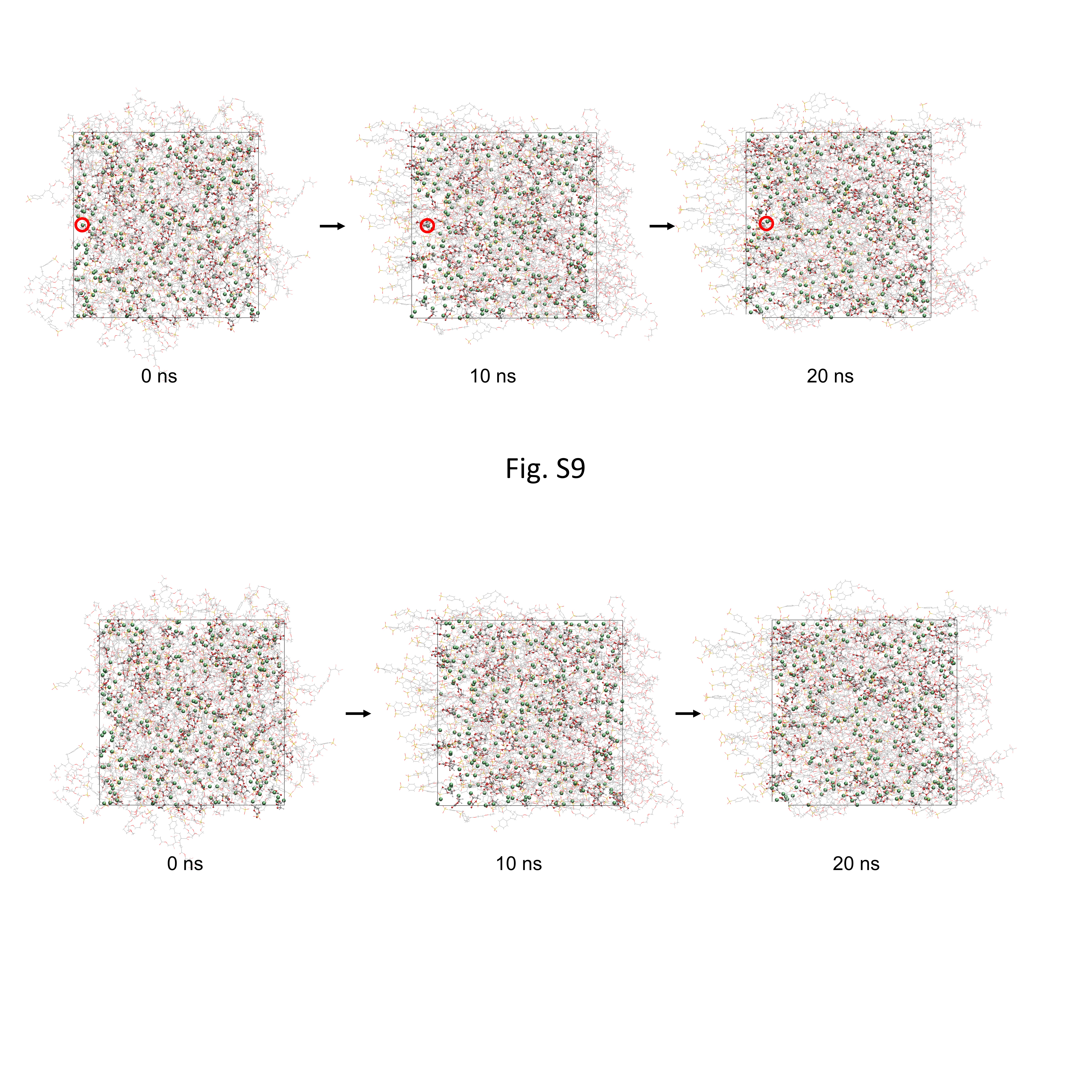


**Figure S15.** Simulation snapshots of the Li^+^ migration in PLF system.


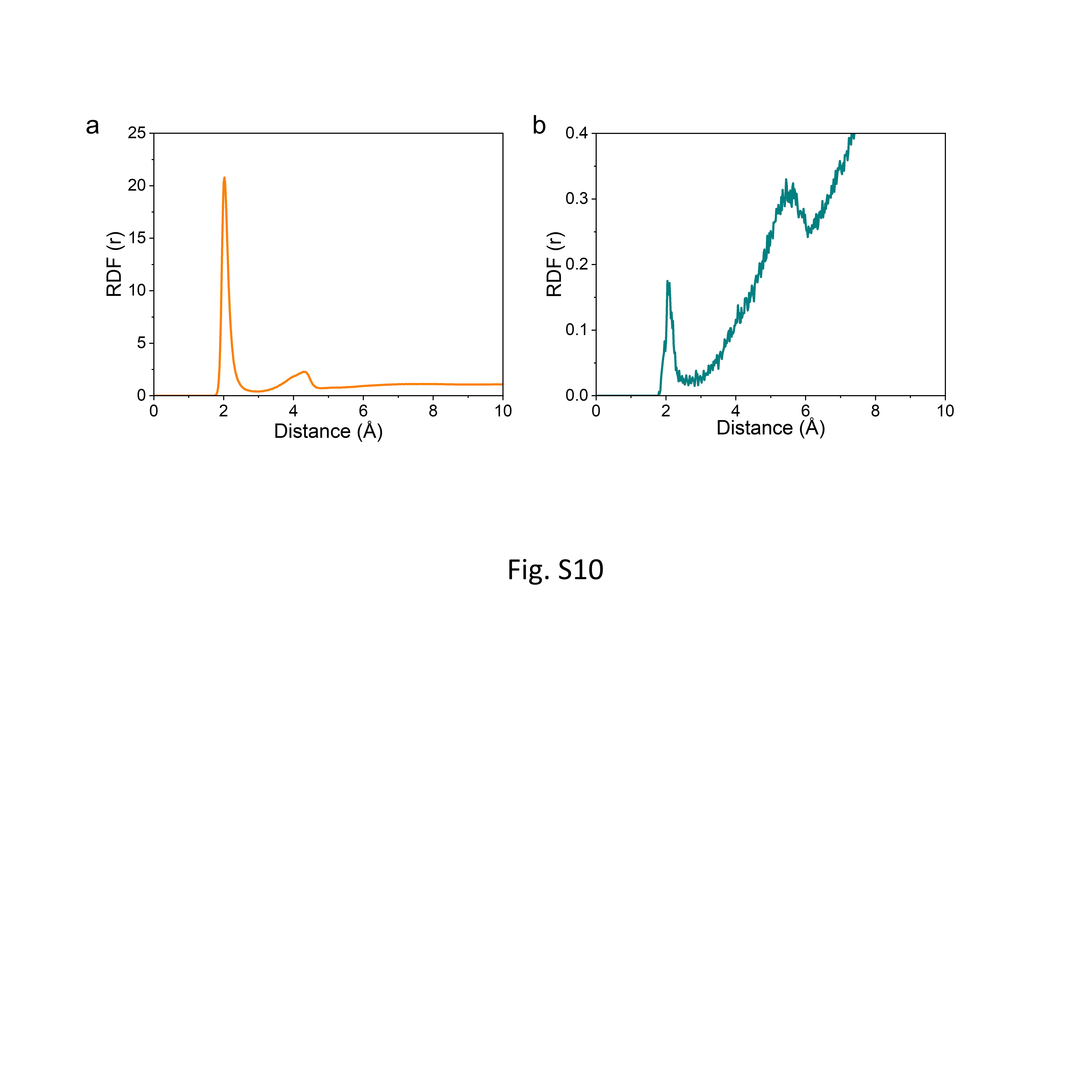


**Figure S16.** Calculated RDFs of a) Li-O (PLF) and b) Li-O (COF316) in PLF@COF316 system.


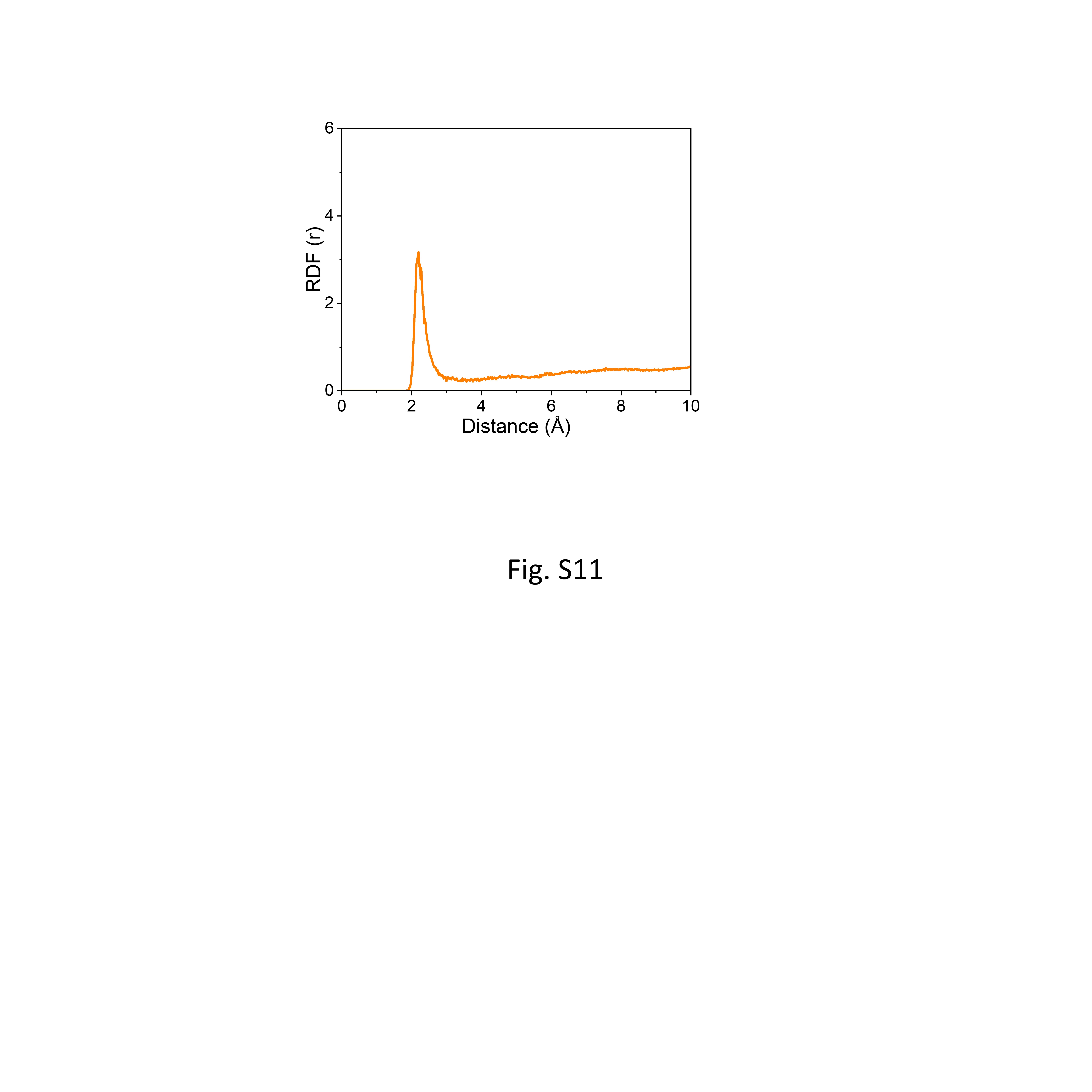


**Figure S17.** Calculated RDF of Li-N (COF316) in PLF@COF316 system.


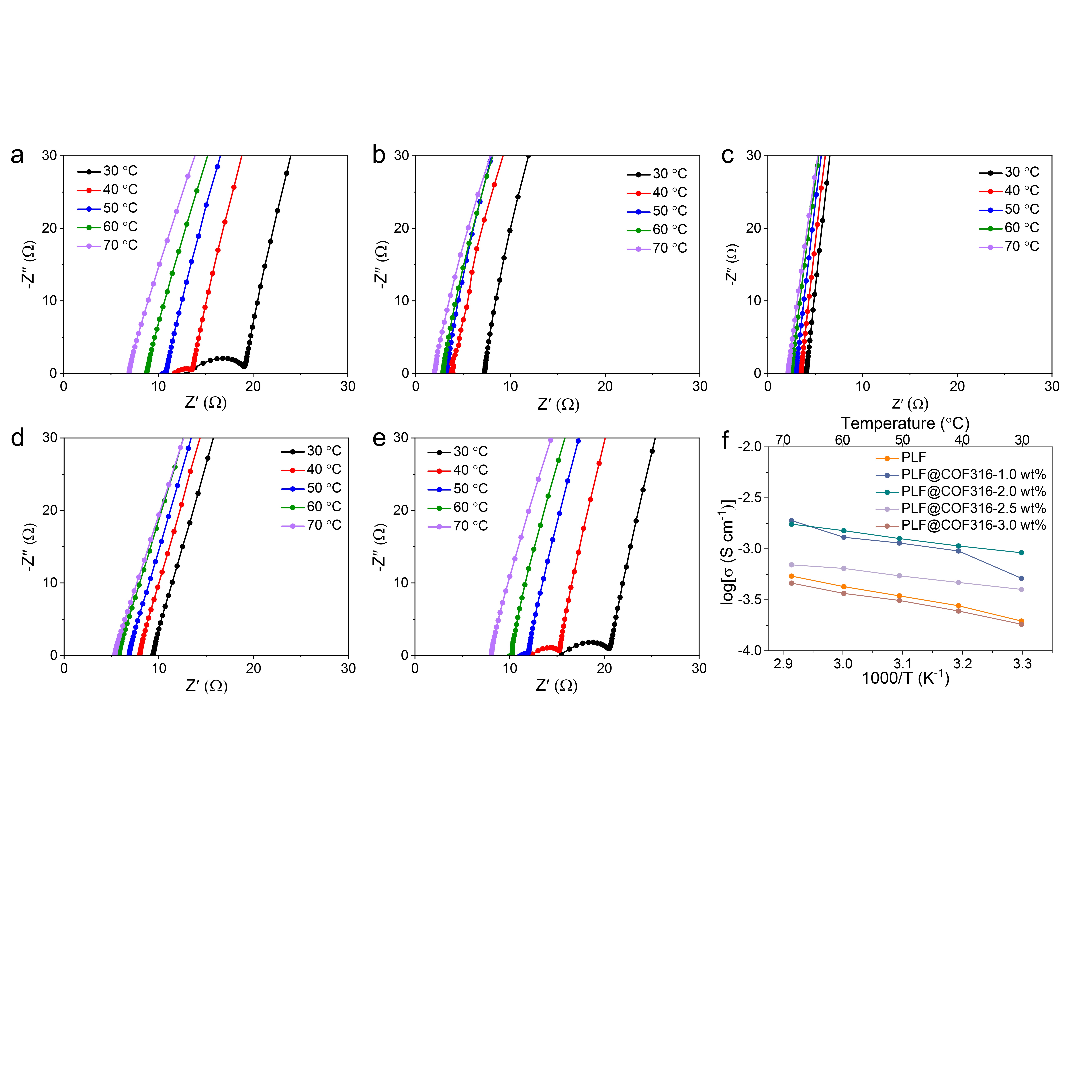


**Figure S18.** EIS curves of PLF@COF316 with COF316 contents of (a) 0 wt%, (b) 1.0 wt%, (c) 2.0 wt%, (d) 2.5 wt%, and (e) 3.0 wt%. (f) Ionic conductivity-temperature functions of PLF@COF316 with various COF316 contents.


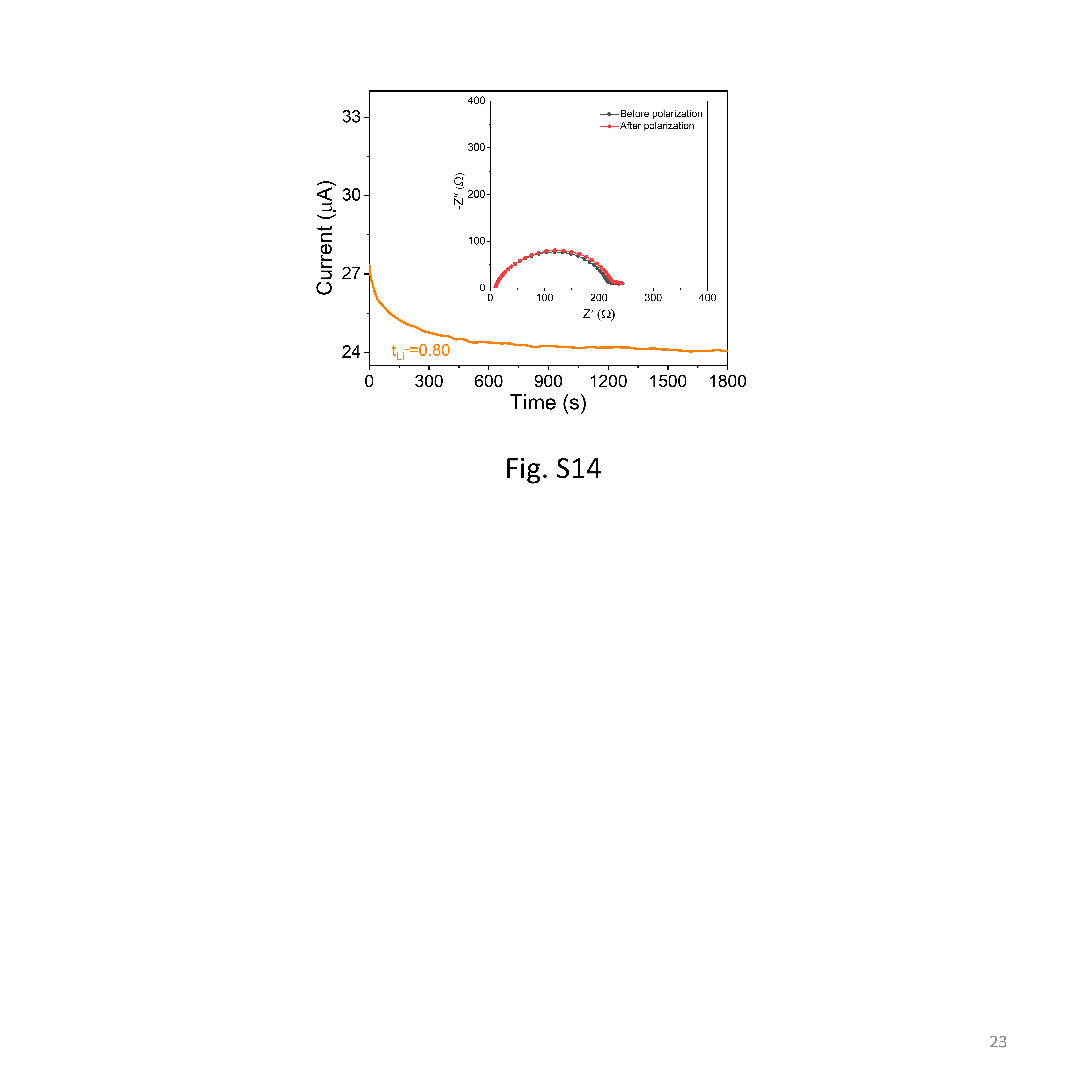


**Figure S19.** Chronoamperometry polarization curve and the impedance spectra before and after polarization of Li|PLF|Li symmetric cell.


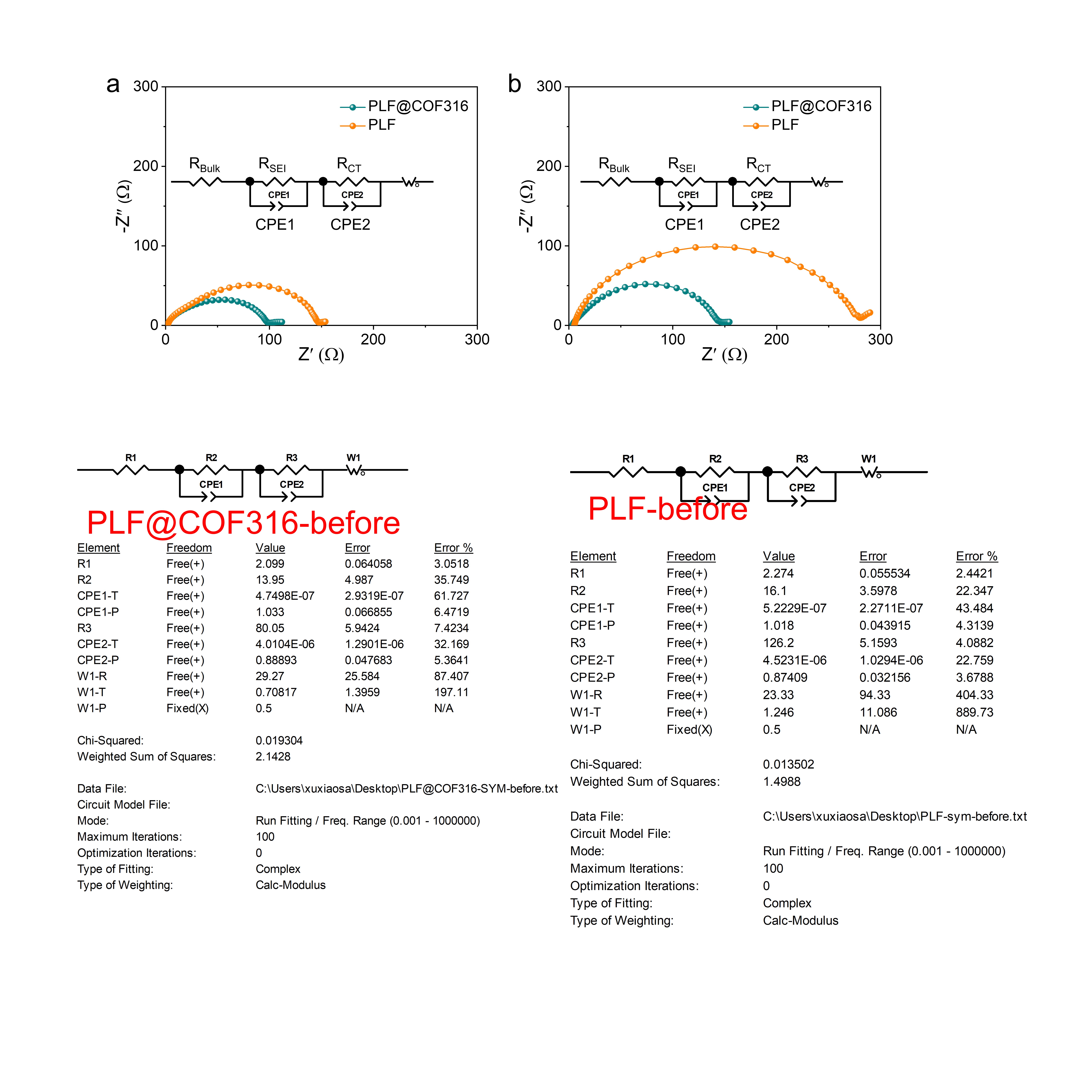


**Figure S20.** EIS curves of Li||Li symmetric cells with PLF@COF316 and PLF electrolytes before and after 500 cycles (the inset is the equivalent circuit simulations).


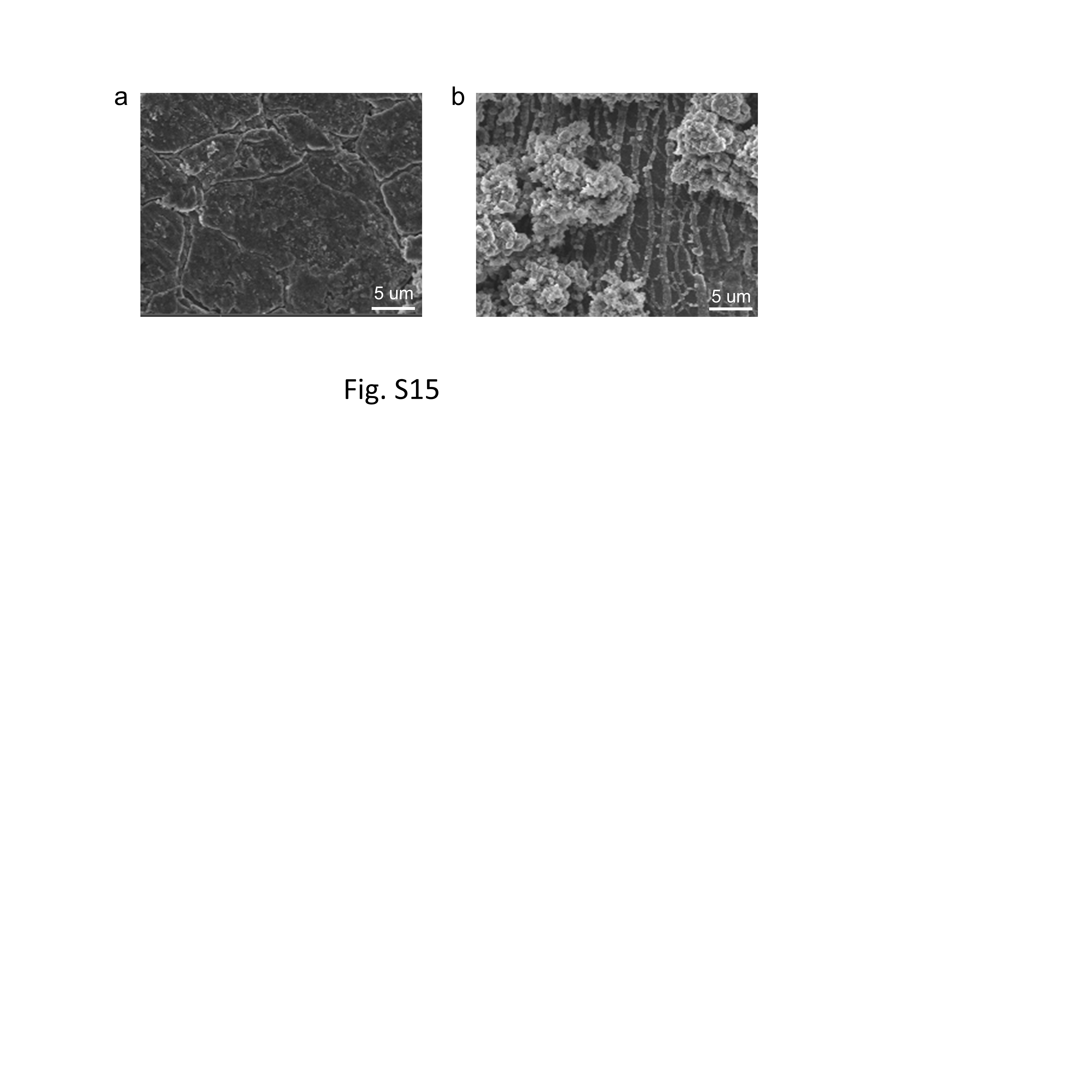


**Figure S21.** Surface SEM image of the cycled Li-anode using a) PLF@COF316 and b) PLF.


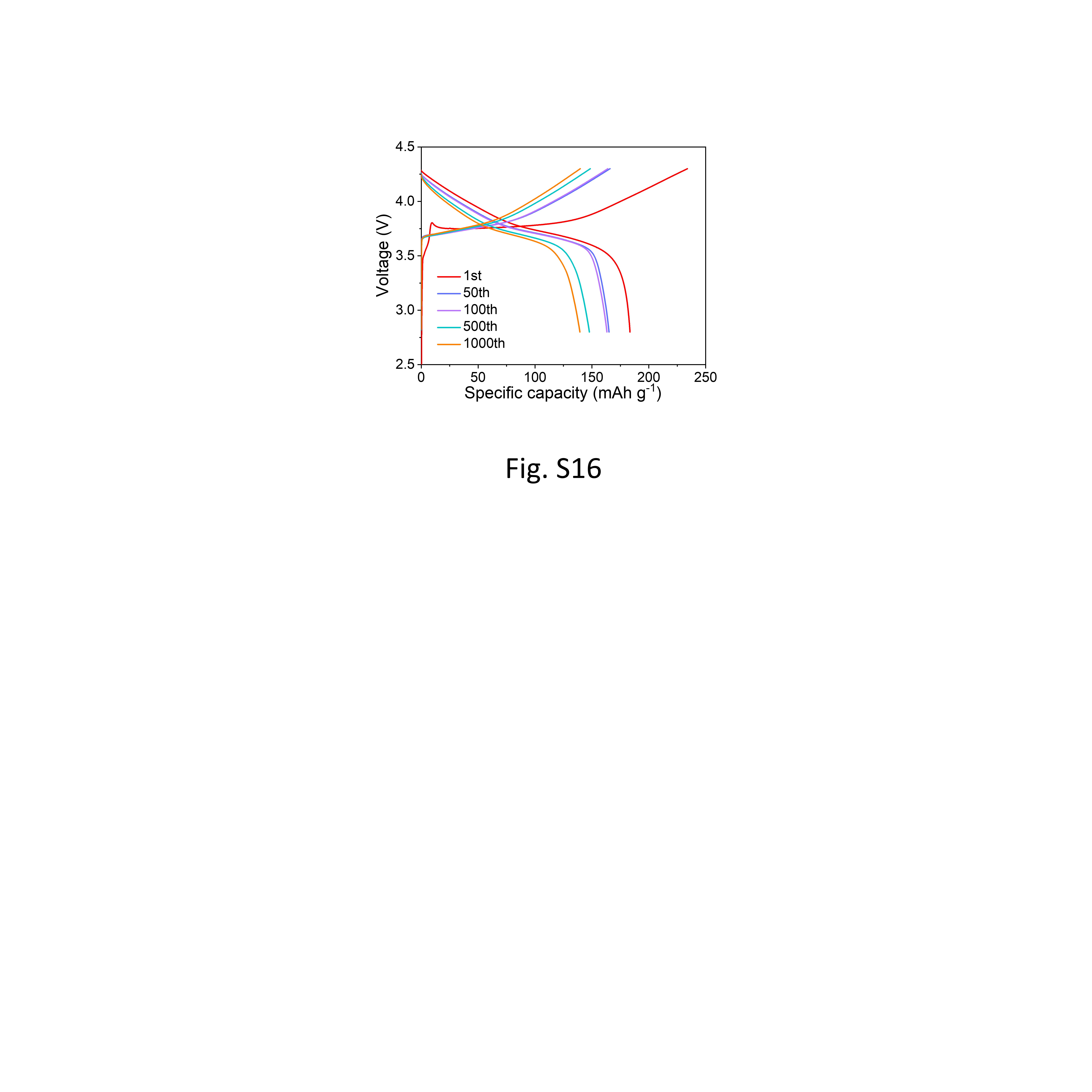


**Figure S22.** The corresponding charge-discharge profiles of NCM622|PLF@COF316|Li cell at different cycles.


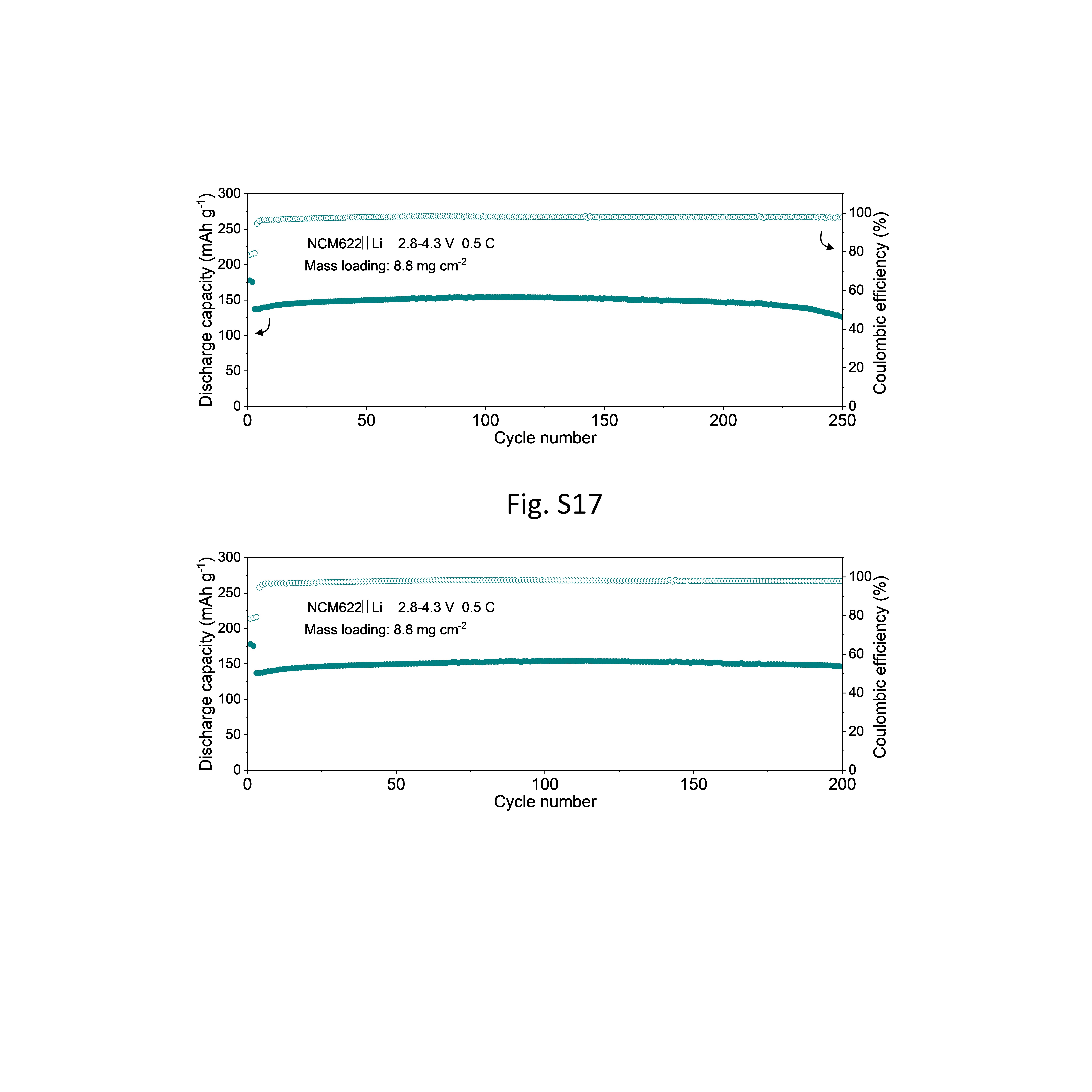


**Figure S23.** Cycling performance of NCM622|PLF@COF316|Li cell at high loading.


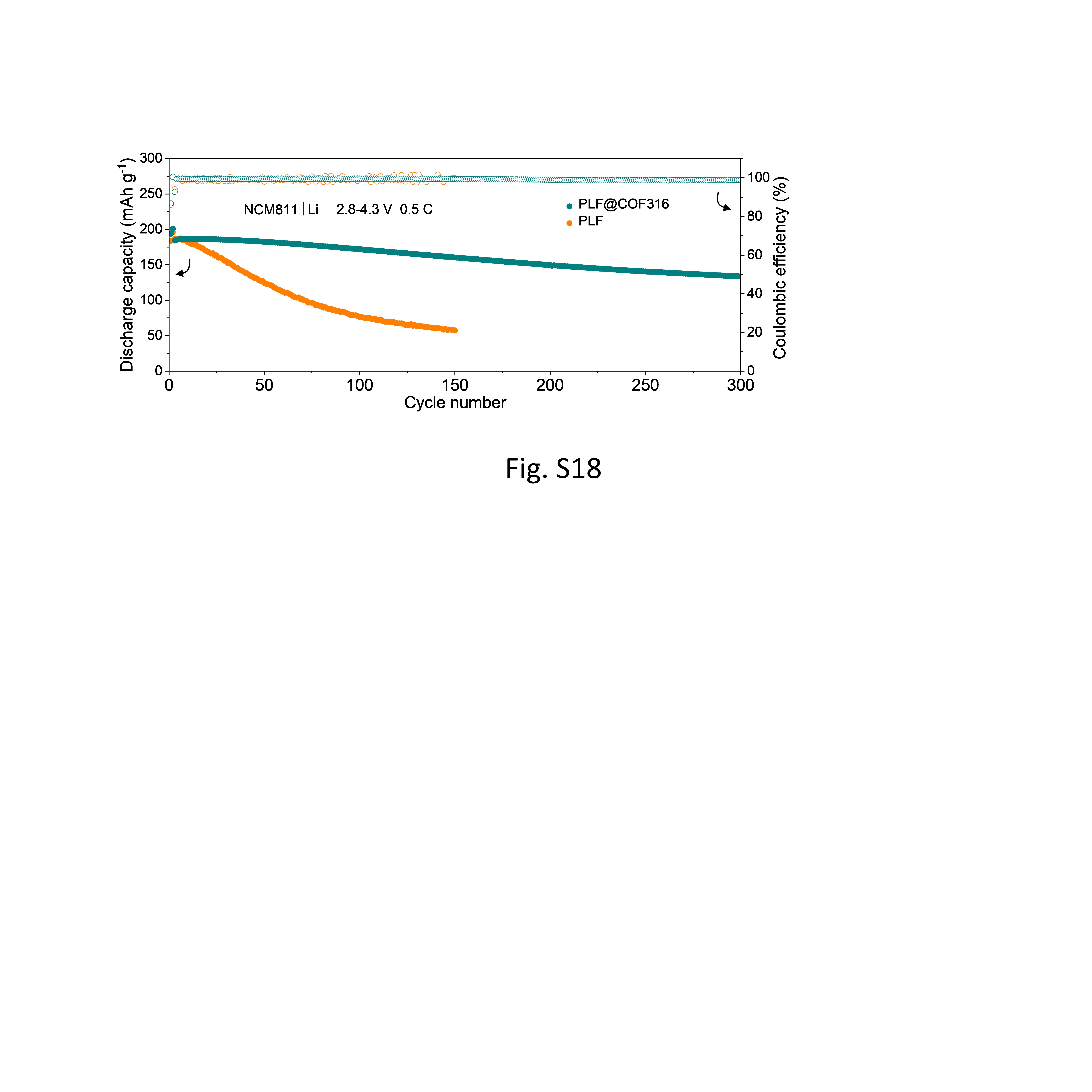


**Figure S24.** Cycling performance of NCM811||Li cells with PLF@COF316 and PLF electrolytes at 0.5 C.


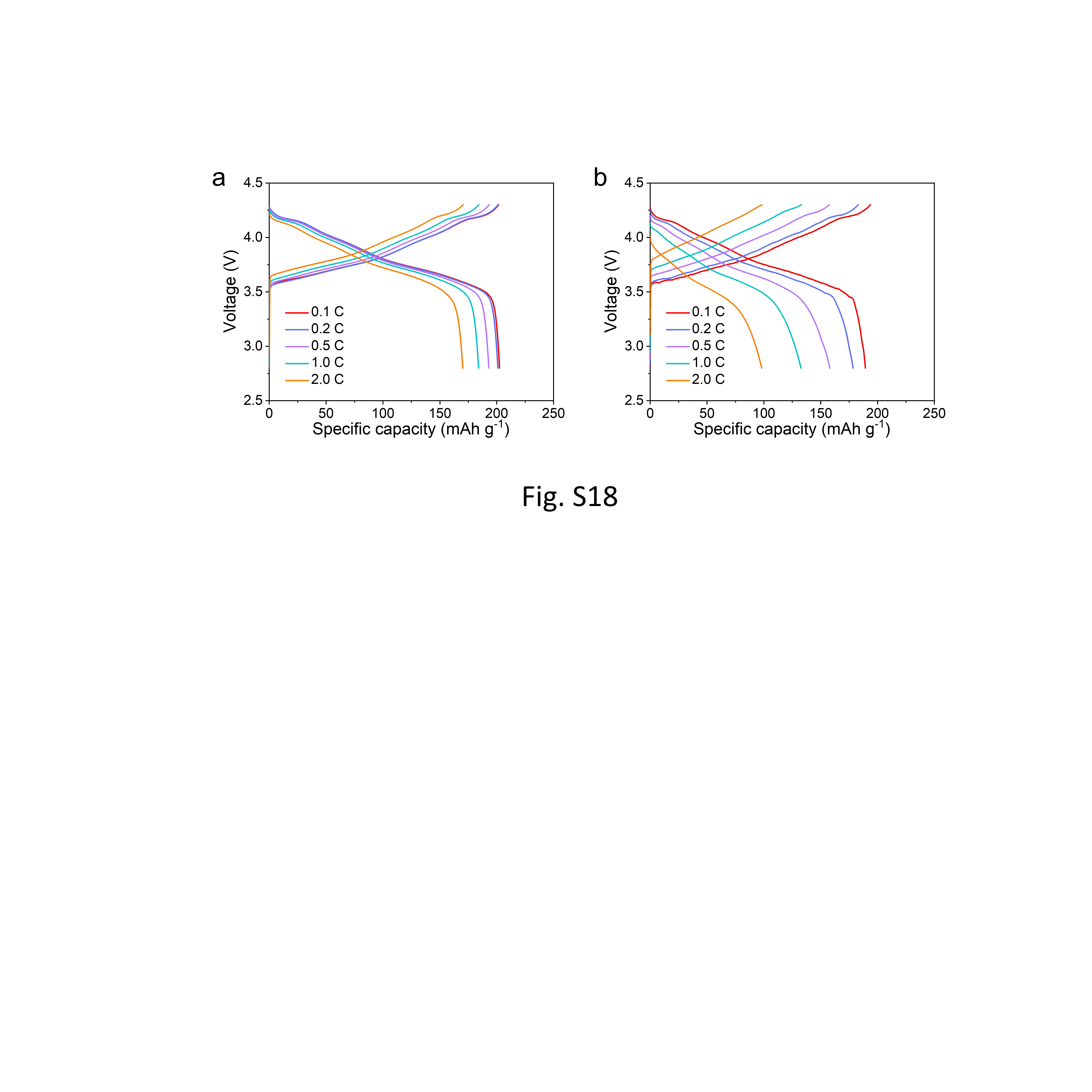


**Figure S25.** The corresponding charge-discharge profiles of NCM811||Li cells with a) PLF@COF316 and b) PLF electrolytes at different current densities.


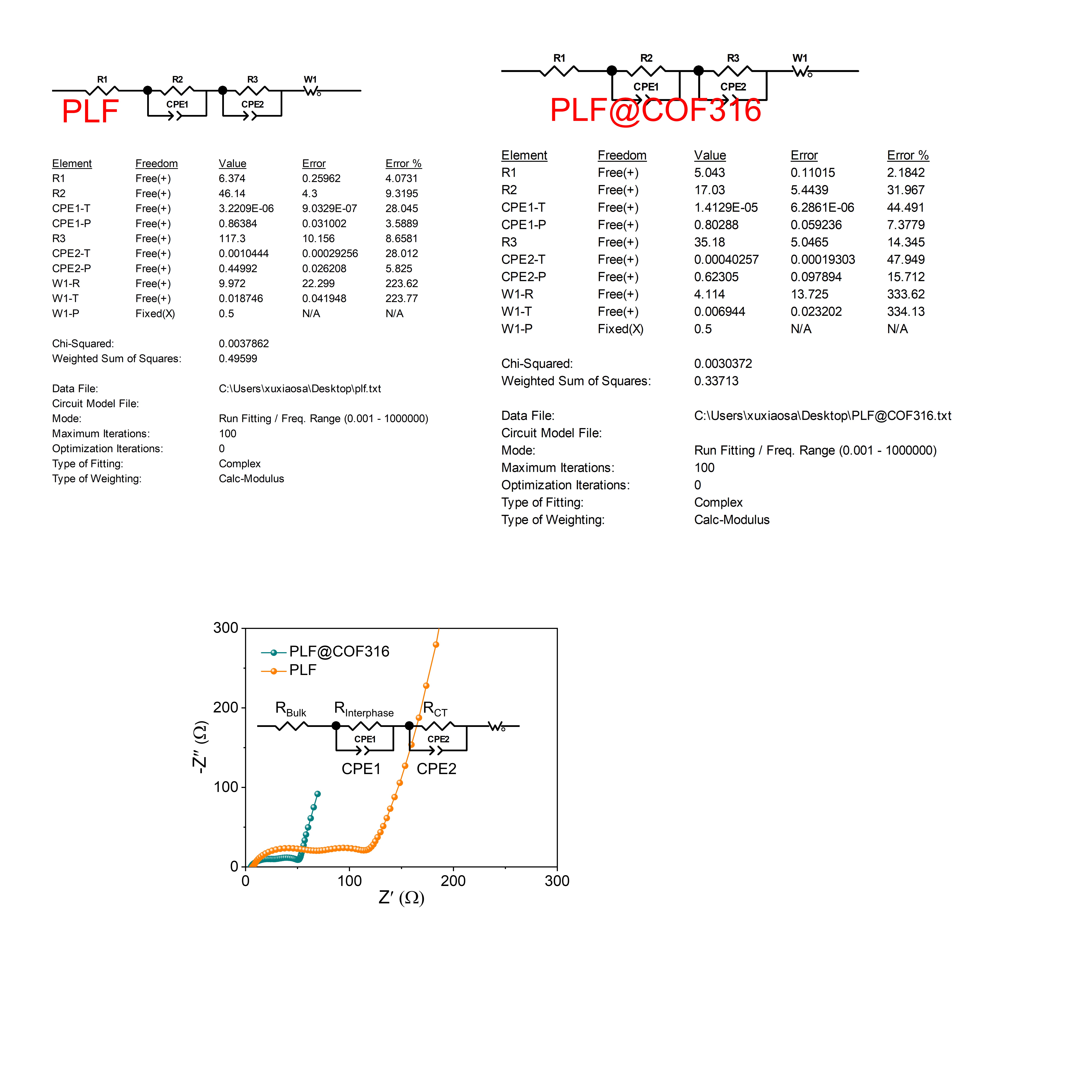


**Figure S26.** EIS curves of NCM811||Li full cells with PLF@COF316 and PLF electrolytes after the rate performance test (the inset is the equivalent circuit simulation).


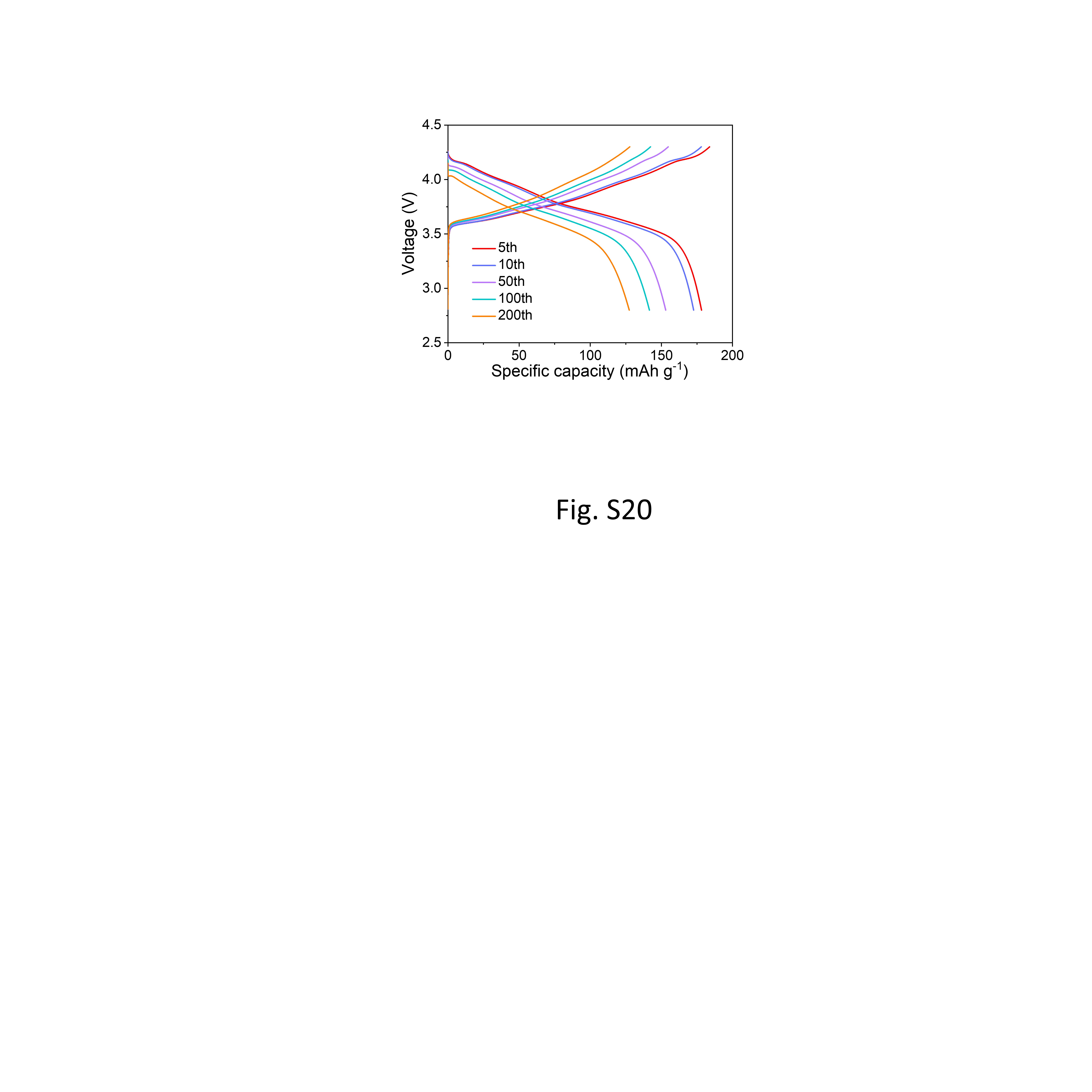


**Figure S27.** The corresponding charge-discharge profiles of NCM811|PLF@COF316|Li pouch cell at different cycles.


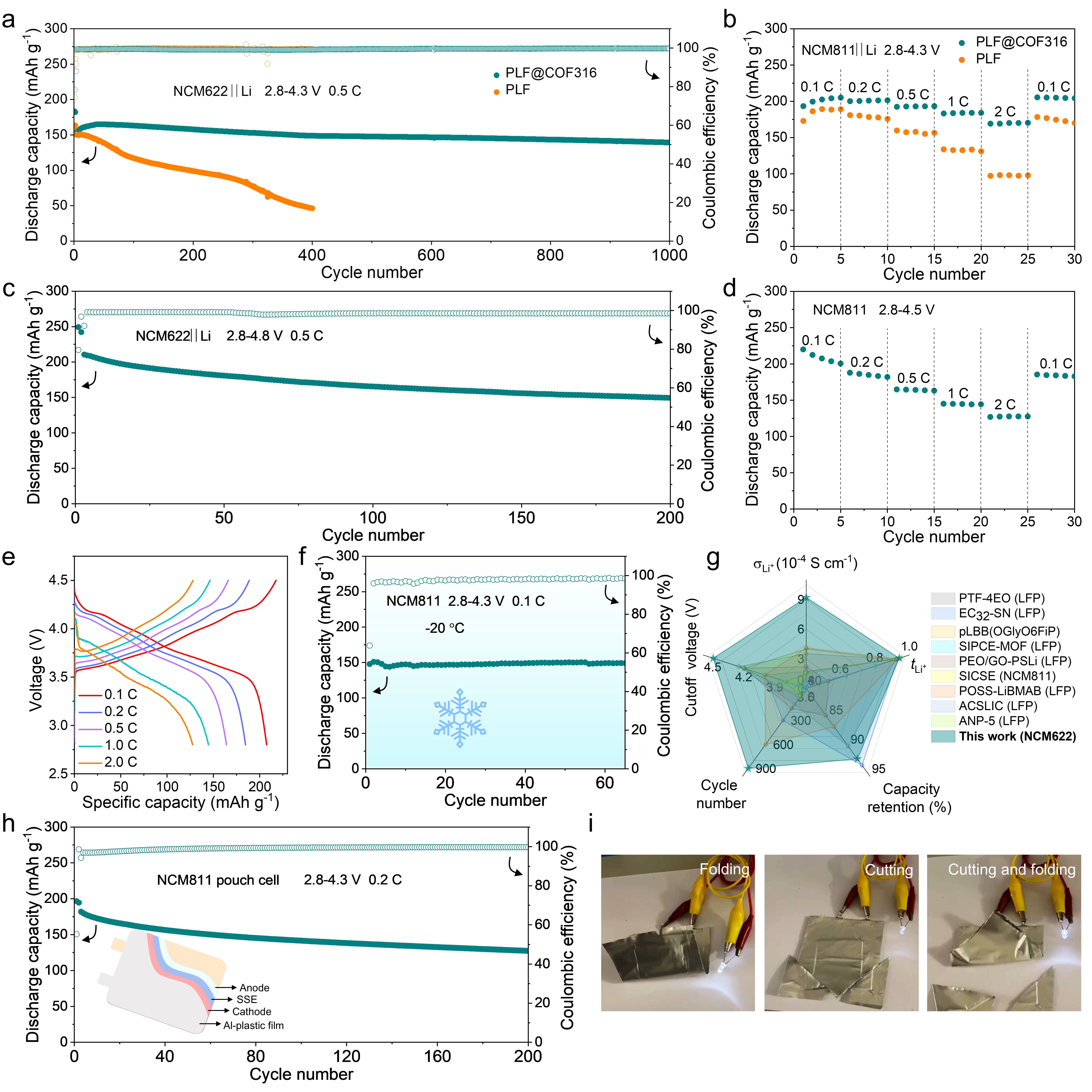


**Figure S28.** The security tests by lighting LED under extreme circumstances.


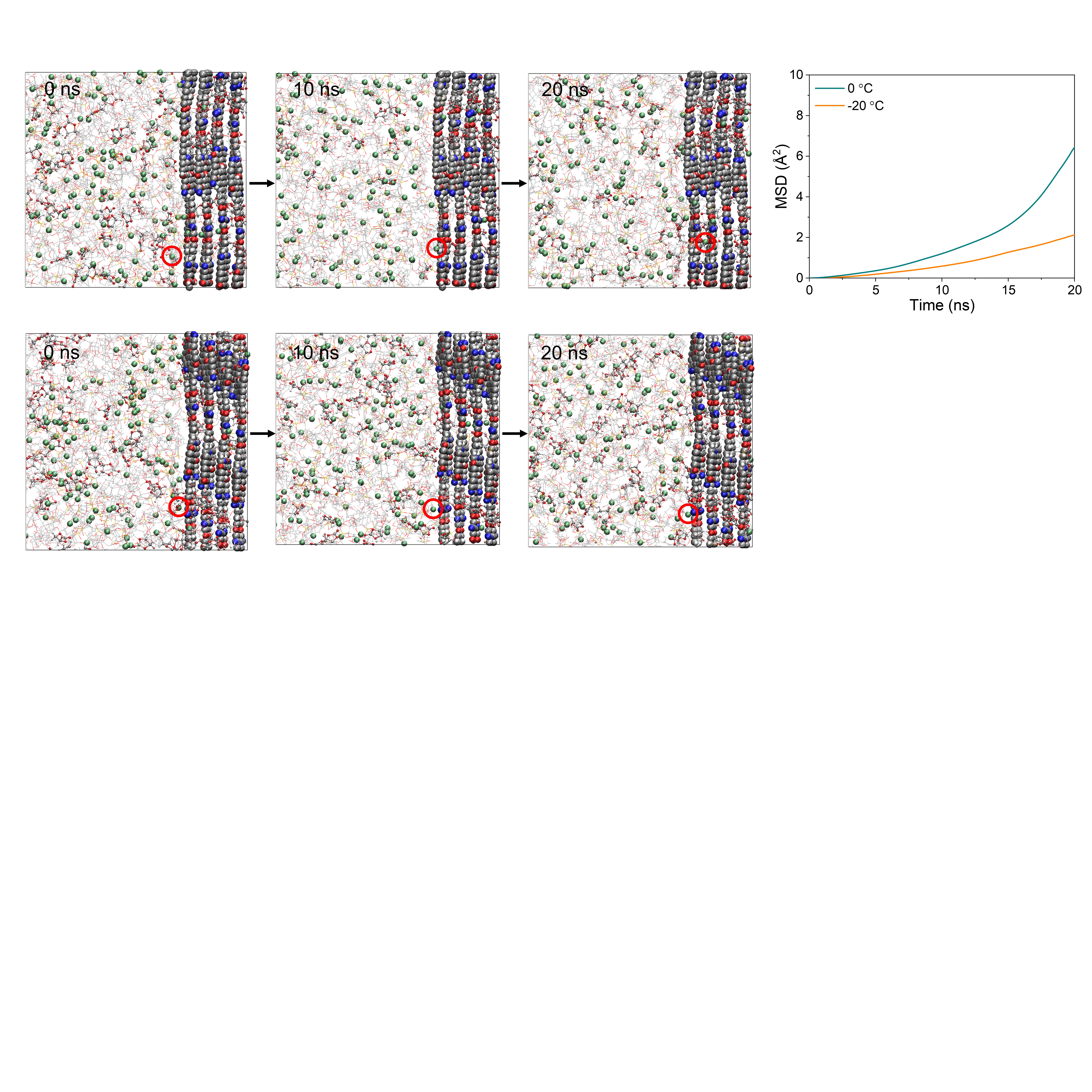


**Figure S29.** Molecular dynamics simulation snapshots of the Li^+^ migration in PLF@COF316 system at -20 °C.


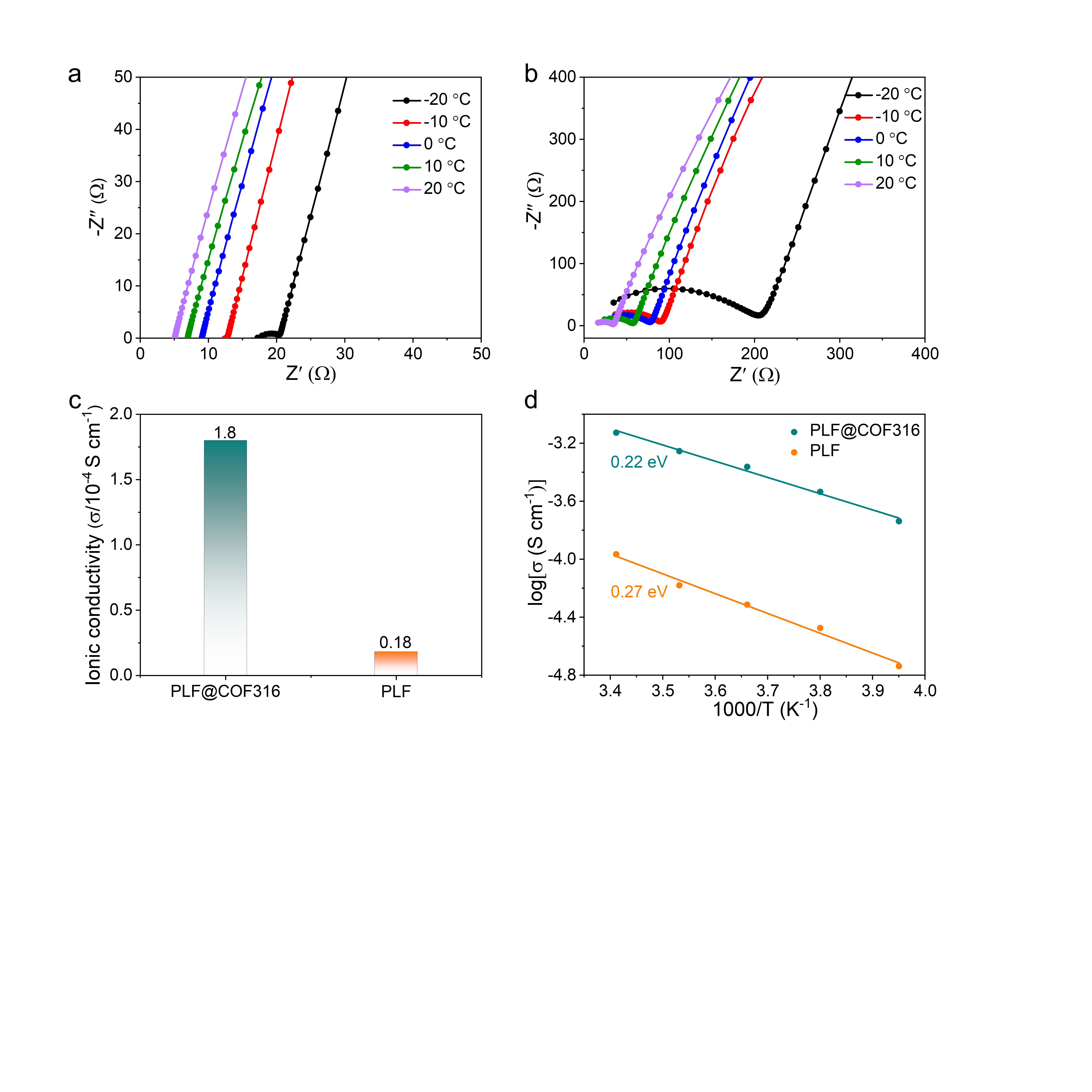


**Figure S30.** EIS curves of (a) PLF@COF316 and (b) PLF from 20 °C to -20 °C. (c) σ_Li+_ and (d) Arrhenius plots of PLF@COF316 and PLF at -20 °C.


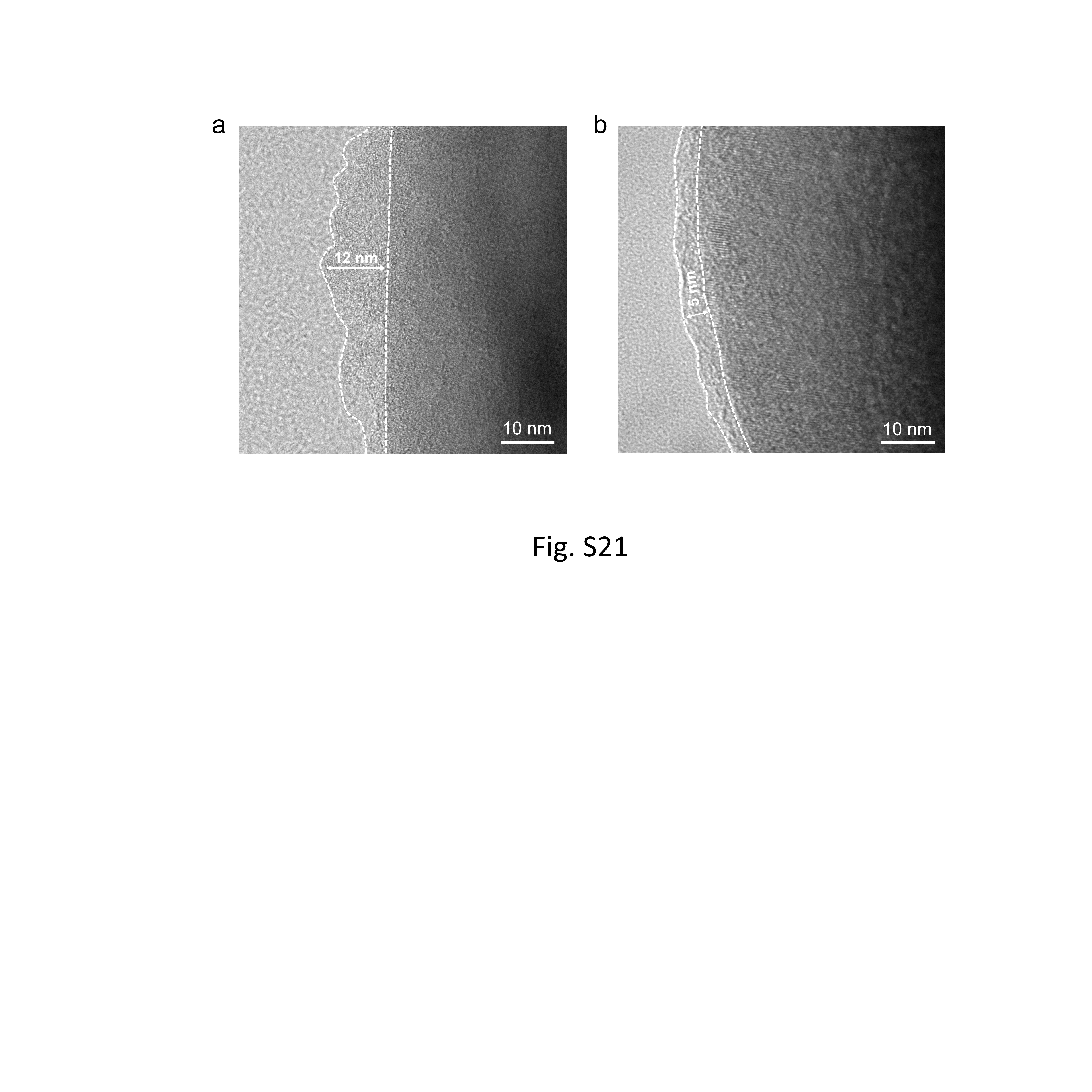


**Figure S31.** TEM images of cycled NCM811 particles with a) PLF and b) PLF@COF316.


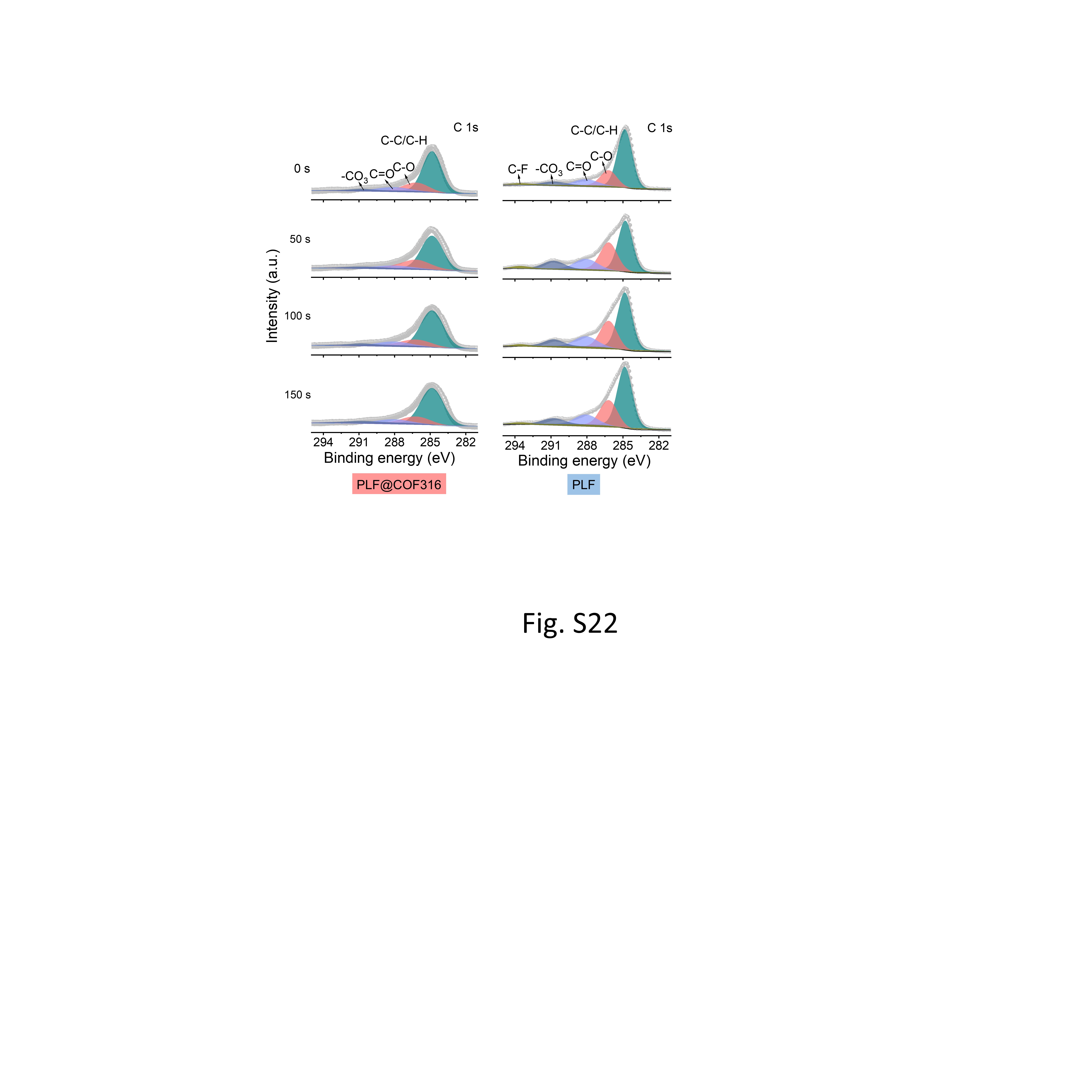


**Figure S32.** XPS C 1s spectra of cycled NCM811 cathodes in the NCM811|PLF@COF316|Li and NCM811|PLF|Li cells.


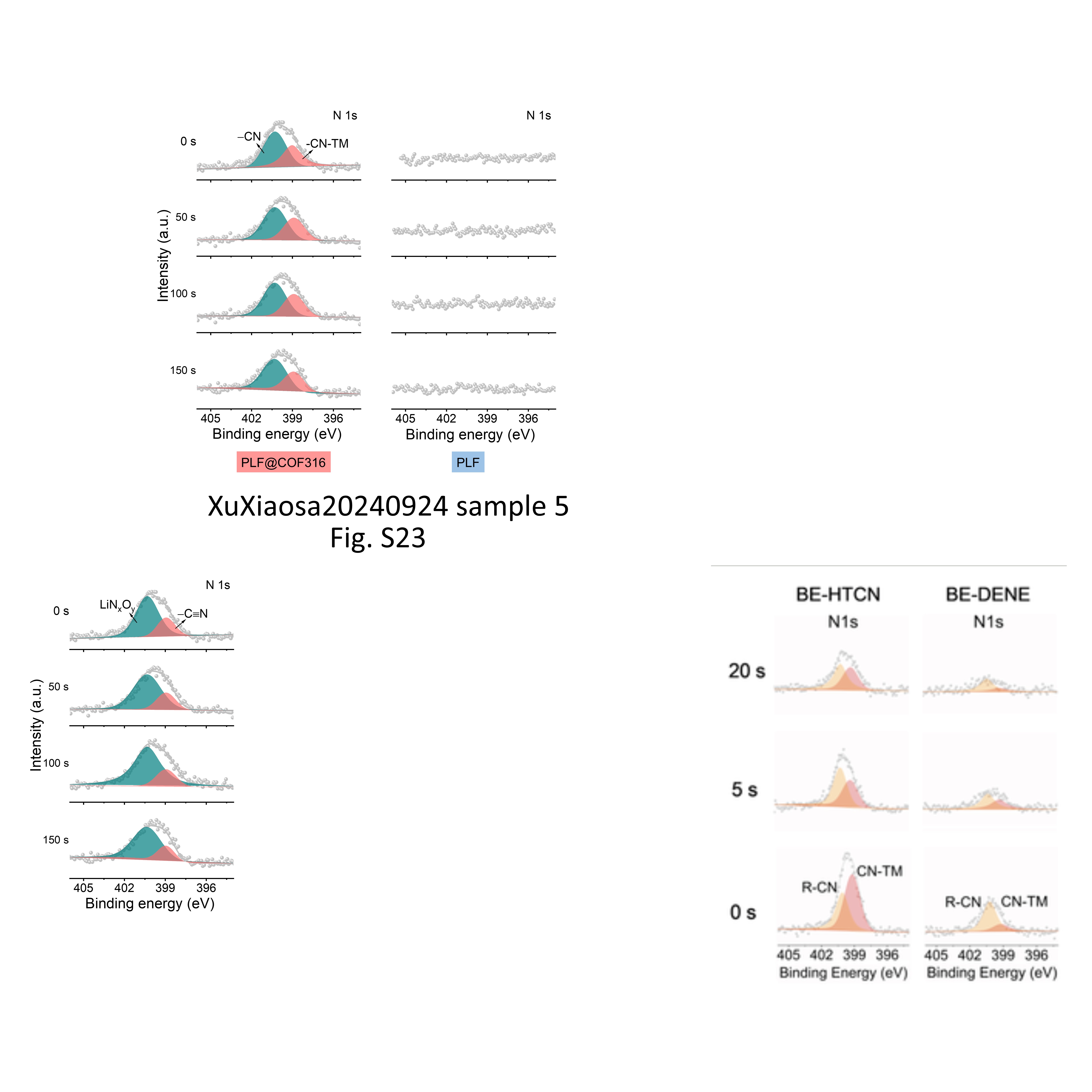


**Figure S33.** XPS N 1s spectra of cycled NCM811 cathodes in the NCM811|PLF@COF316|Li and NCM811|PLF|Li cells.


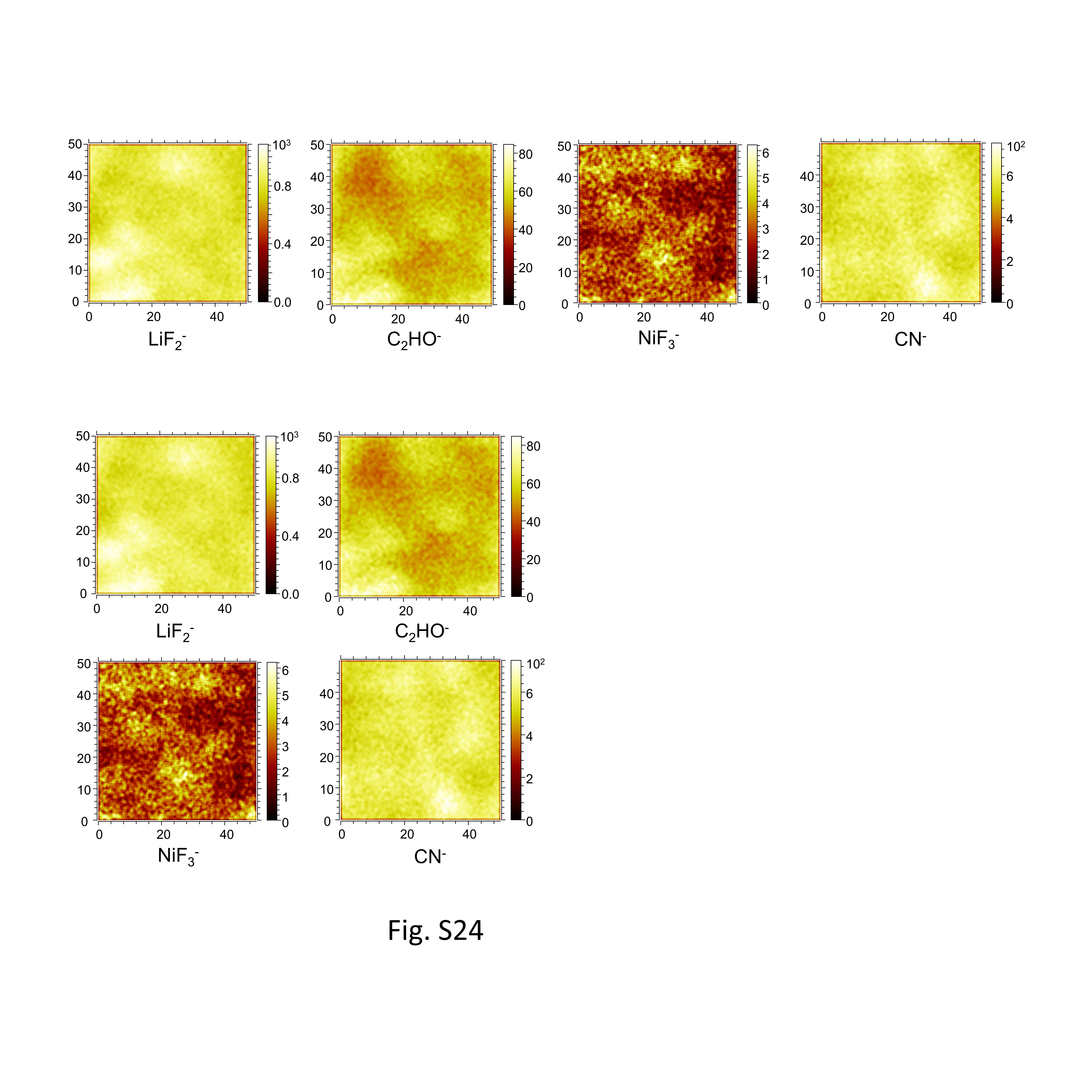


**Figure S34.** TOF-SIMS 2D mappings of LiF_2_^-^, C_2_HO^-^, NiF_3_^-^, and CN^-^ fragments in the formed CEI by PLF@COF316 electrolyte.


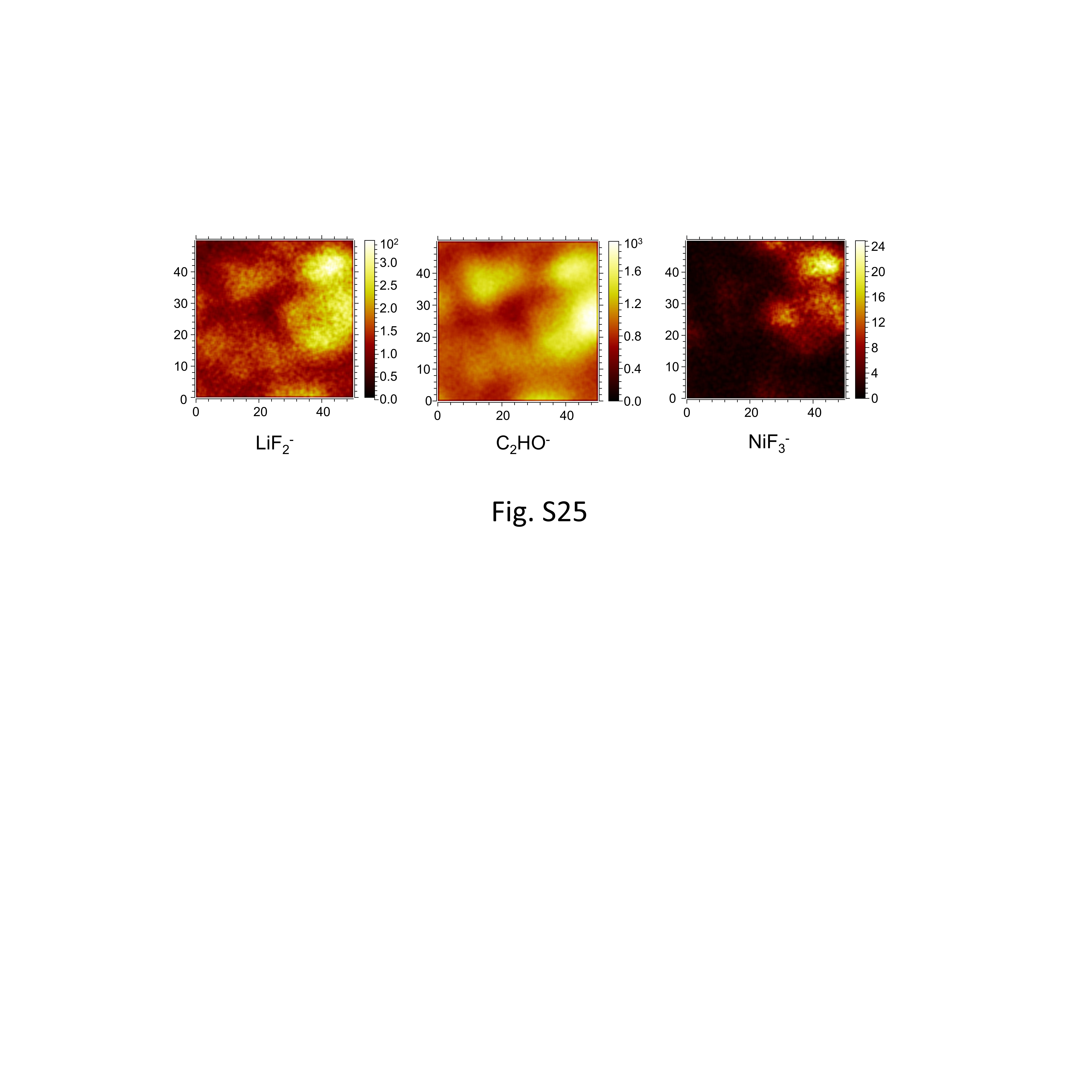


**Figure S35.** TOF-SIMS 2D mappings of LiF_2_^-^, C_2_HO^-^, and NiF_3_^-^ fragments in the formed CEI by PLF electrolyte.


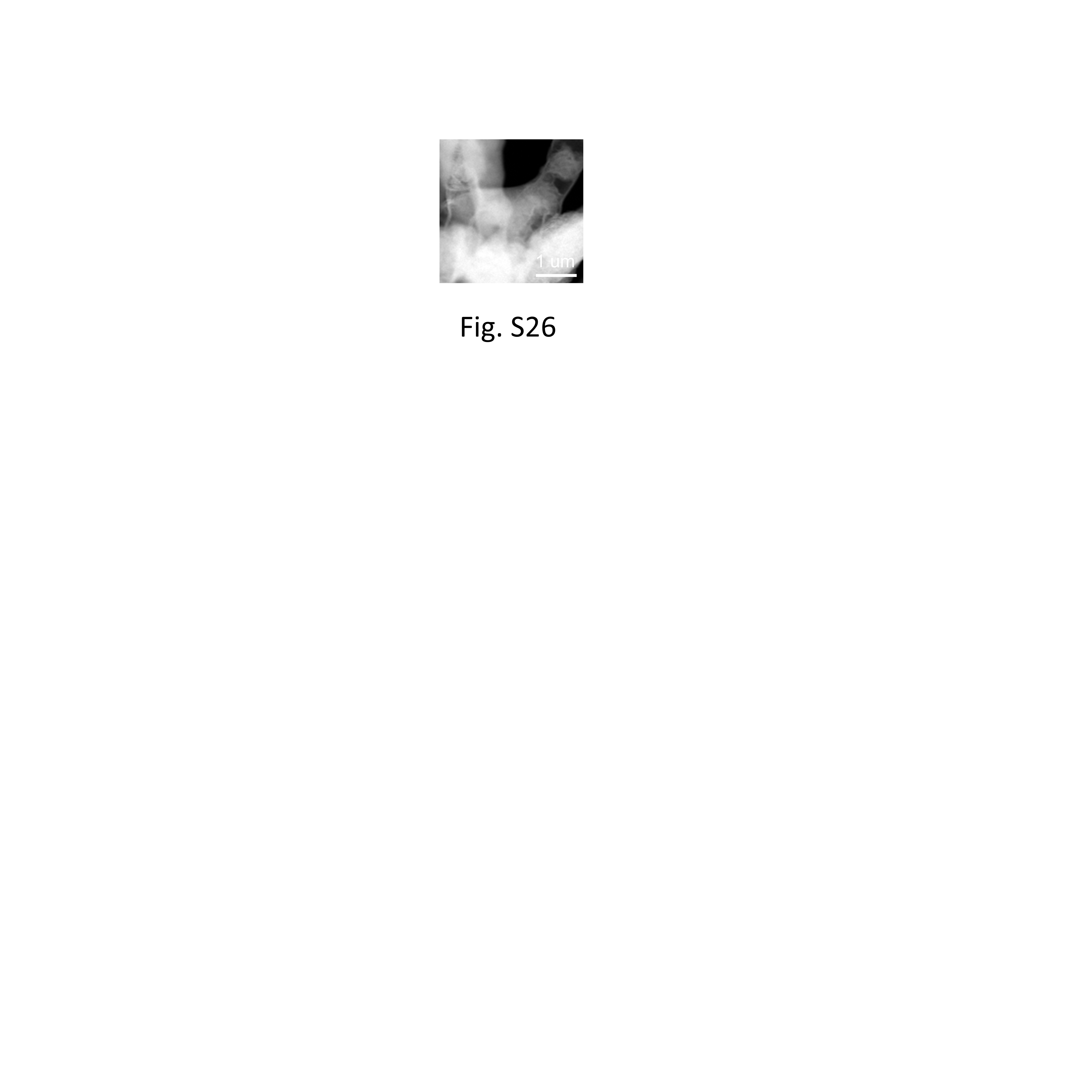


**Figure S36.** The HAADF-STEM image of the deposited Li.


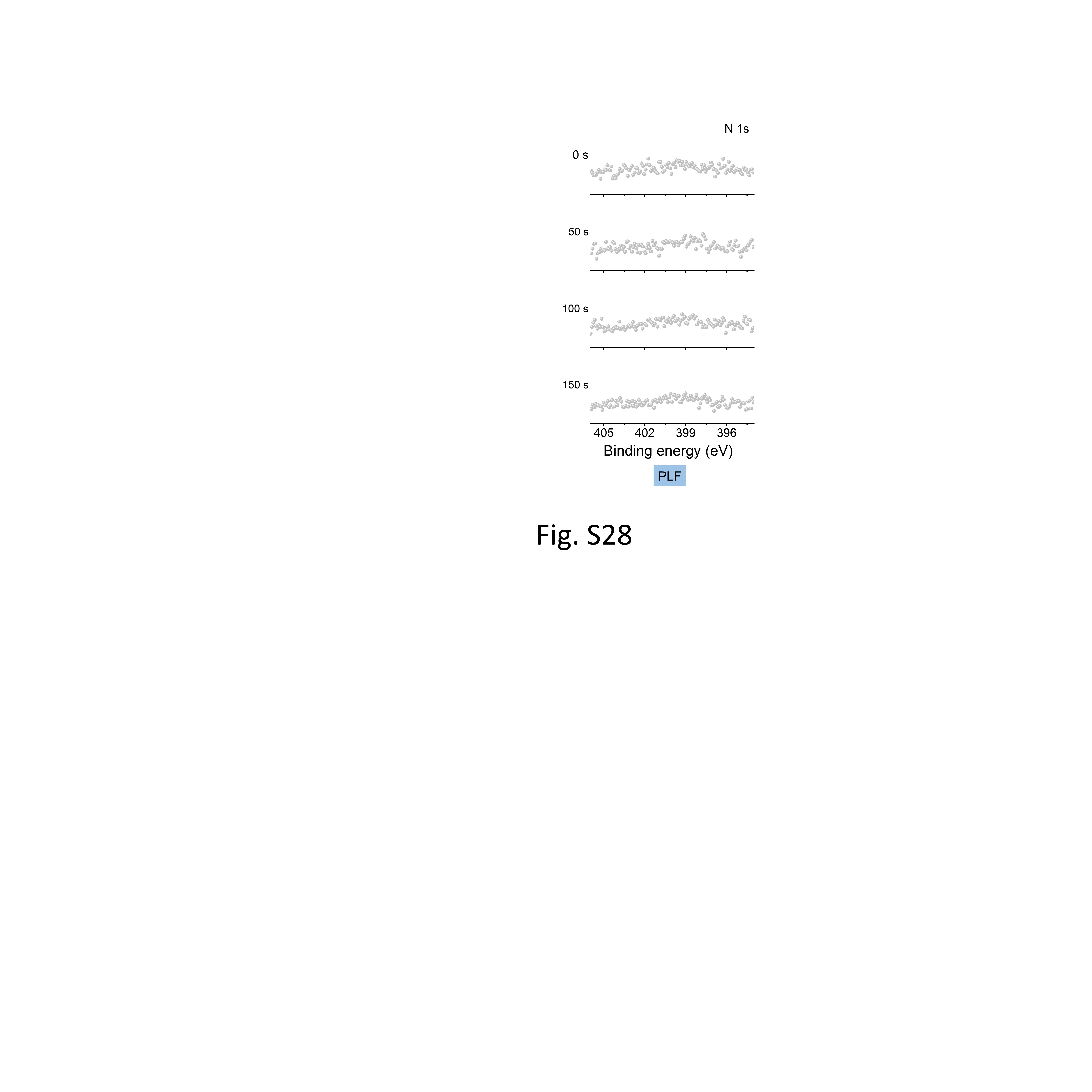


**Figure S37.** XPS depth profiles of N 1s of Li-anodes cycled in NCM811|PLF|Li cell.


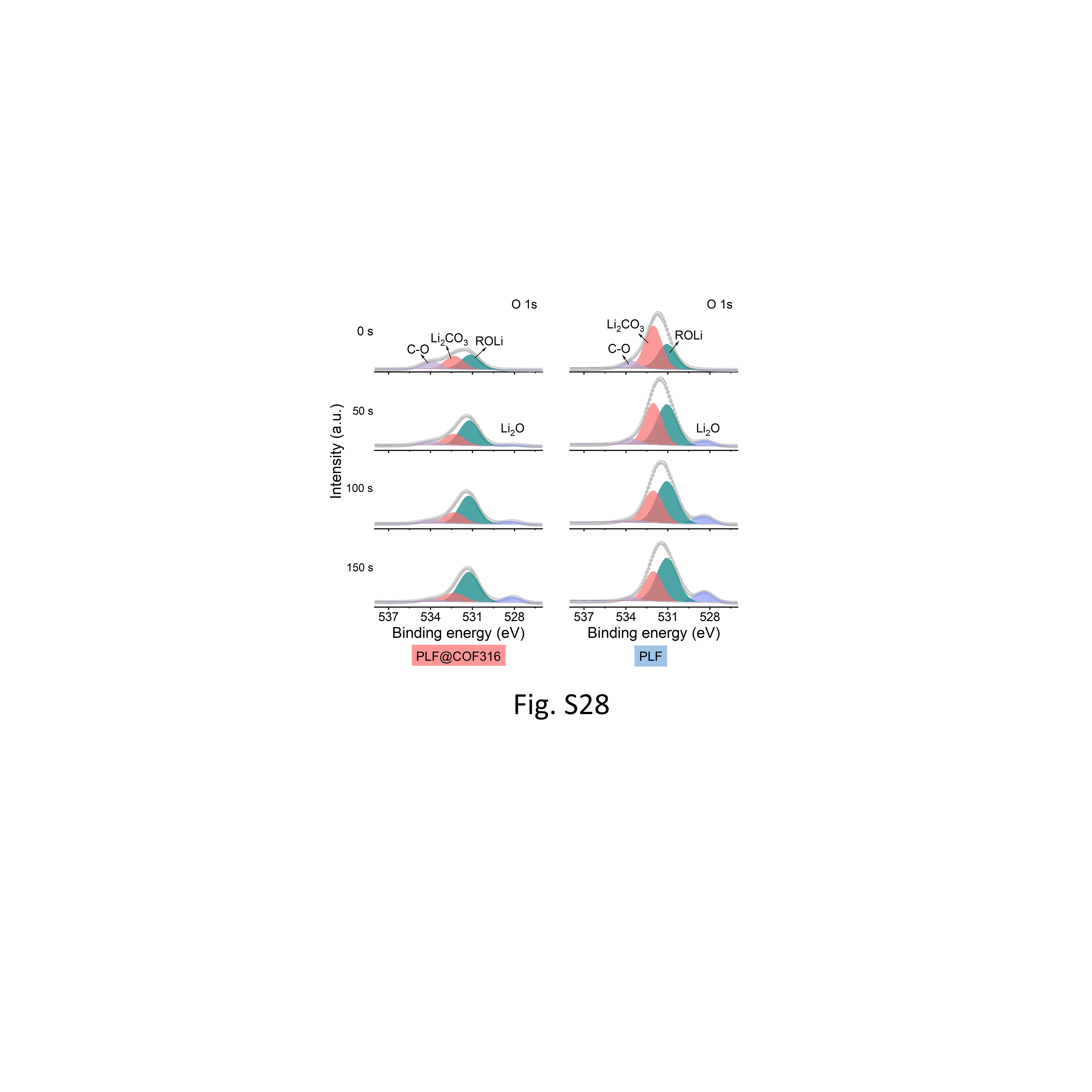


**Figure S38.** XPS depth profiles of O 1s of Li-anodes cycled in NCM811|PLF@COF316|Li and NCM811|PLF|Li cells.


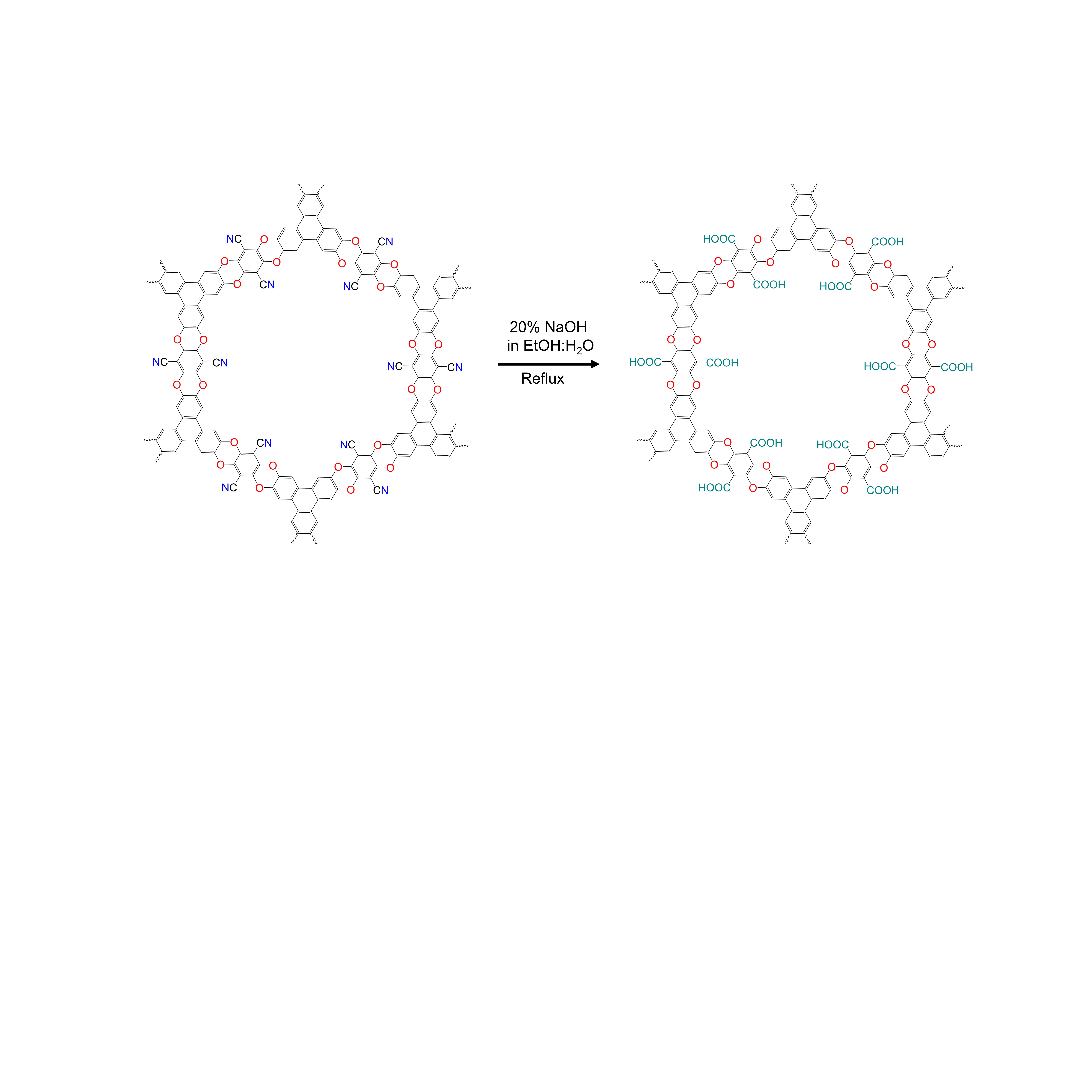


**Figure S39.** The synthesis of COF316-COOH.


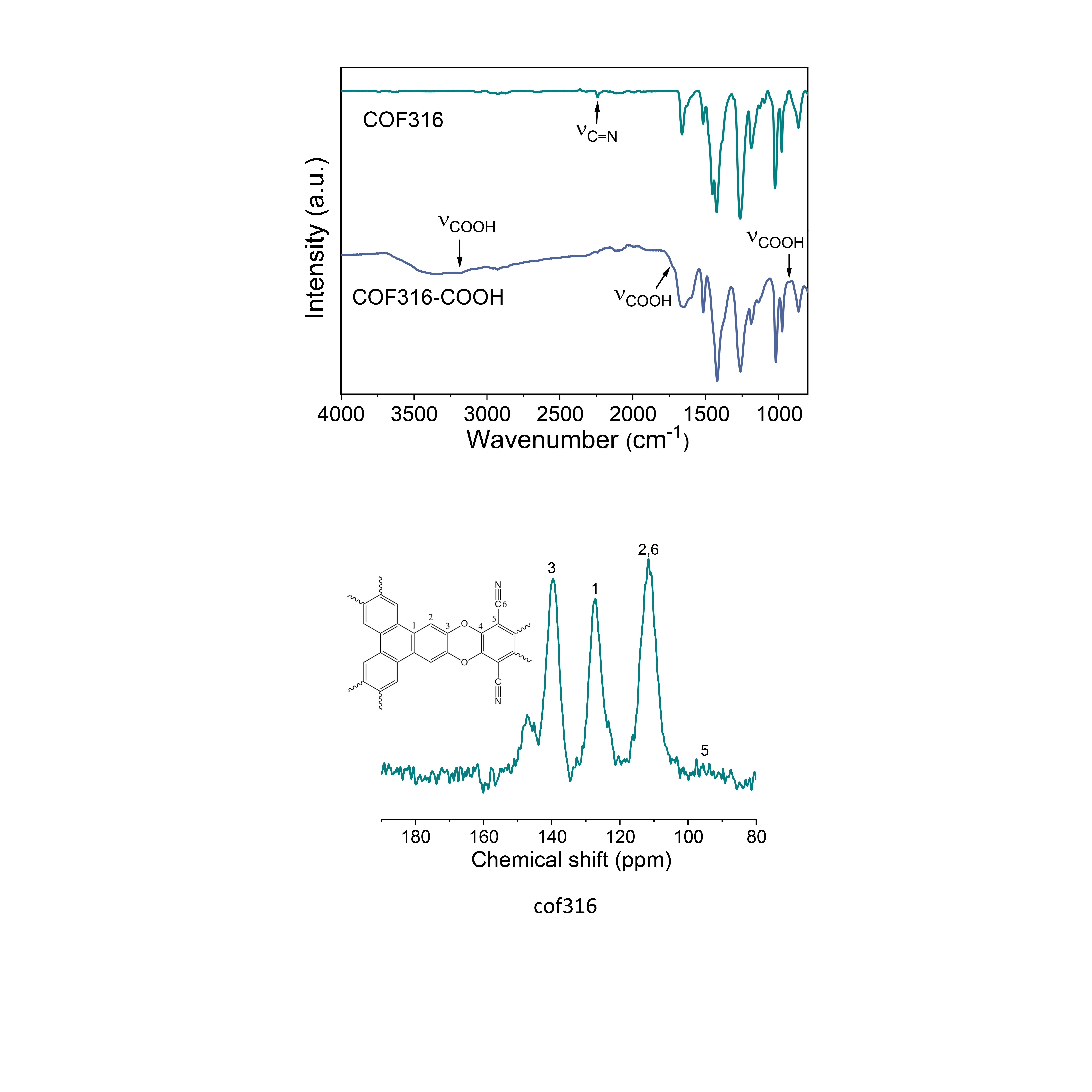


**Figure S40.** FTIR spectra of COF316 and COF316-COOH.

The peaks at 3185 cm^-1^, 1729 cm^-1^, and 924 cm^-1^ for COF316-COOH indicate the successful grafting of the carboxyl-group on the COF316. The significant decrease of the peak at 2240 cm^-1^ confirms the high conversion ratios of cyano-group to carboxyl-group.


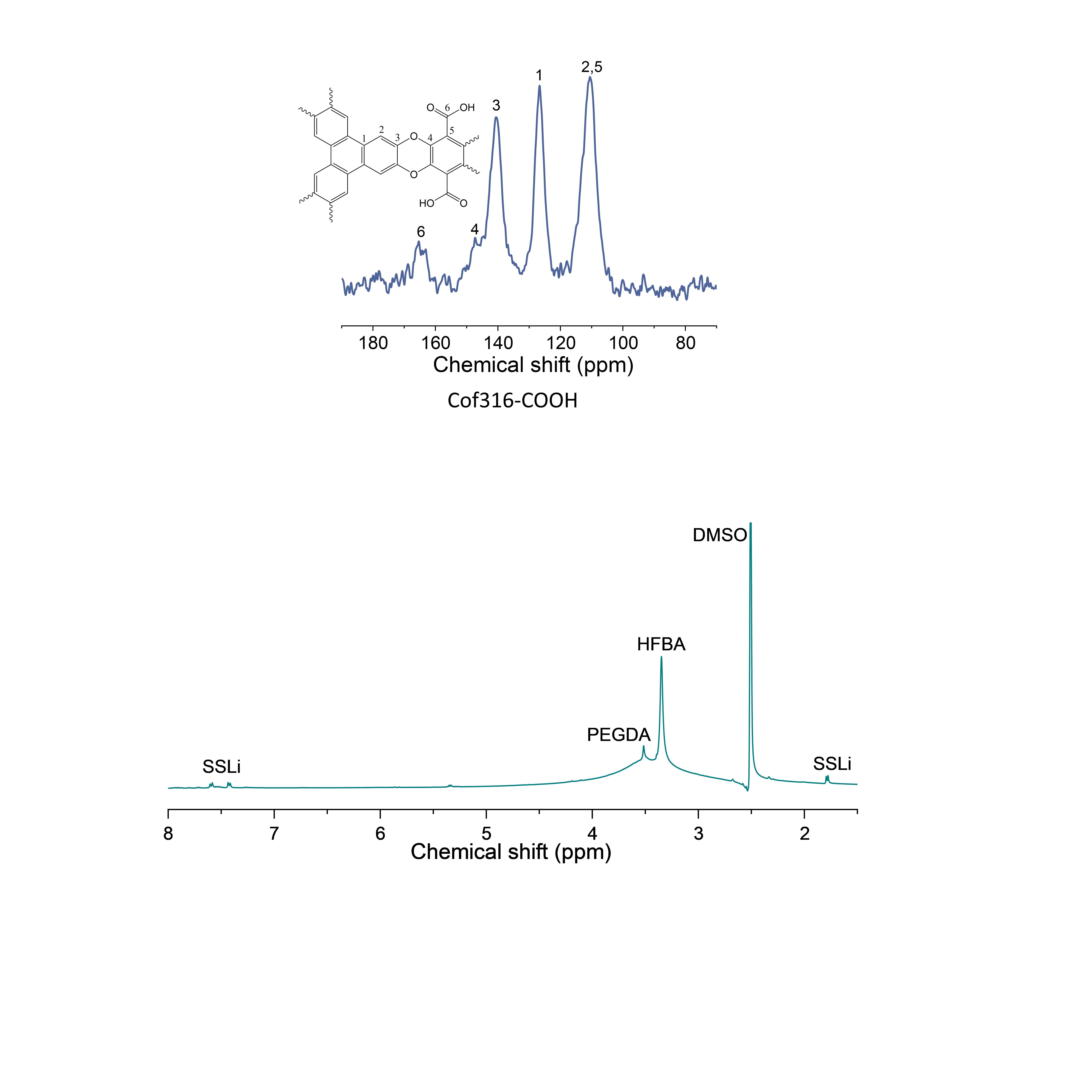


**Figure S41.** Solid-state ^13^C NMR spectrum of COF316-COOH.

The peak at 164 ppm in the solid-state ^13^C NMR spectrum of COF316-COOH demonstrates the presence of carboxyl-group.


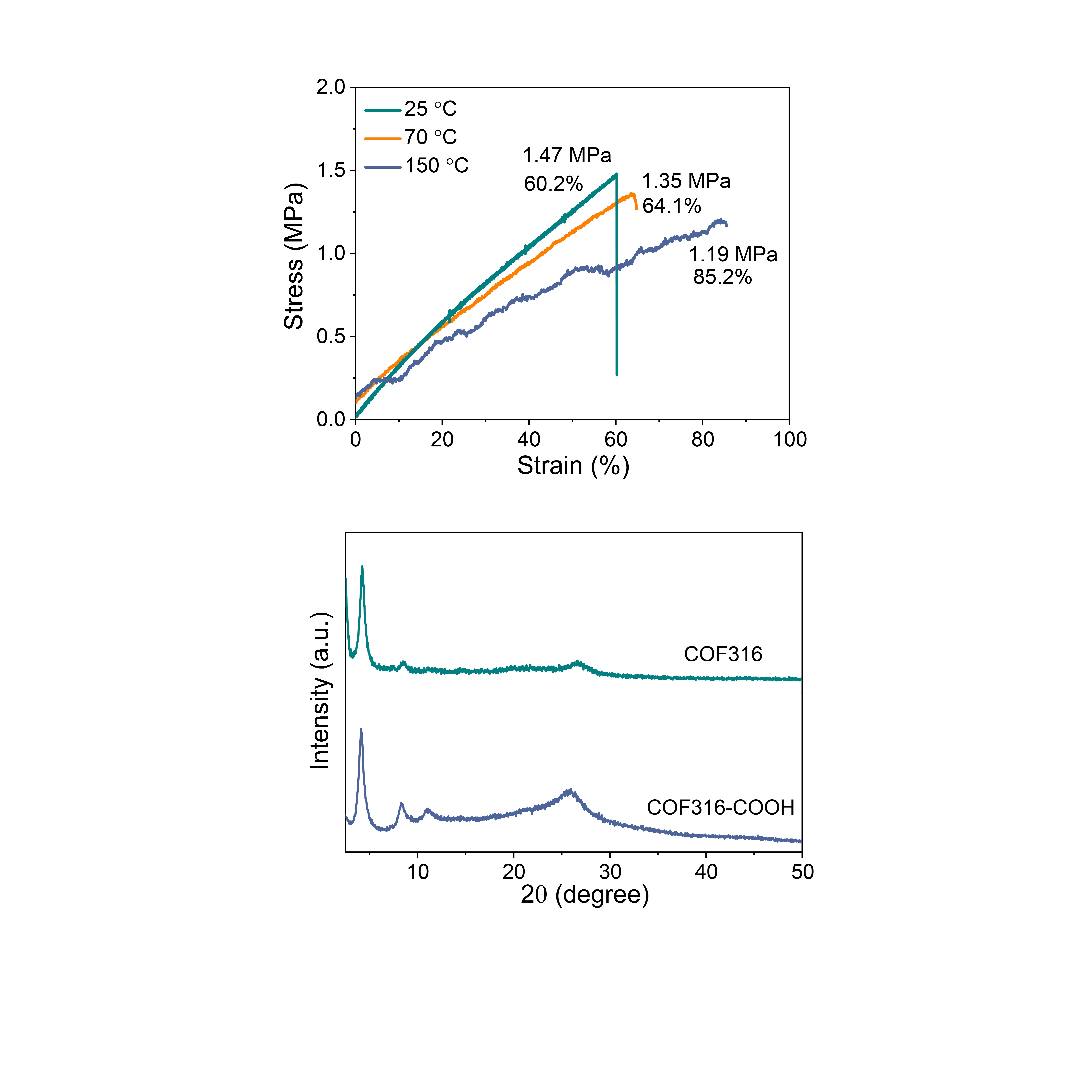


**Figure S42.** XRD patterns of COF316 and COF316-COOH.

The XRD pattern of COF316-COOH is similar to that of COF316, implying that the framework remains unaffected by the grafting of carboxyl-group.


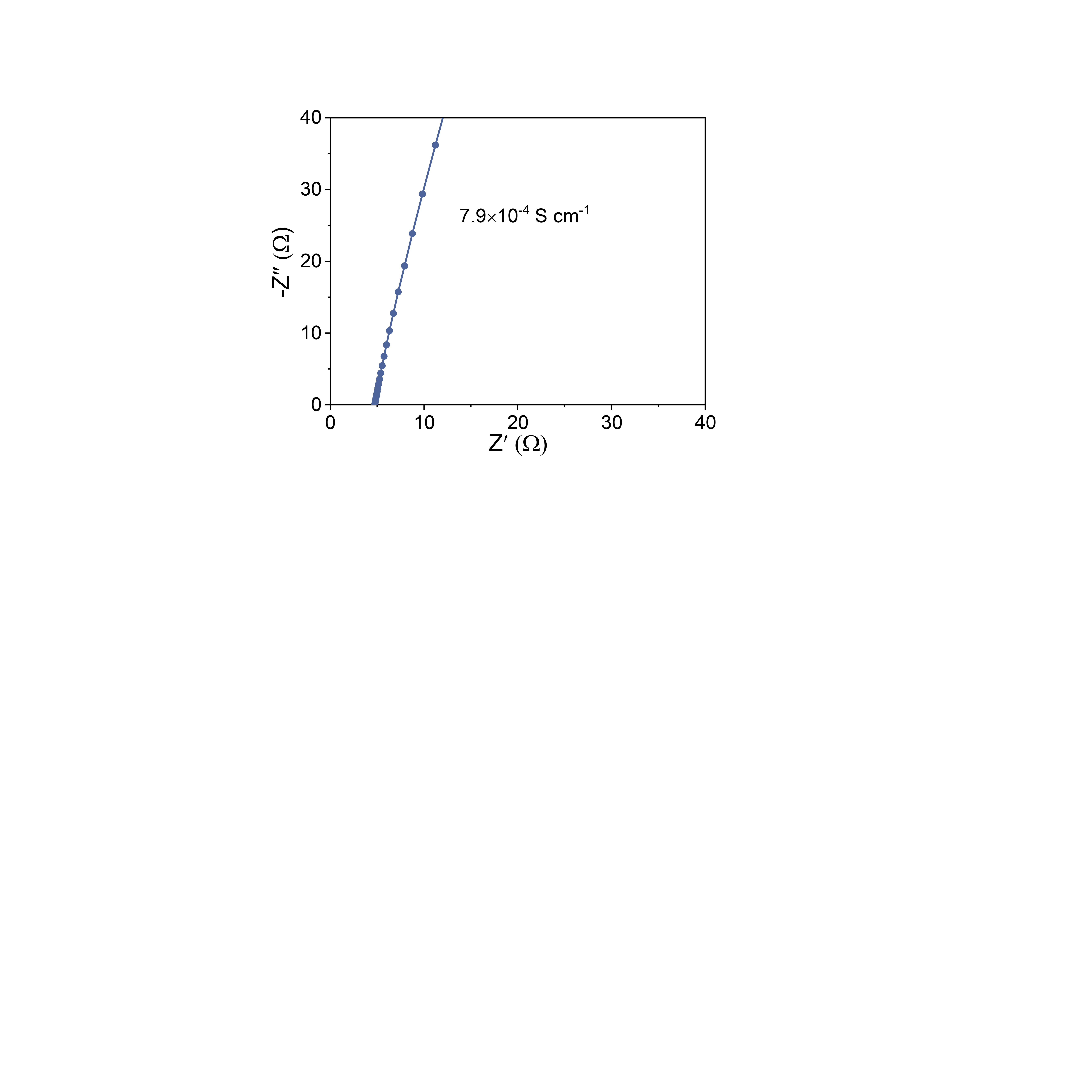


**Figure S43.** EIS curve of PLF@COF316-COOH electrolyte at 30 °C.


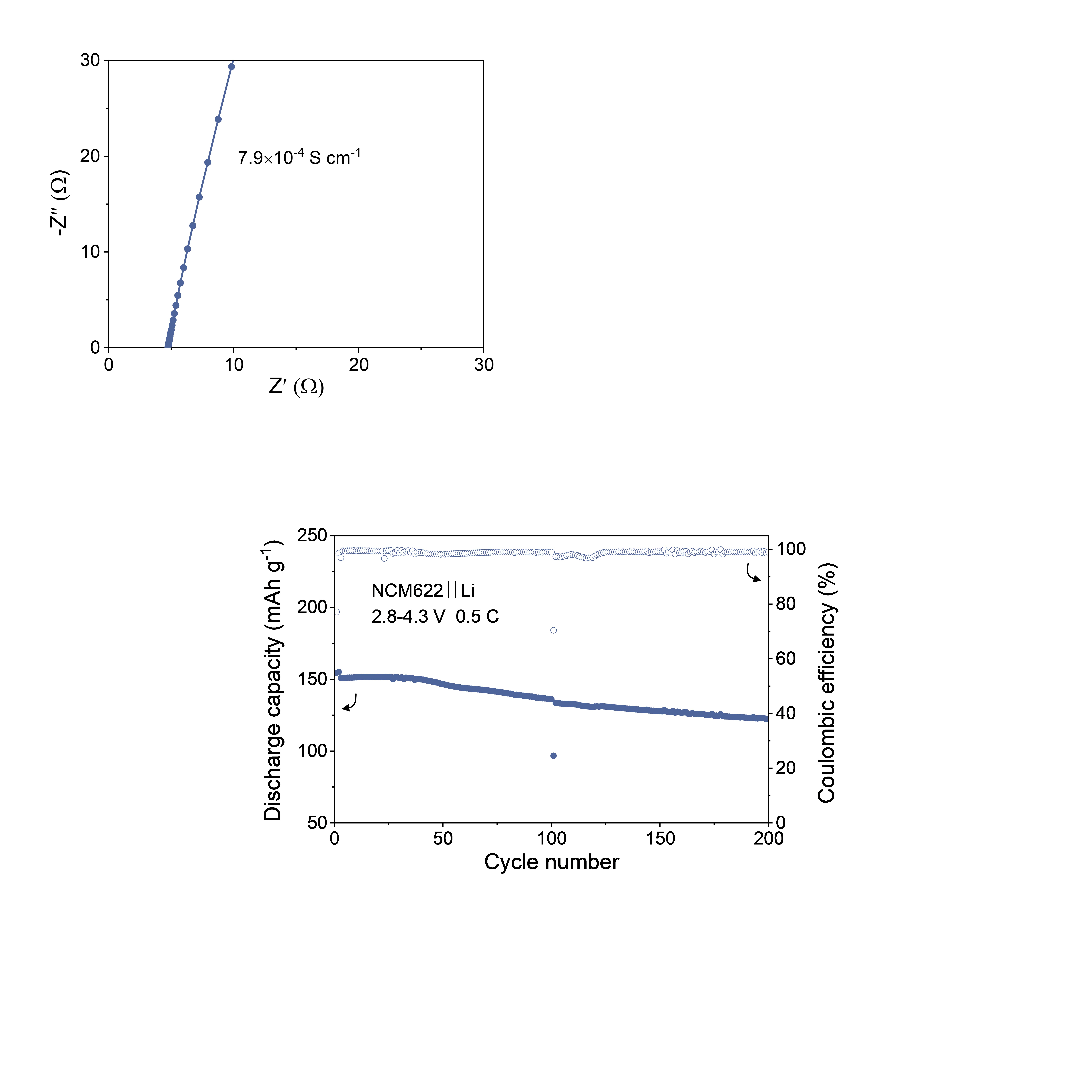


**Figure S44.** Cycling performance of NCM622|PLF@COF316-COOH |Li full cell at 0.5 C.

**Table S1**. Summary of the pore parameters for COF316.

| **Sample** | **S_BET_ (m^2^ g^-1^)** | **Pore volume**  **(cm^3^ g^-1^)** | **Average pore size (nm)** | **Dominant pore size (nm)** |
| --- | --- | --- | --- | --- |
| **COF316** | 337.9 | 0.33 | 3.9 | 1.2 |

**Table S2**. Statistical average results of GPC test.

| **M_p_** | **M_n_** | **M_w_** | **M_z_** | **M_v_** | **PD** |
| --- | --- | --- | --- | --- | --- |
| 926658 | 157893 | 946225 | 2208098 | 794772 | 5.99282 |

**Table S3.** Summary of the impedance fitting data of Li|PLF@COF316|Li and Li|PLF|Li cells.

|  | Sample | R_Bulk_ (Ω) | R_SEI_ (Ω) | R_CT_ (Ω) |
| --- | --- | --- | --- | --- |
| Before cycling | NCM811\|PLF@COF316\|Li | 2.1 | 14.0 | 80.1 |
|  | NCM811\|PLF\|Li | 2.3 | 16.1 | 126.2 |
| After  cycling | NCM811\|PLF@COF316\|Li | 3.6 | 5.0 | 135 |
|  | NCM811\|PLF\|Li | 5.2 | 67.13 | 200.5 |

**Table S4.** Summary of the impedance fitting data of NCM811|PLF@COF316|Li and NCM811|PLF|Li cells.

| Sample | R_Bulk_ (Ω) | R_Interphase_ (Ω) | R_CT_ (Ω) |
| --- | --- | --- | --- |
| NCM811\|PLF@COF316\|Li | 5.0 | 17.0 | 35.2 |
| NCM811\|PLF\|Li | 6.4 | 46.1 | 117.3 |

The charge transfer resistance is the sum of R_Interphase_ and R_CT._

**Table S5.** The overview of NCM811|PLF@COF316|Li pouch cell.

| **Pouch cell overview** | | | |
| --- | --- | --- | --- |
| NCM811 cathode | Specific capacity (mAh g^-1^) | | 205 |
|  | Area loading (mg cm^-2^) | | 10.0 |
|  | Area capacity (mAh cm^-2^) | | 2.1 |
| Li metal anode | Specific capacity (mAh g^-1^) | | 3680 |
|  | Thickness of Li foil (μm) | | 100 |
|  | Area weight (mg cm^-2^) | | 3.74 |
|  | Area capacity (mAh cm^-2^) | | 13.8 |
| Separator (PE) | Thickness (μm) | | 75 |
|  | Areal weight (mg cm^-2^) | | 2 |
| Negative/Positive capacity ratio | | 6.6 | |
| Median voltage (V) | | 3.8 | |
| Cell weight (g) | | 3.5 g | |
| Cell capacity (Ah) | | 0.15 | |
| Energy density (Wh kg^-1^) after 200 cycles | | 282.0 (excluding Al-plastic film) | |

**Table S6.** Comparison of *σ*_Li_^+^, *t*_Li_^+^, cutoff voltage, and cycling performance of PLF@COF316 with reported SICPEs.

| Name | *σ*_Li_^+^  (S cm^-1^) | *t*_Li_^+^ | Specific capacity (mAh g^-1^) and retention | Cycle number | Cutoff voltage (V) | Cathodes | Ref. |
| --- | --- | --- | --- | --- | --- | --- | --- |
| PTF-4EO | 3.53×10^-4^ (RT) | 0.92 | 131.1 (89.8%) | 200 (0.5 C) | 4.25 | LFP | 1 |
| EC_32_-SN | 1.72×10^-4^ (RT) | 0.93 | 139 (93.1%) | 400 (1 C) | 4.0 | LFP | 2 |
| pLBB(OGlyO6FiP) | 1.65×10^-4^ (60 °C) | 0.93 | -- | -- | -- | -- | 3 |
| SIPCE-MOF | 1.14×10^-5^ (RT) | 0.80 | 150 (98.3%) | 120  (0.5 C, 60 °C) | 4.2 | LFP | 4 |
| PEO/GO-PSLi | 2.2×10^-4^ (60 °C) | 0.53 | 134.6 (84.6%) | 250  (1 C, 60 °C) | 4.2 | LFP | 5 |
| SSLMBs with SICSE | 4.0×10^-4^ (RT) | 0.91 | ∼155.2 (84.5%) | 100 (0.1 C) | 4.2 | NCM811 | 6 |
| POSS-LiBMAB | 4.1×10^-4^ (RT) | 0.93 | 116.4 (86.5%) | 700 (1 C) | 4.0 | LFP | 7 |
| ACSLIC | 3.7×10^-5^ (70 °C) | 0.63 | ∼145.1 (98.5%) | 30  (0.1 C, 70 °C) | 3.7 | LFP | 8 |
| ANP-5 | 1.5×10^-4^ (RT) | 0.95 | 102 (84%) | 100 (0.5 C) | 3.8 | LFP | 9 |
| **PLF@COF316** | **9.2×10^-4^ (RT)** | **0.94** | **139.4 (92.0%)** | **1000**  **(0.5 C)** | **4.3** | **NCM622** | **This work** |

**Table S7.** Summary of XPS curve fitting results of cycled NCM811 cathodes in the NCM811|PLF@COF316|Li and NCM811|PLF|Li cells.

| Spectra  details | Binding  energy (eV) | Attributed species | FWHM (eV) | | | | | | | |
| --- | --- | --- | --- | --- | --- | --- | --- | --- | --- | --- |
|  |  |  | PLF@COF316 | | | | PLF | | | |
|  |  |  | 0 s | 50 s | 100 s | 150 s | 0 s | 50 s | 100 s | 150 s |
| C 1s | 284.8 | C-C/C-H | 1.9 | 2.1 | 2.3 | 2.3 | 1.5 | 1.4 | 1.4 | 1.5 |
|  | 286.2 | C-O | 2.5 | 3.0 | 3.1 | 2.8 | 1.6 | 1.7 | 1.7 | 1.7 |
|  | 288.1 | C=O | 3.0 | 3.5 | 3.1 | 3.1 | 2.2 | 2.2 | 2.2 | 2.2 |
|  | 290.7 | -CO_3_ | 2.2 | 3.4 | 2.7 | 3.6 | 2.3 | 2.1 | 1.9 | 2.2 |
|  | 293.5 | C-F | / | / | / | / | 2.3 | 1.8 | 1.4 | 1.7 |
| F 1s | 685.0 | LiF | 1.8 | 1.9 | 1.9 | 1.9 | 3.3 | 3.0 | 2.3 | 2.5 |
|  | 687.5 | C-F | 2.8 | 2.7 | 2.7 | 2.7 | 2.2 | 2.4 | 2.7 | 2.6 |
| N 1s | 398.9 | -CN-TM | 1.7 | 1.8 | 1.8 | 1.7 | / | / | / | / |
|  | 400.3 | -CN | 2.0 | 2.0 | 2.0 | 2.1 | / | / | / | / |
| Ni 2p | 849.9 | Ni^0^ | / | / | / | / | 3.0 | 3.1 | 2.9 | 2.4 |
|  | 851.0 | Ni^2+^ (2p_3/2_) | 6.3 | 5.4 | 6.1 | 6.0 | 6.6 | 4.8 | 5.3 | 6.6 |
|  | 857.5 | Ni^3+^ (2p_3/2_) | 7.4 | 6.7 | 7.0 | 7.1 | 3.5 | 3.6 | 3.5 | 3.8 |
|  | 875.7 | Ni^2+^ (2p_1/2_) | 4.0 | 4.5 | 5.0 | 6.0 | 5.0 | 3.7 | 4.0 | 4.9 |
|  | 878.2 | Ni^3+^ (2p_1/2_) | 5.1 | 5.5 | 5.5 | 6.0 | 5.5 | 5.0 | 6.0 | 6.6 |

**Table S8.** The LiF, organic C, and reduced Ni ions contents in the CEI with PLF@COF316 (up) and PLF (bottom) electrolytes.

| Etching time (s) | LiF (%) | organic C (%) | Reduced Ni ions (%) |
| --- | --- | --- | --- |
| 0 | 11.17 | 61.58 | 0.26 |
|  | 2.74 | 66.49 | 0.41 |
| 50 | 12.63 | 60.98 | 0.23 |
|  | 5.49 | 65.33 | 0.55 |
| 100 | 13.13 | 59.78 | 0.19 |
|  | 4.92 | 69.73 | 0.52 |
| 150 | 13.65 | 59.98 | 0.25 |
|  | 4.07 | 72.33 | 0.35 |

**Table S9.** Summary of XPS curve fitting results of Li-anodes cycled in NCM811|PLF@COF316|Li and NCM811|PLF|Li cells.

| Spectra  details | Binding  energy (eV) | Attributed species | FWHM (eV) | | | | | | | |
| --- | --- | --- | --- | --- | --- | --- | --- | --- | --- | --- |
|  |  |  | PLF@COF316 | | | | PLF | | | |
|  |  |  | 0 s | 50 s | 100 s | 150 s | 0 s | 50 s | 100 s | 150 s |
| C 1s | 284.8 | C-C/C-H | 1.4 | 1.8 | 1.8 | 2.0 | 1.6 | 1.8 | 1.9 | 2.1 |
|  | 286.2 | C-O | 1.4 | 1.8 | 1.5 | 1.8 | 1.9 | 1.8 | 1.8 | 1.9 |
|  | 288.1 | C=O | 1.7 | 2.1 | 1.6 | 1.9 | 1.6 | 1.5 | 1.4 | 1.5 |
|  | 289.4 | O-C=O | 1.3 | 1.3 | 1.2 | 1.3 | 1.0 | 1.1 | 1.1 | 1.1 |
|  | 290.2 | -CO_3_ | 1.1 | 1.1 | 1.0 | 1.2 | 1.7 | 1.7 | 1.5 | 1.3 |
|  | 293.3 | C-F | / | / | / | / | 1.1 | 1.0 | 1.1 | 1.1 |
| F 1s | 685.0 | LiF | 1.8 | 1.7 | 1.6 | 1.6 | 2.6 | 1.7 | 1.7 | 1.7 |
|  | 688.1 | C-F | 2.8 | / | / | / | 2.2 | 2.5 | 2.7 | 2.7 |
| N 1s | 400.0 | Li_3_N | 1.9 | 2.3 | 2.1 | 2.2 | / | / | / | / |
| O 1s | 528.3 | Li_2_O | / | 2.2 | 2.0 | 1.5 | / | 1.5 | 1.6 | 1.5 |
|  | 531.1 | ROLi | 1.7 | 1.7 | 1.6 | 1.7 | 1.6 | 1.7 | 1.8 | 1.8 |
|  | 532.2 | Li_2_CO_3_ | 1.6 | 1.7 | 1.7 | 1.8 | 1.5 | 1.5 | 1.6 | 1.5 |
|  | 533.8 | C-O | 1.6 | 1.7 | 1.6 | 1.5 | 1.3 | 1.4 | 2.1 | 1.3 |

**References**

(1) Li, H.; Du, Y.; Zhang, Q.; Zhao, Y.; Lian, F. A single‐ion conducting network as rationally coordinating polymer electrolyte for solid‐state Li metal batteries. *Adv. Energy Mater.* **2022**, *12* (13), 2103530. DOI: 10.1002/aenm.202103530.

(2) Wen, K.; Xin, C.; Guan, S.; Wu, X.; He, S.; Xue, C.; Liu, S.; Shen, Y.; Li, L.; Nan, C. W. Ion-dipole interaction regulation enables high-performance single-ion polymer conductors for solid-state batteries. *Adv. Mater.* **2022**, *34* (32), 2202143. DOI: 10.1002/adma.202202143.

(3) Guzman-Gonzalez, G.; Vauthier, S.; Alvarez-Tirado, M.; Cotte, S.; Castro, L.; Gueguen, A.; Casado, N.; Mecerreyes, D. Single-ion lithium conducting polymers with high ionic conductivity based on borate pendant groups. *Angew. Chem. Int. Ed.* **2022**, *61* (7), e202114024. DOI: 10.1002/anie.202114024.

(4) Chen, L.; Xue, P.; Liang, Q.; Liu, X.; Tang, J.; Li, J.; Liu, J.; Tang, M.; Wang, Z. A single-ion polymer composite electrolyte via in situ polymerization of electrolyte monomers into a porous MOF-based fibrous membrane for lithium metal batteries. *ACS Appl. Energy Mater.* **2022**, *5* (3), 3800-3809. DOI: 10.1021/acsaem.2c00282.

(5) Hu, Z.; Bao, W.; Zhang, Y.; Jiang, X.; Chen, J.; Zhang, Y.; Huo, S.; Zhang, J.; Qin, J.; Li, S.; et al. Single-ion conductors functionalized graphene oxide enabling solid polymer electrolytes with uniform Li-ion transport toward stable and dendrite-free lithium metal batteries. *Chem. Eng. J.* **2023**, *472*, 144932. DOI: 10.1016/j.cej.2023.144932.

(6) Oh, K. S.; Kim, J. H.; Kim, S. H.; Oh, D.; Han, S. P.; Jung, K.; Wang, Z.; Shi, L.; Su, Y.; Yim, T.; et al. Single‐ion conducting soft electrolytes for semi‐solid lithium metal batteries enabling cell fabrication and operation under ambient conditions. *Adv. Energy Mater.* **2021**, *11* (38), 2101813. DOI: 10.1002/aenm.202101813.

(7) Liu, P.; Zhang, J.; Zhong, L.; Huang, S.; Gong, L.; Han, D.; Wang, S.; Xiao, M.; Meng, Y. Interphase building of organic-inorganic hybrid polymer solid electrolyte with uniform intermolecular Li^+^ path for stable lithium metal batteries. *Small* **2021**, *17* (41), 2102454. DOI: 10.1002/smll.202102454.

(8) Martinez‐Ibañez, M.; Sanchez‐Diez, E.; Qiao, L.; Zhang, Y.; Judez, X.; Santiago, A.; Aldalur, I.; Carrasco, J.; Zhu, H.; Forsyth, M.; et al. Unprecedented improvement of single Li‐ion conductive solid polymer electrolyte through salt additive. *Adv. Funct. Mater.* **2020**, *30* (16), 2000455. DOI: 10.1002/adfm.202000455.

(9) Shin, D. M.; Bachman, J. E.; Taylor, M. K.; Kamcev, J.; Park, J. G.; Ziebel, M. E.; Velasquez, E.; Jarenwattananon, N. N.; Sethi, G. K.; Cui, Y.; et al. A single-ion conducting borate network polymer as a viable quasi-solid electrolyte for lithium metal batteries. *Adv. Mater.* **2020**, *32* (10), 1905771. DOI: 10.1002/adma.201905771.
